# Supplementary material for: Synthesis and Evaluation of Antimicrobial and Cytotoxic Activity of Oxathiine-Fused Quinone-Thioglucoside Conjugates of Substituted 1,4-Naphthoquinones
Source: Molecules. 2020 Aug 6;25(16):3577. doi: 10.3390/molecules25163577 (PMC7463537; doi:10.3390/molecules25163577)

Supplementary Materials

for the paper

"Synthesis and evaluation of antimicrobial and cytotoxic activity of oxathiine fused quinone-thioglicoside conjugates of substituted 1,4-naphthoquinones"

Yuri E. Sabutski, Ekaterina S. Menchinskaya, Ludmila S. Shevchenko, Ekaterina A. Chingizova, Artur R. Chingizov, Roman S. Popov, Vladimir A. Denisenko, Valery V. Michailov, Dmitry L. Aminin, Sergey G. Polonik

Content

^{1}H and ^{13}C NMR spectra for synthesized compounds S2-S33

2-(2,3,4,6-Tetra-O-acetyl-β-D-glucopyranosyl-1-thio)-3,5,8-trimethoxy-1,4-naphthoquinone ***(13a)****, (^{1}H NMR - 500 MHz, ^{13}C NMR - 125 MHz, solvent - $CDCl_3$)*

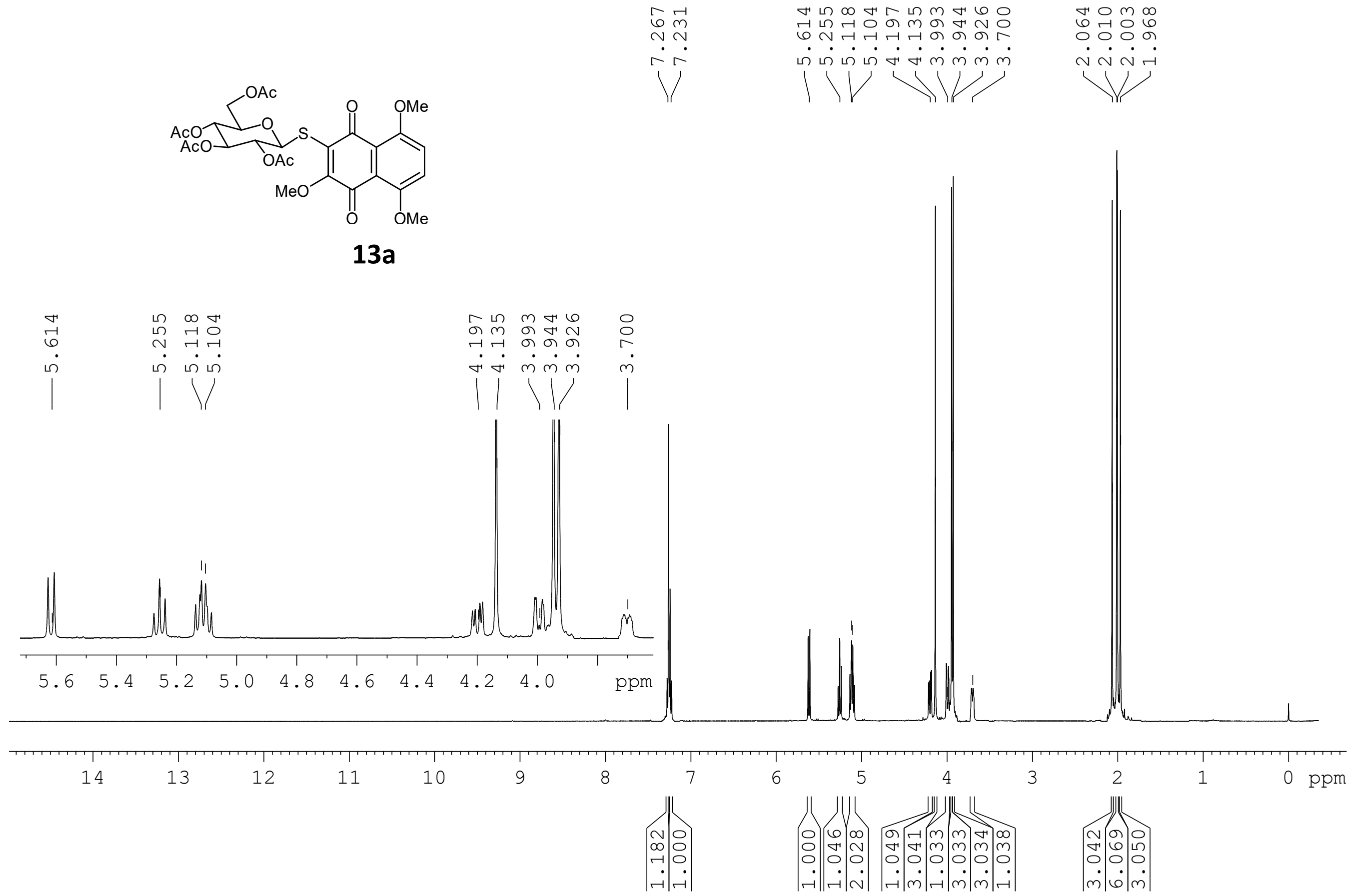

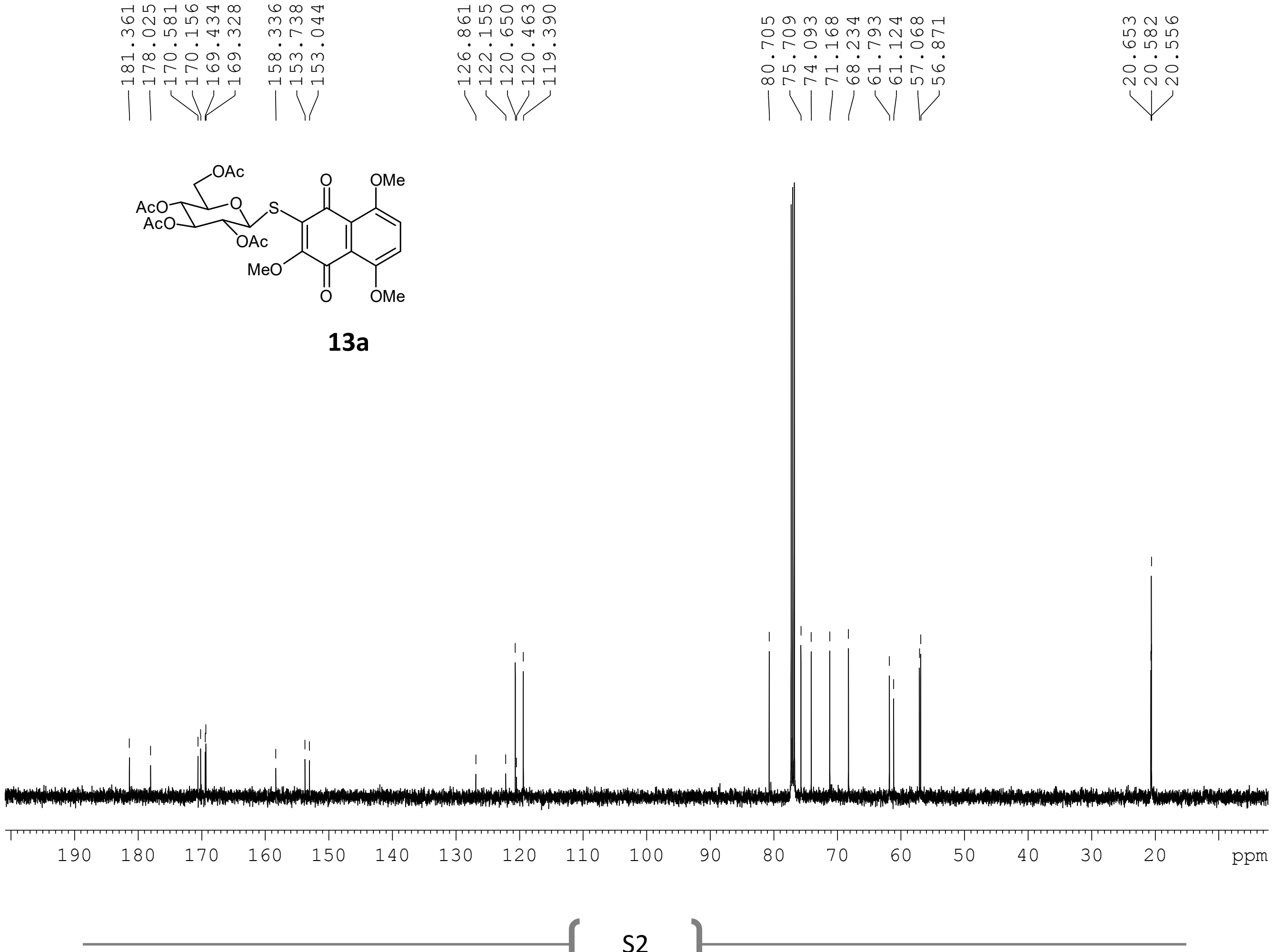

2-(2,3,4,6-Tetra-O-acetyl-β-D-galactopyranosyl-1-thio)-3,5,8-trimethoxy-1,4-naphthoquinone ***(13b)****, (^{1}H NMR - 500 MHz, ^{13}C NMR - 125 MHz, solvent - $CDCl_3$)*

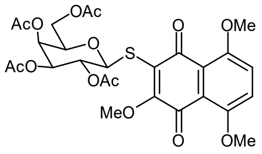

13b

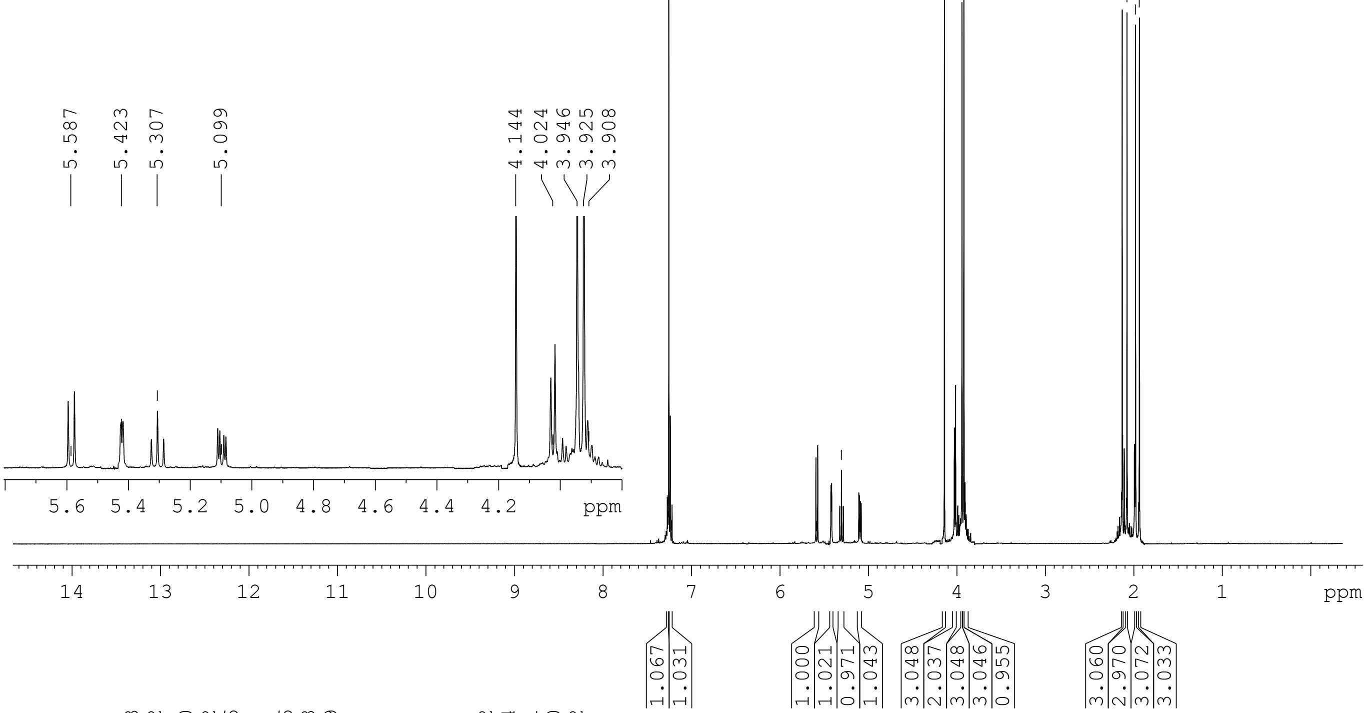

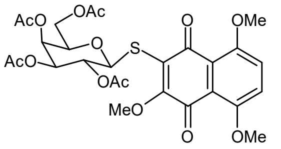

13b

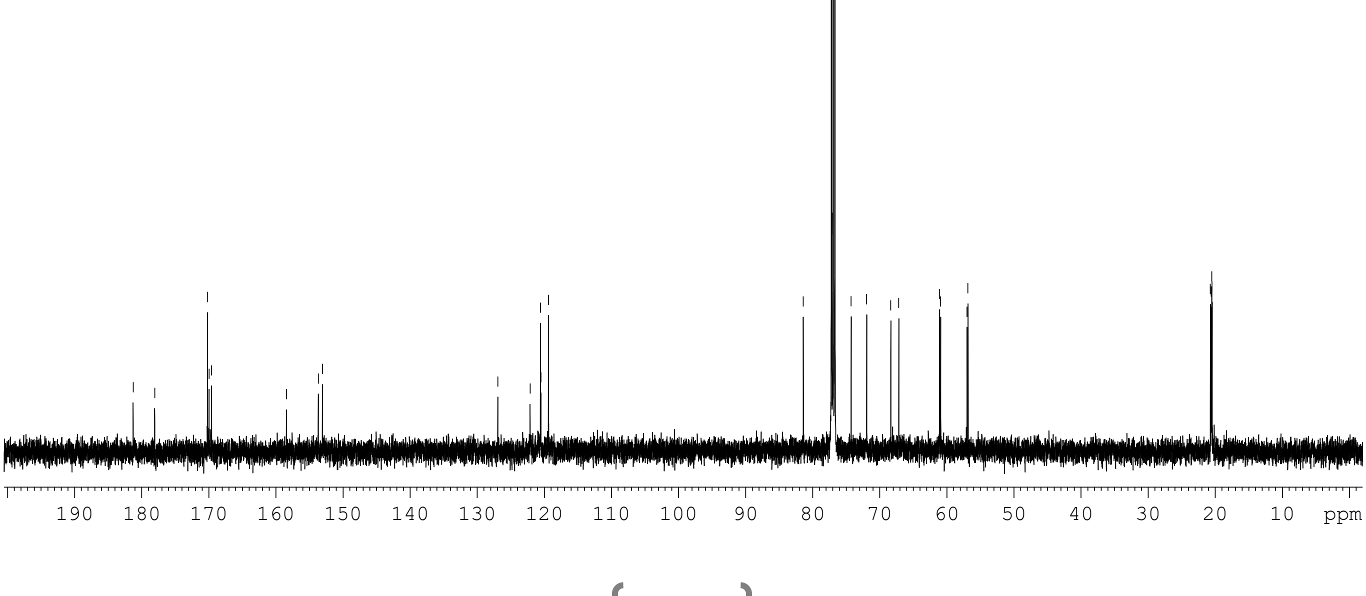

2-(2,3,4-Tri-O-acetyl-β-D-xylopyranosyl-1-thio)-3,5,8-trimethoxy-1,4-naphthoquinone ***(13c)****, (*[1]*H NMR - 500 MHz,* [13]*C NMR - 125 MHz, solvent - $CDCl_3$)*

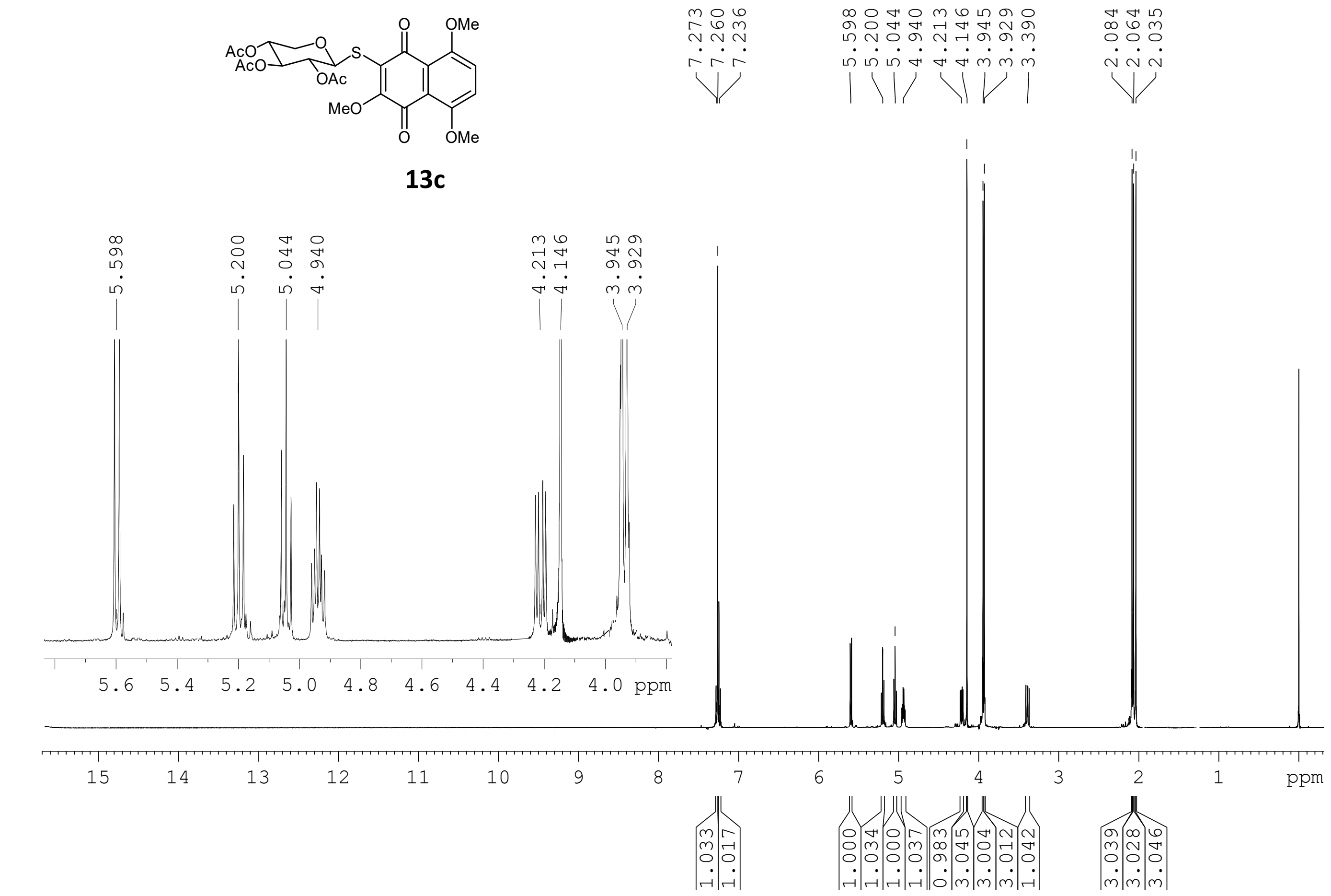

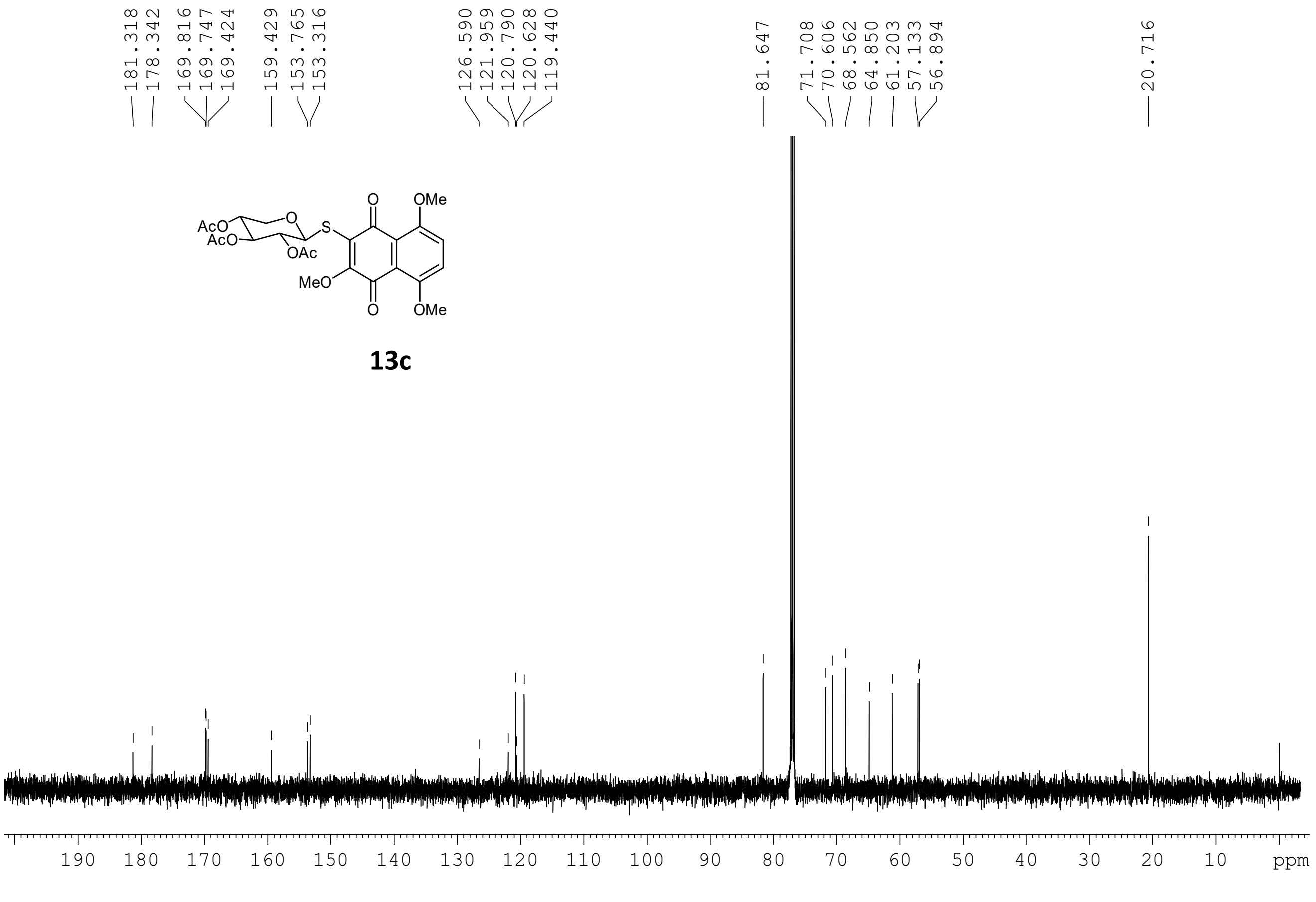

2-(2,3,4-Tri-O-acetyl-α-L-arabinopyranosyl-1-thio)-3,5,8-trimethoxy-1,4-naphthoquinone ***(13d)****, (^{1}H NMR - 500 MHz, ^{13}C NMR - 125 MHz, solvent - $CDCl_3$)*

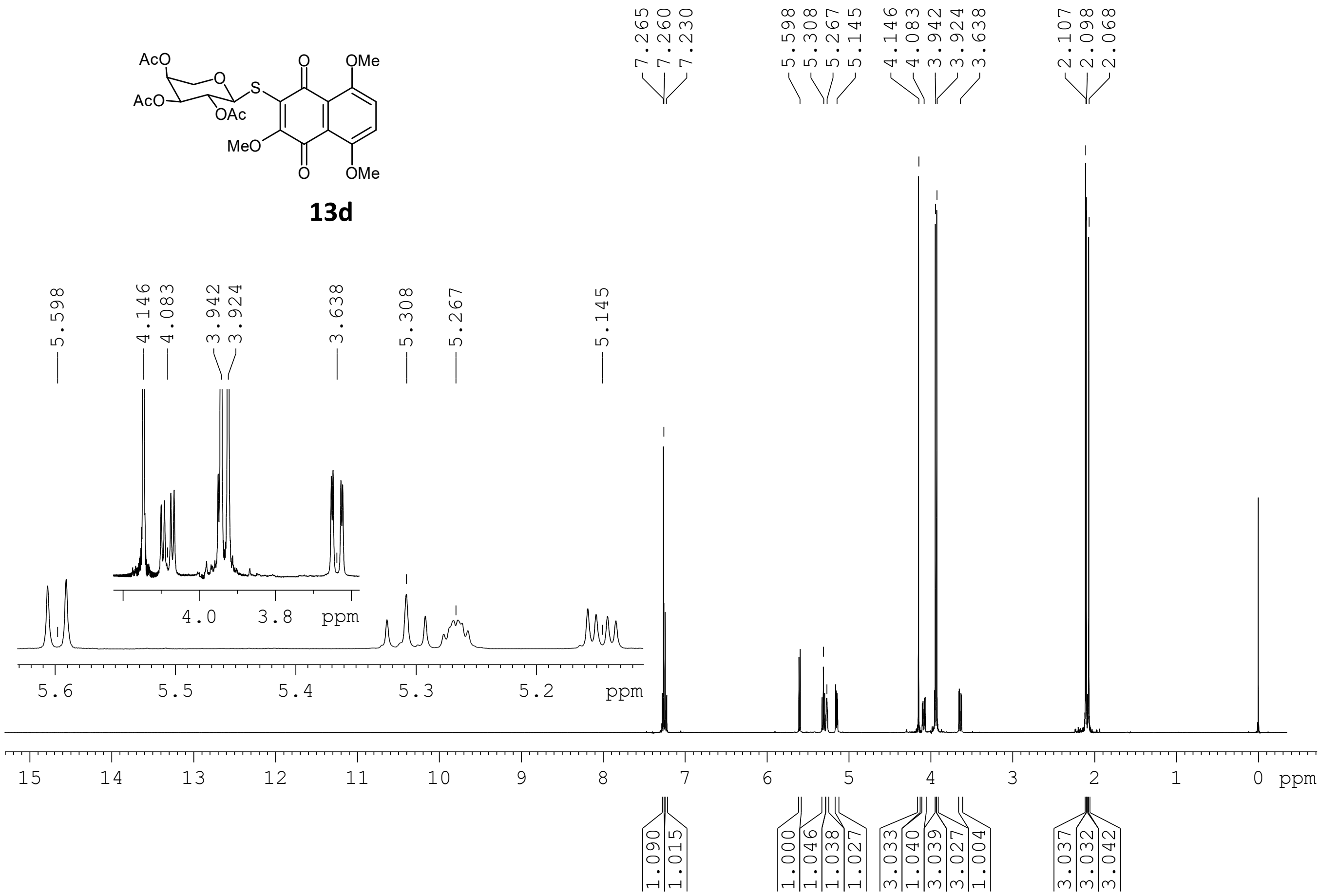

13d

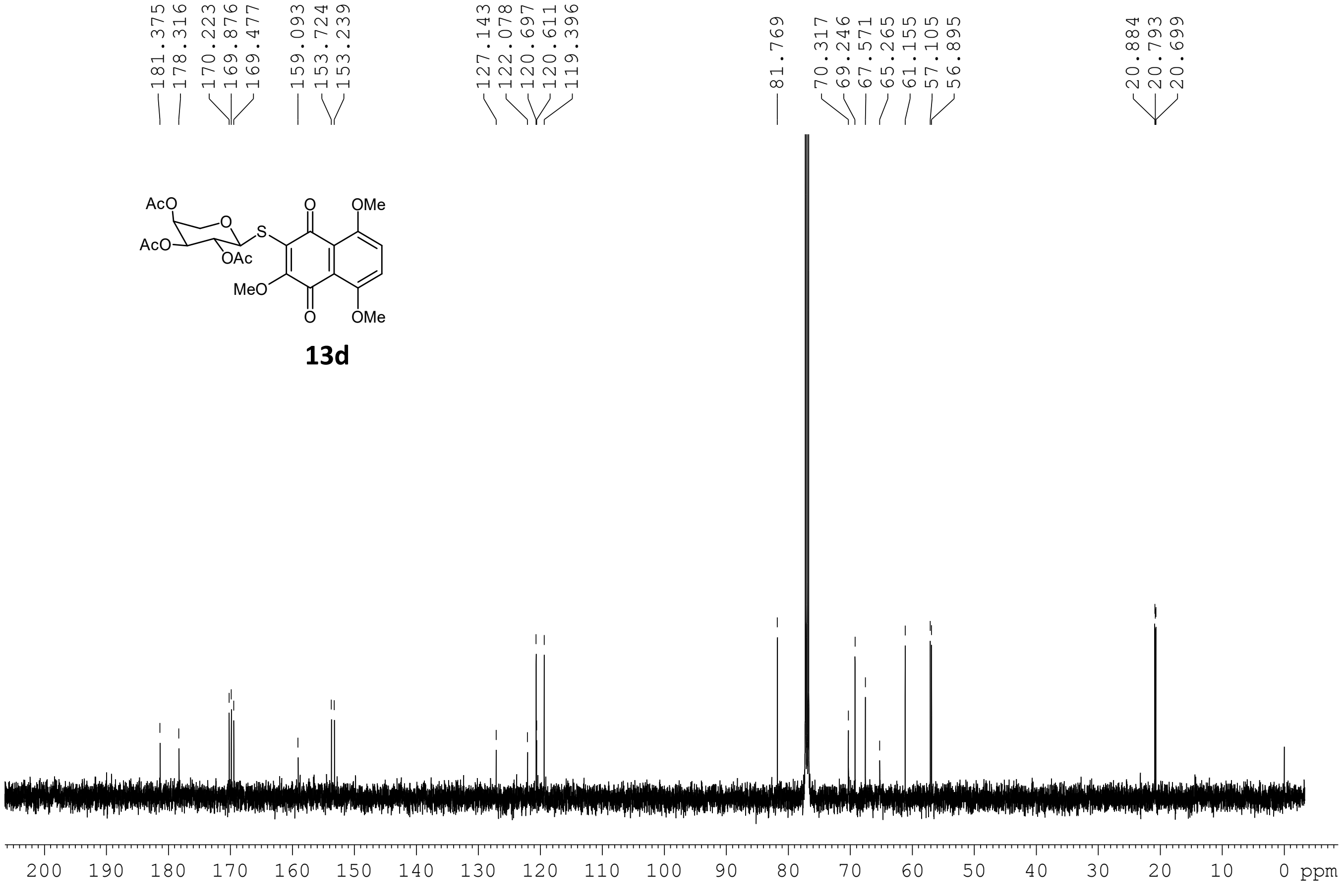

13d

2-(2,3,4,6-Tetra-O-acetyl-β-D-glucopyranosyl-1-thio)-6,7-dichloro-3,5,8-trimethoxy-1,4-naphthoquinone ***(14a)*** *,(^{1}H NMR - 500 MHz, ^{13}C NMR - 125 MHz, solvent - $CDCl_3$)*

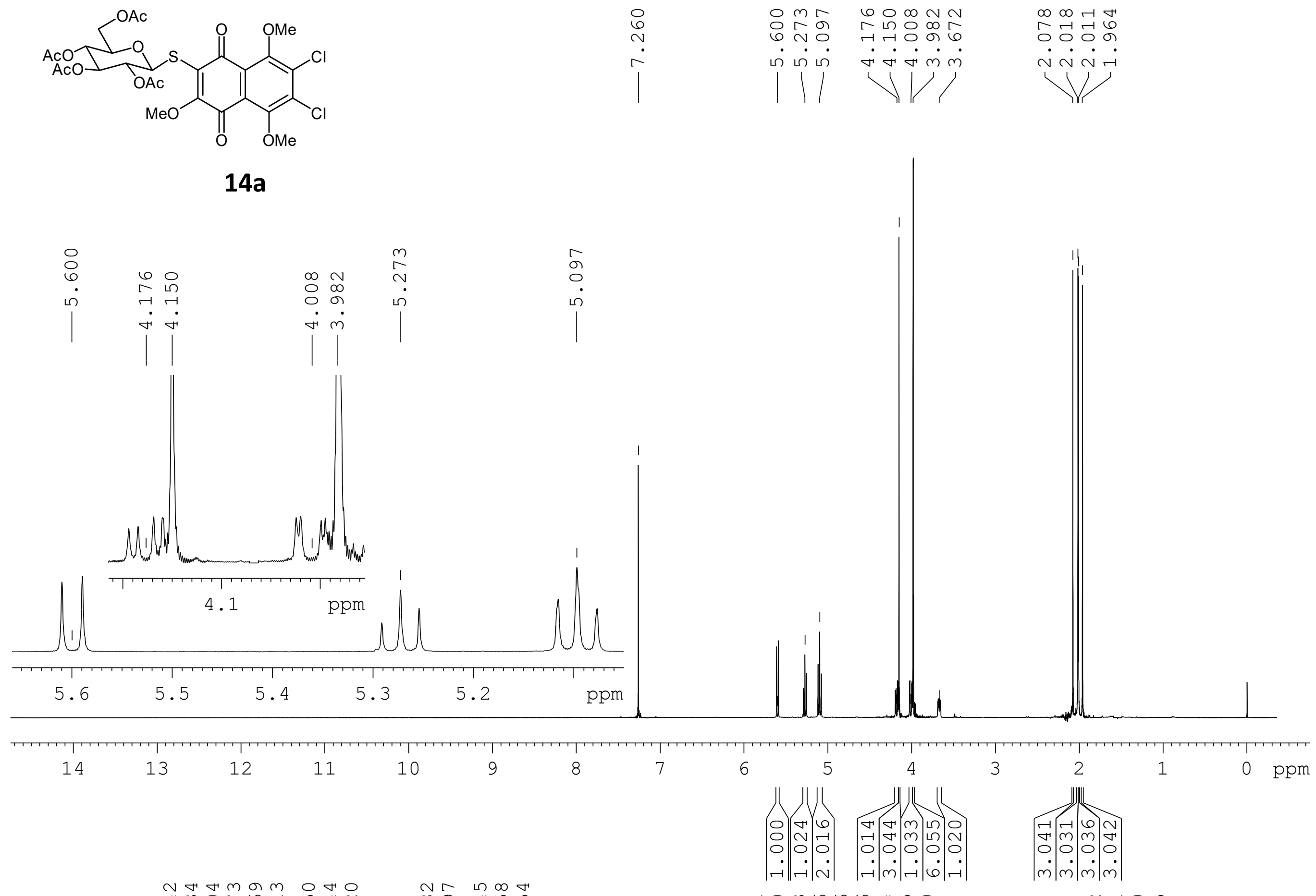

14a

14a

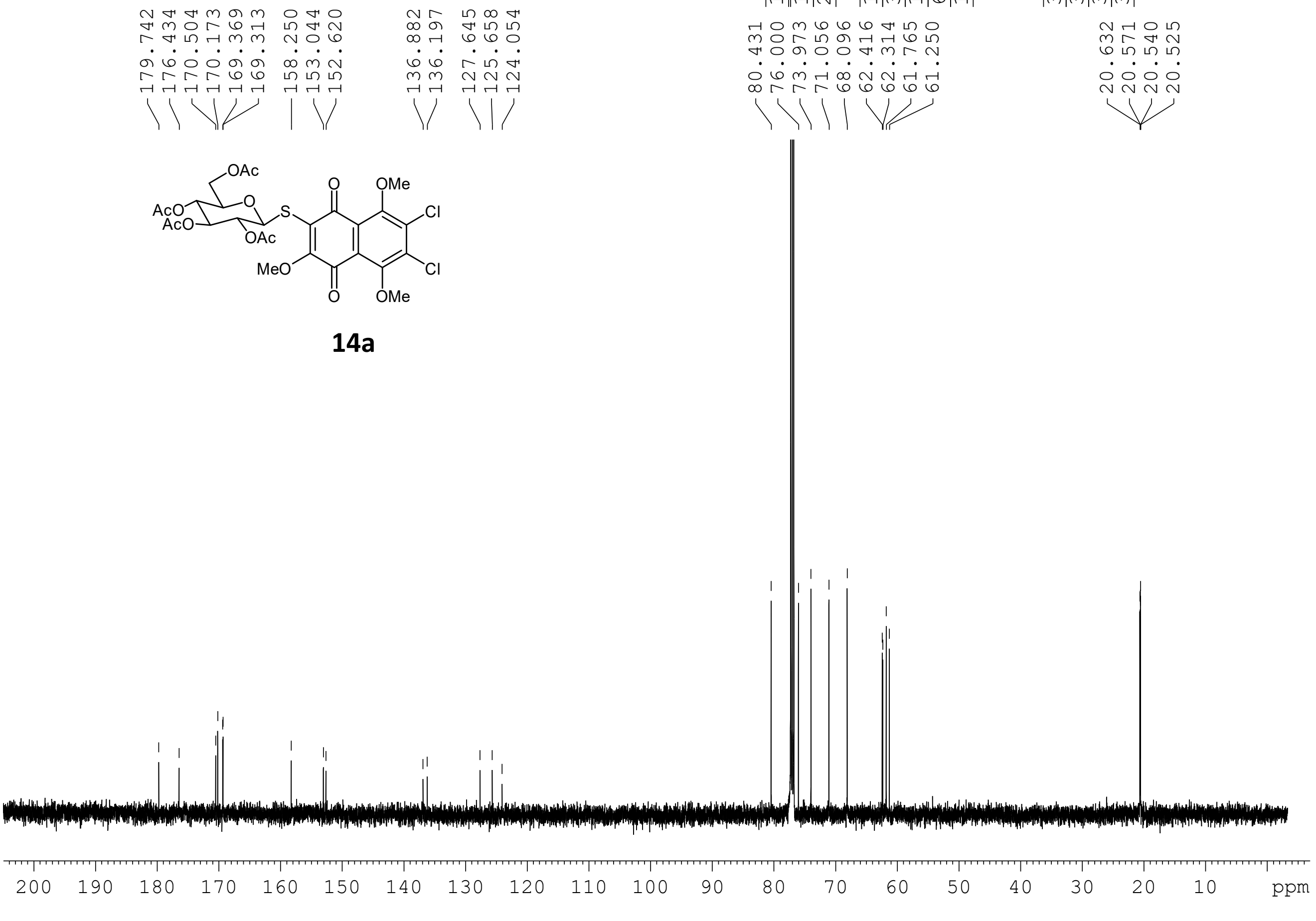

2-(2,3,4,6-Tetra-O-acetyl-β-D-galactopyranosyl-1-thio)-6,7-dichloro-3,5,8-trimethoxy-1,4-naphthoquinone ***(14b)****, (^{1}H NMR - 500 MHz, ^{13}C NMR - 125 MHz, solvent - CDCl*$_3$*)*

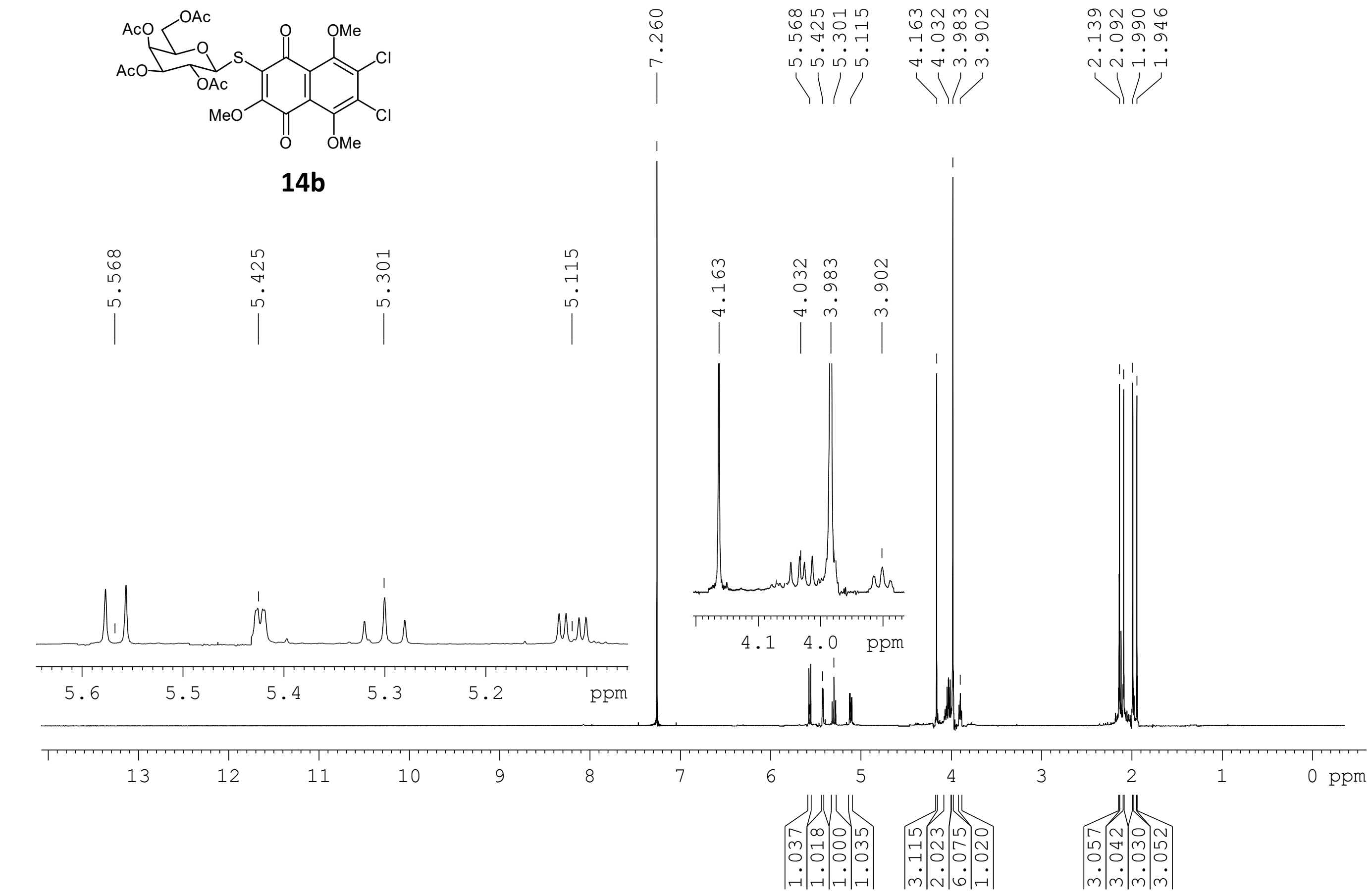

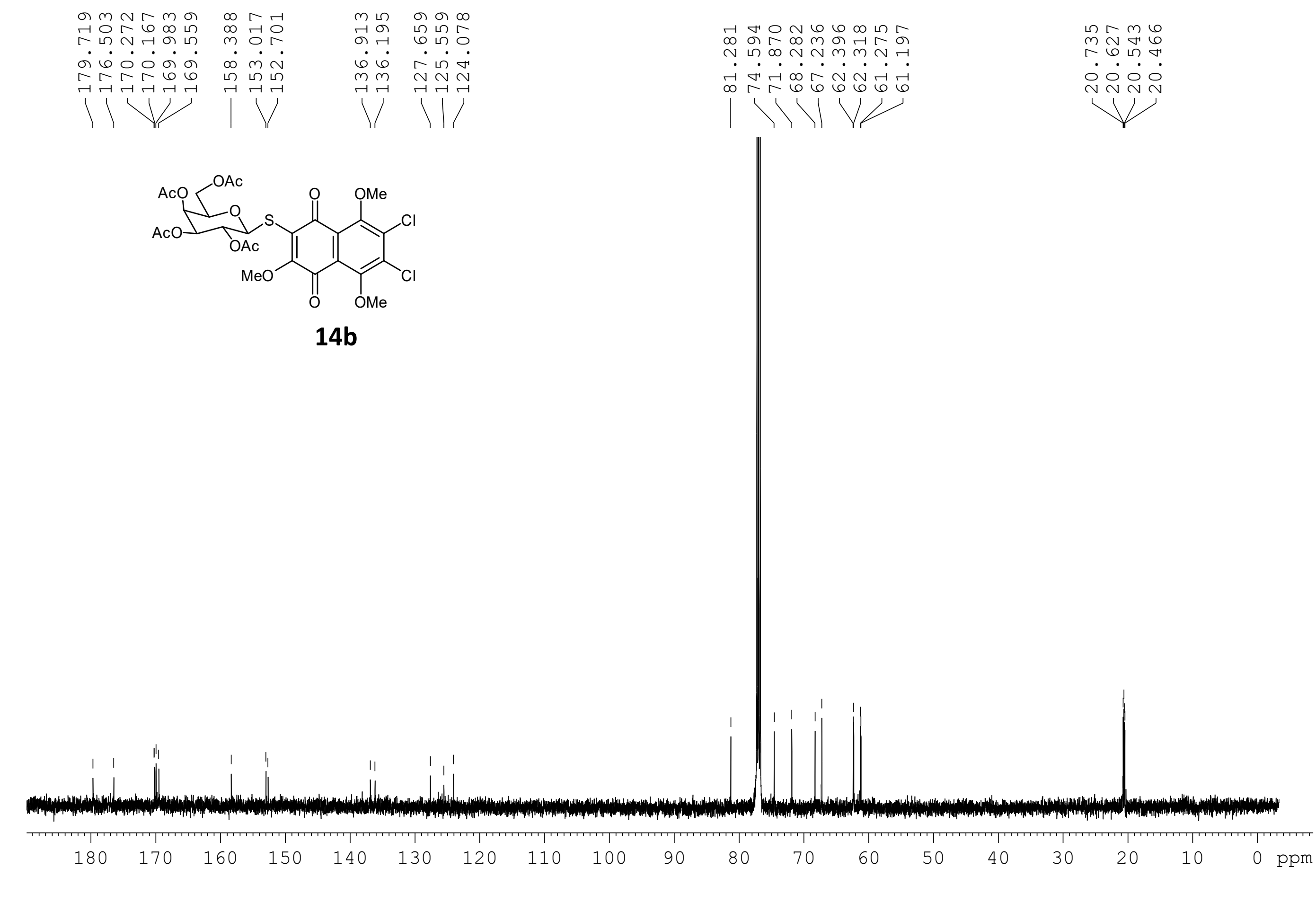

2-(2,3,4-Tri-O-acetyl-β-D-xylopyranosyl-1-thio)-6,7-dichloro-3,5,8-trimethoxy-1,4-naphthoquinone ***(14c)****, (^{1}H NMR - 500 MHz, ^{13}C NMR - 125 MHz, solvent - CDCl₃)*

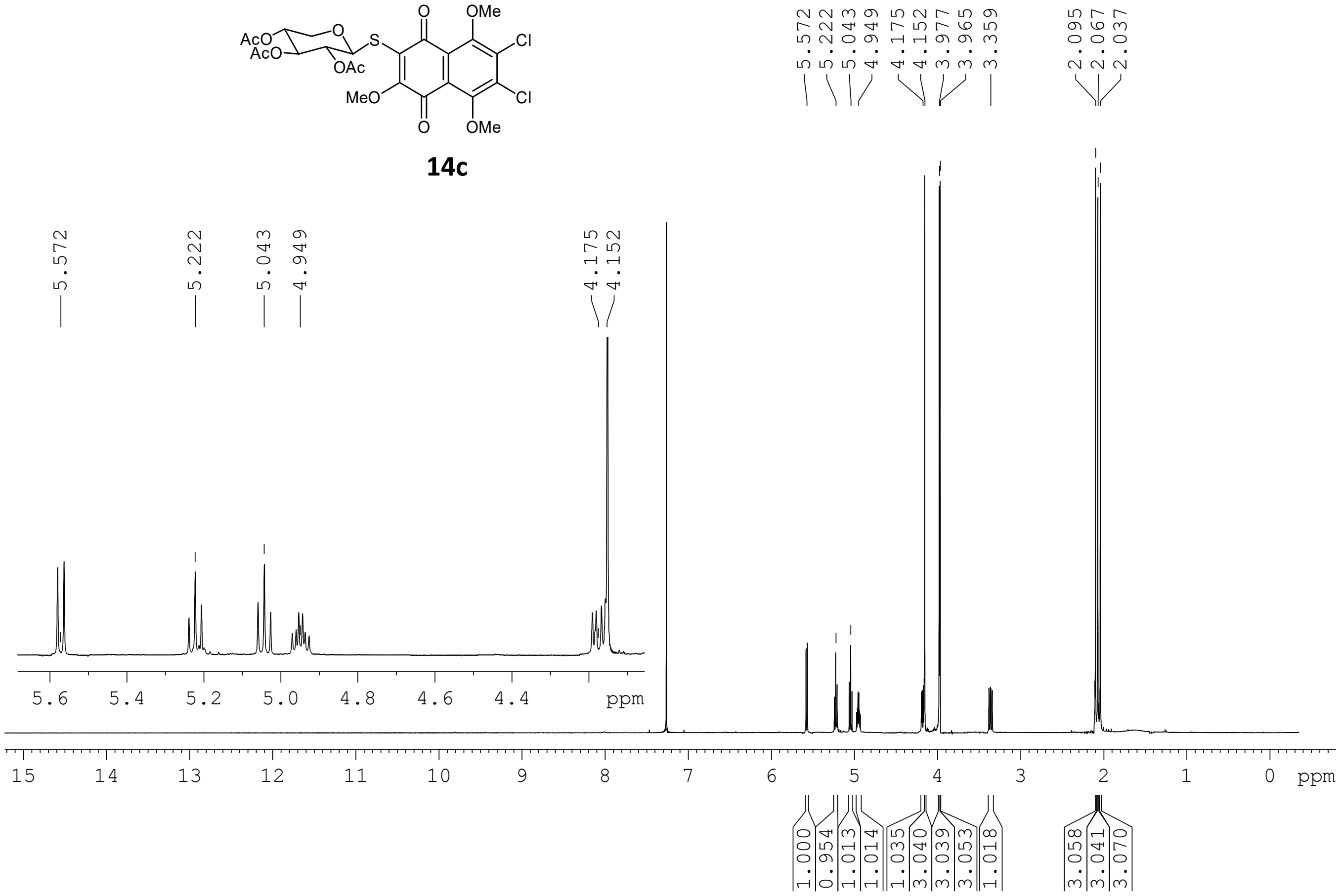

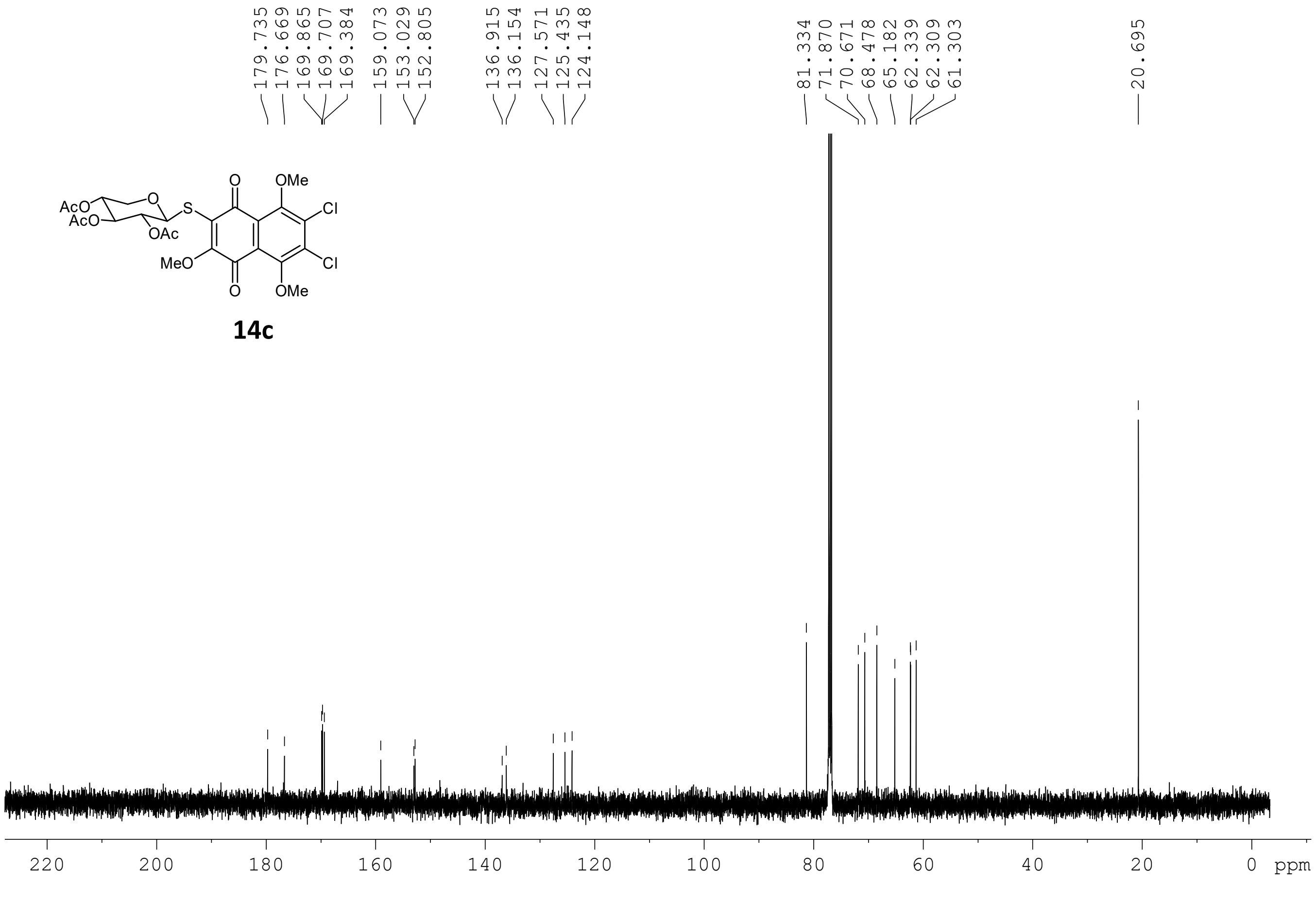

2-(2,3,4-Tri-O-acetyl-α-L-arabinopyranosyl-1-thio)-6,7-dichloro-3,5,8-trimethoxy-1,4-naphthoquinone ***(14d)****, (^{1}H NMR - 500 MHz, ^{13}C NMR - 125 MHz, solvent - $CDCl_3$)*

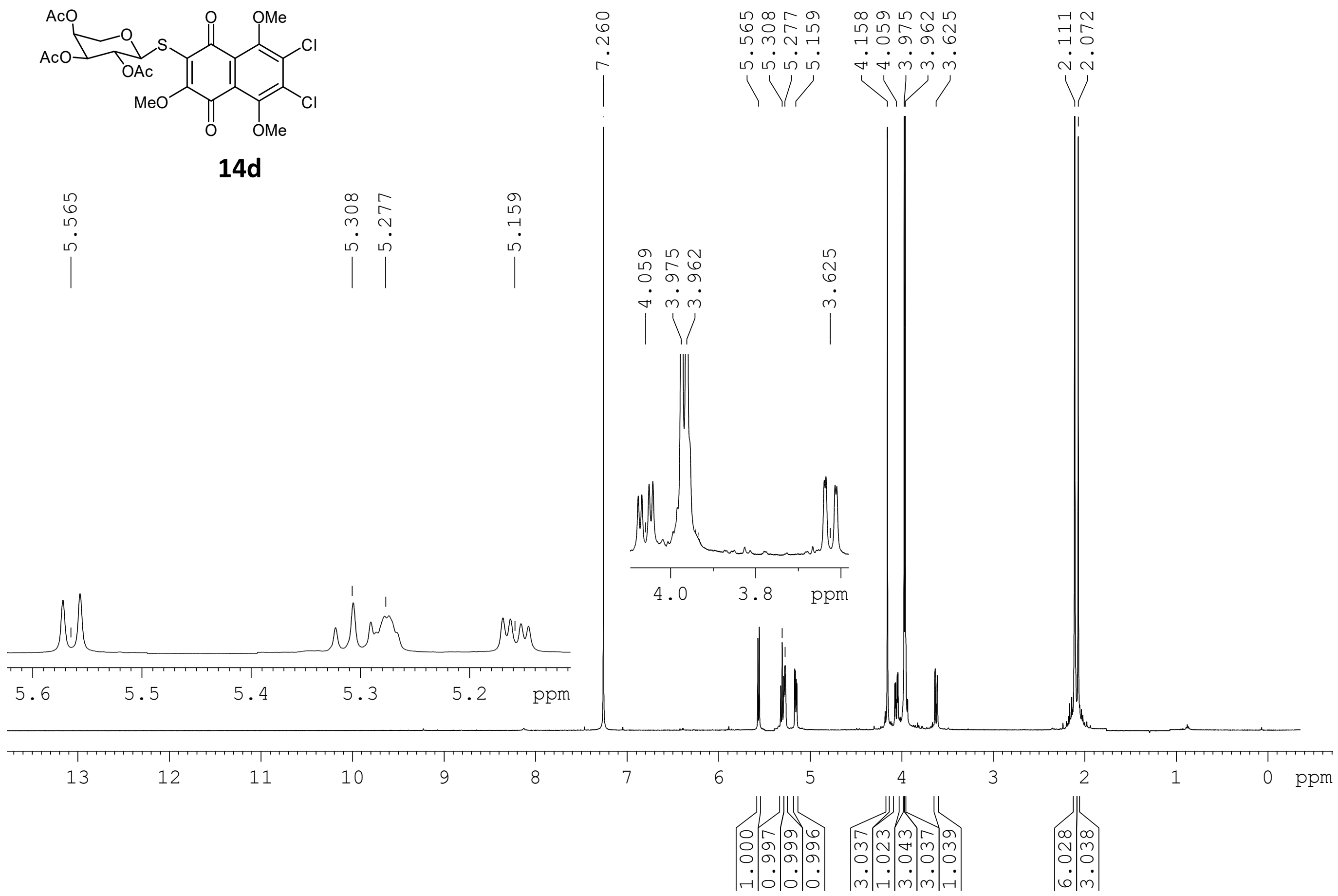

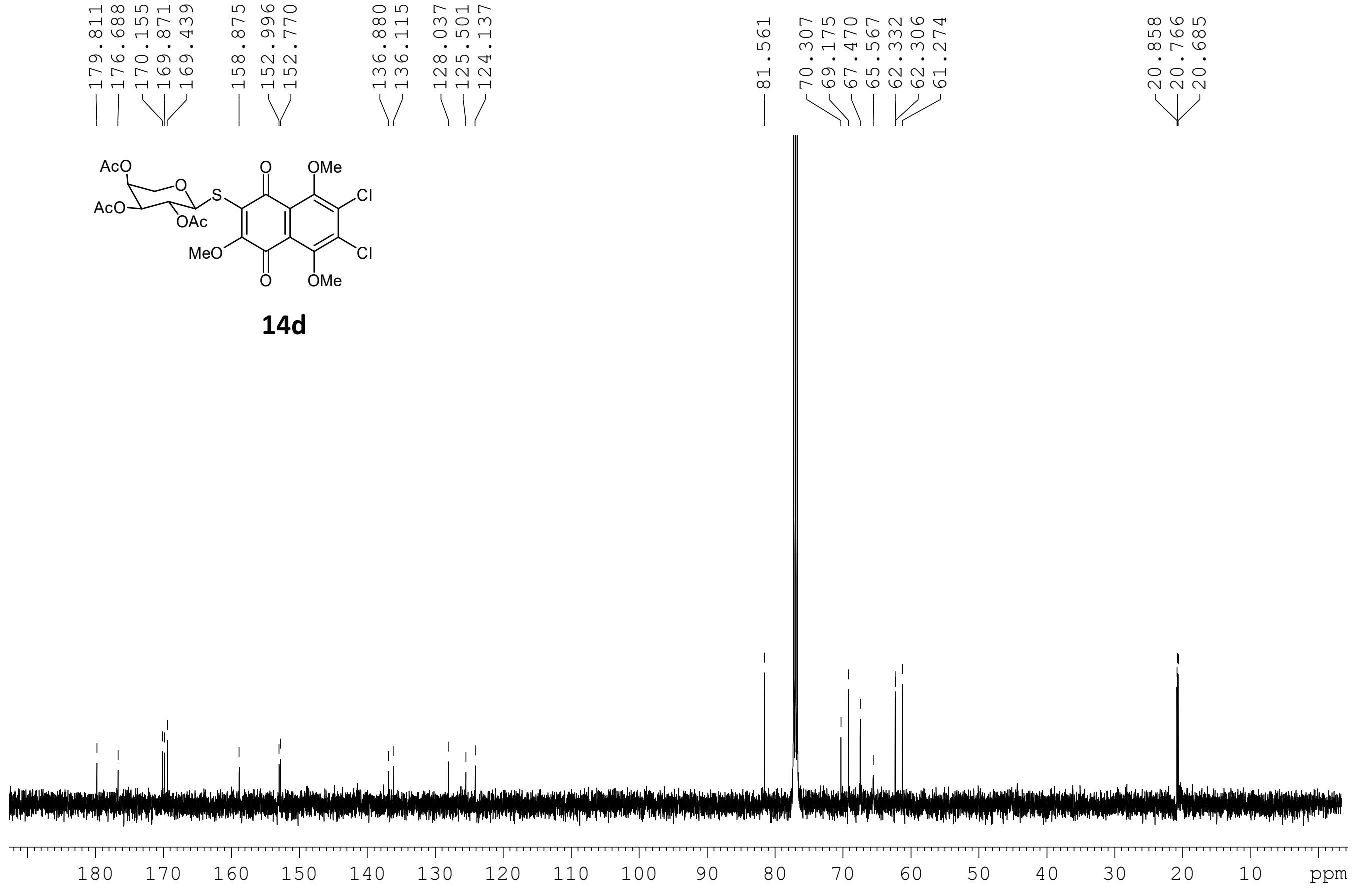

2-(2,3,4,6-Tetra-O-acetyl-β-D-glucopyranosyl-1-thio)-8-hydroxy-3-methoxy-1,4-naphthoquinone ***(15a)***, *(^{1}H NMR - 700 MHz, ^{13}C NMR - 176 MHz, solvent - $CDCl_3$)*

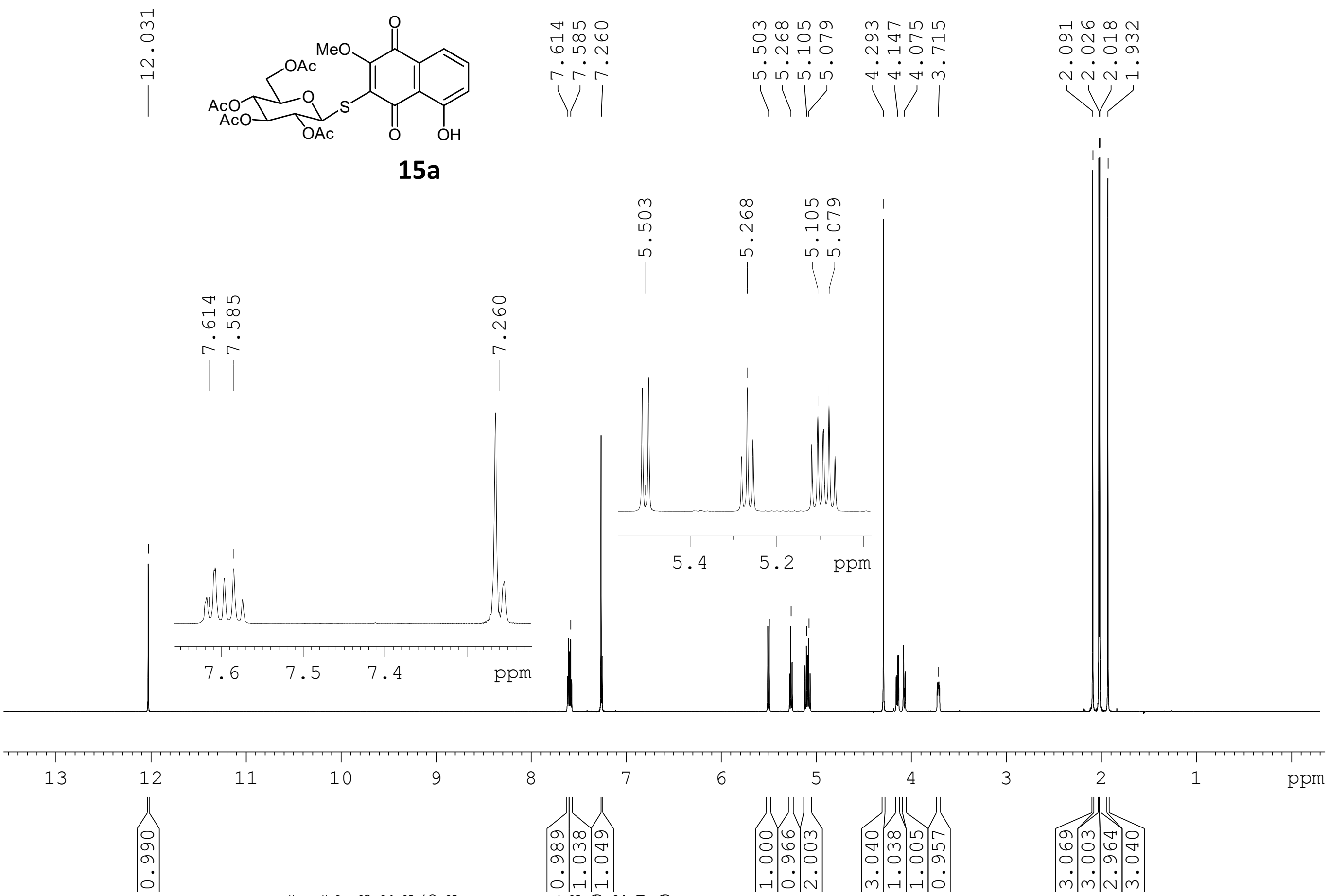

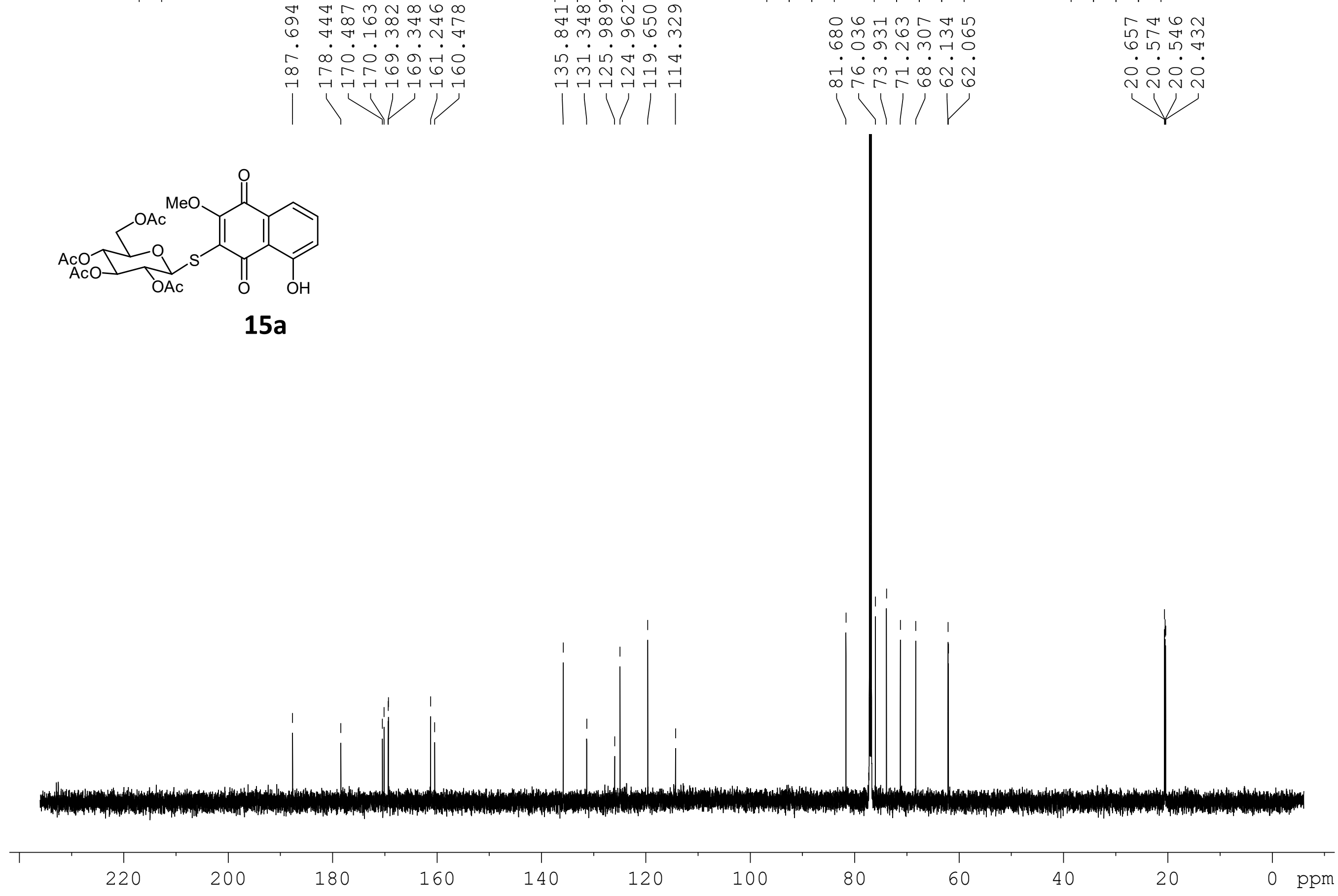

2-(2,3,4,6-Tetra-O-acetyl-β-D-galactopyranosyl-1-thio)-8-hydroxy-3-methoxy-1,4-naphthoquinone ***(15b)***, *(^{1}H NMR - 500 MHz, ^{13}C NMR - 125 MHz, solvent - CDCl₃)*

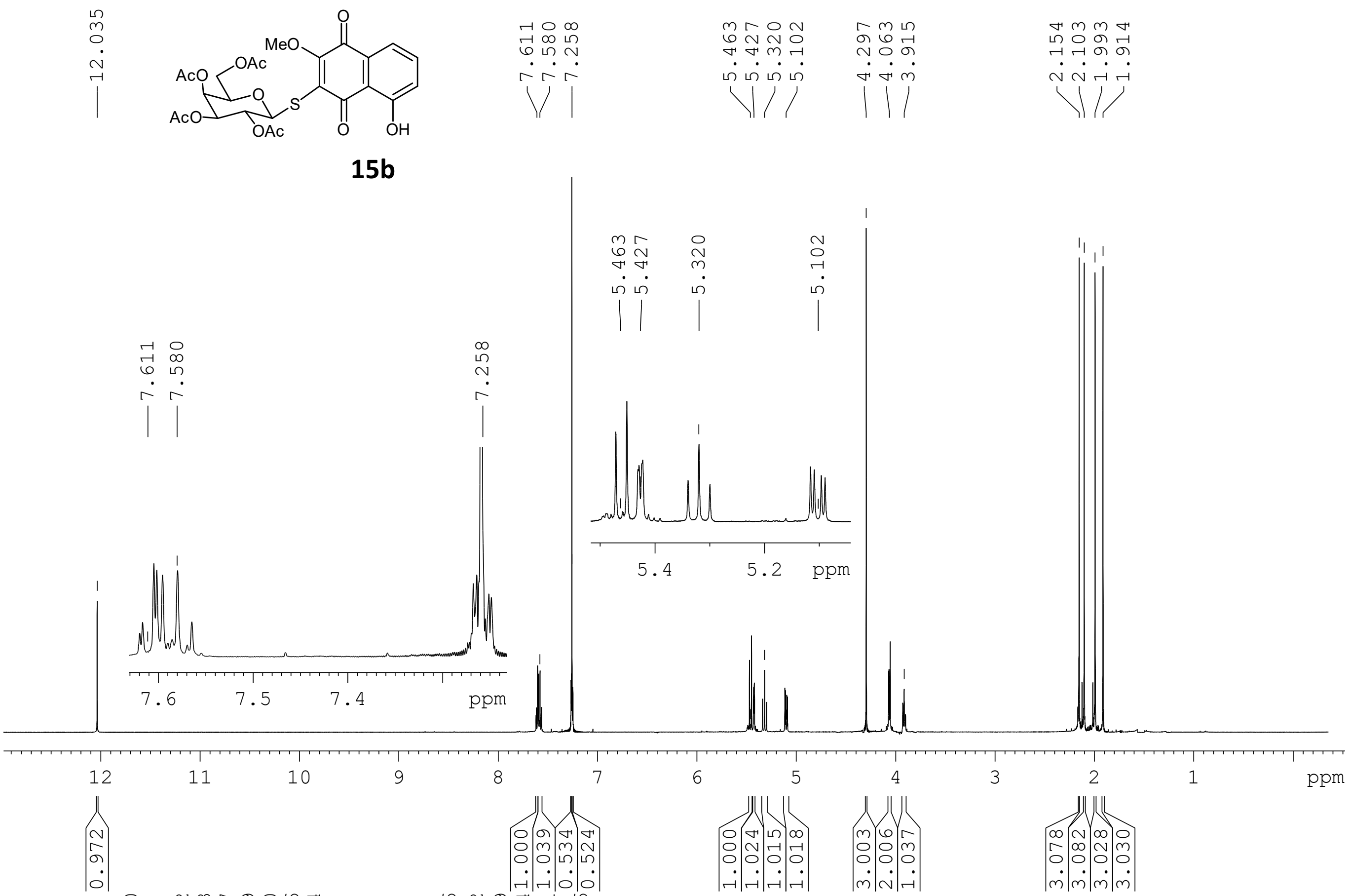

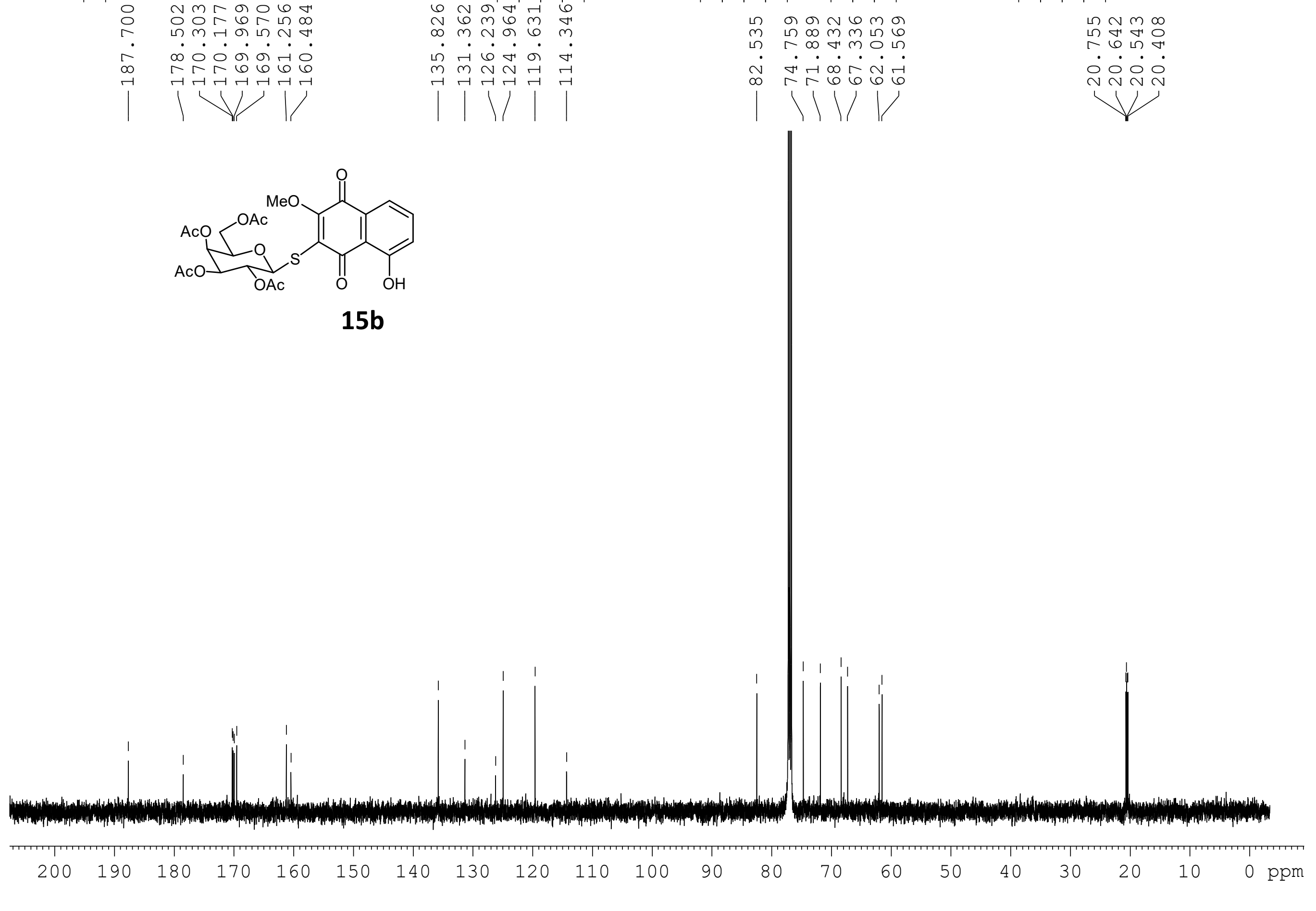

2-(2,3,4-Tri-O-acetyl-β-D-xylopyranosyl-1-thio)-8-hydroxy-3-methoxy-1,4-naphthoquinone ***(15c)****, (*^{1}*H NMR - 500 MHz,* ^{13}*C NMR - 125 MHz, solvent - CDCl*$_3$*)*

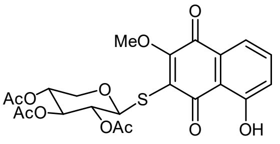

15c

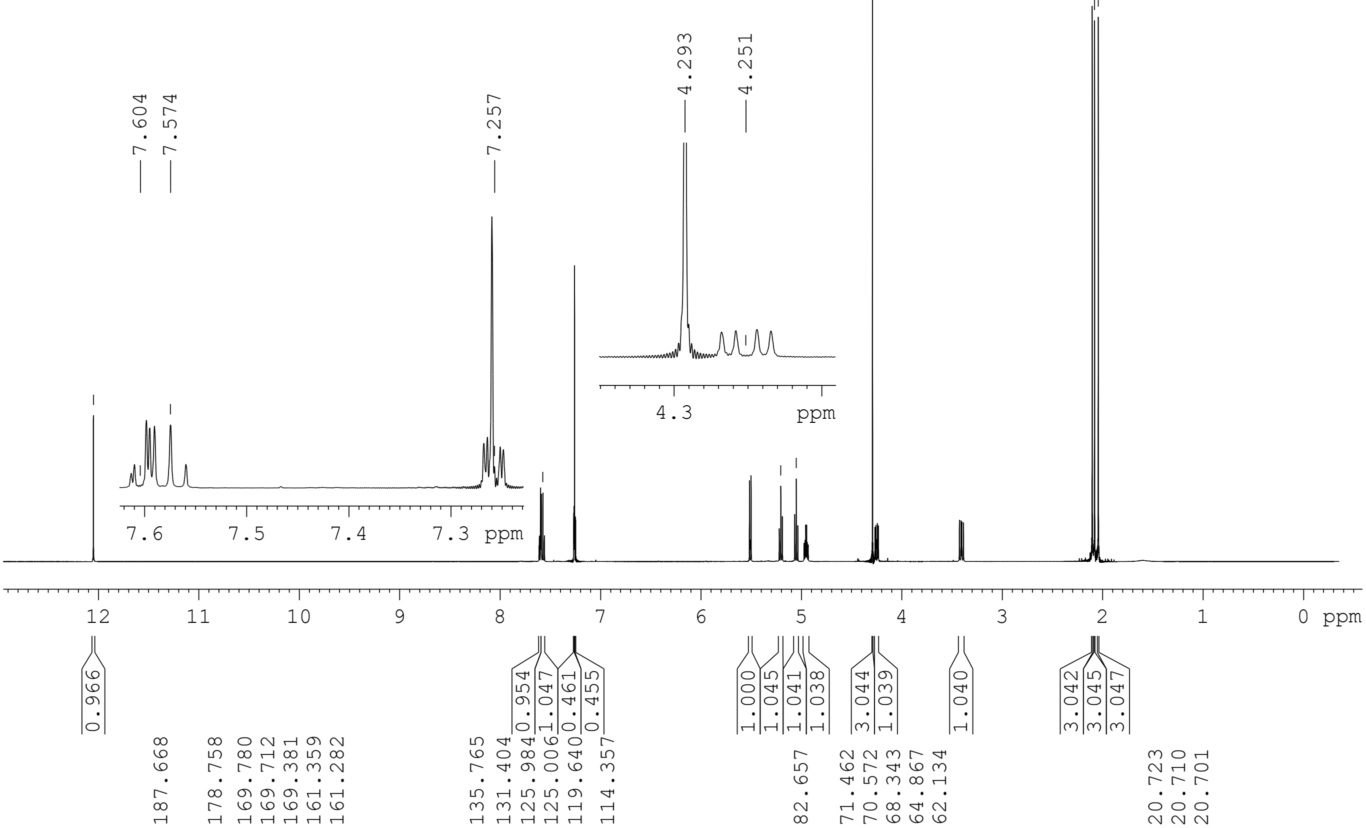

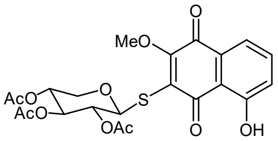

15c

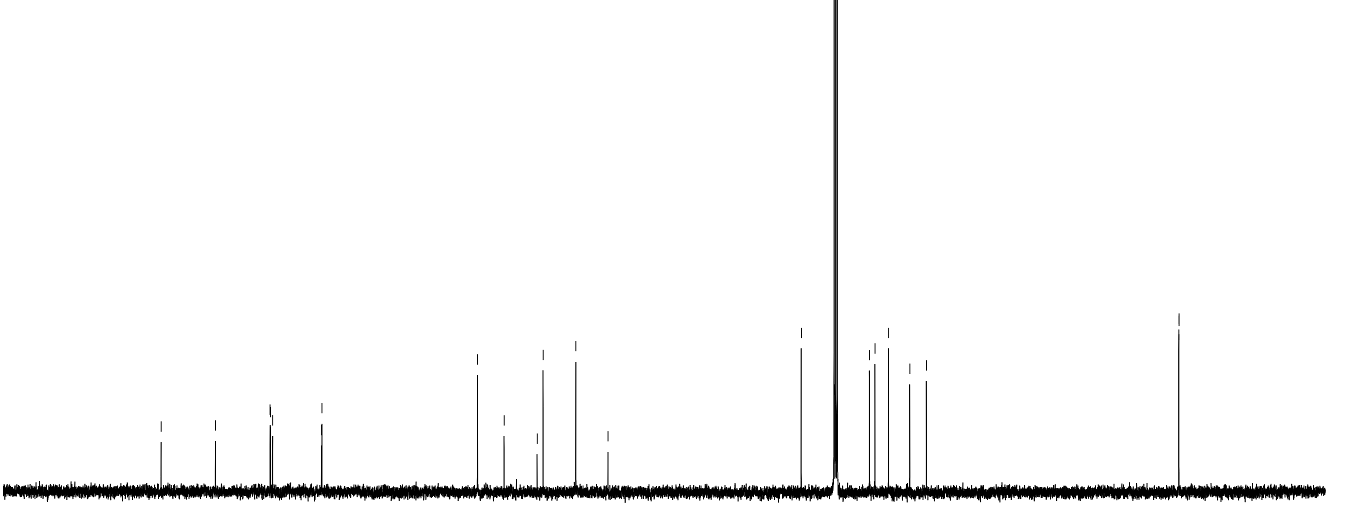

2-(2,3,4-Tri-O-acetyl-α-L-arabinopyranosyl-1-thio)-8-hydroxy-3-methoxy-1,4-naphthoquinone ***(15d)***, *(^{1}H NMR - 500 MHz, ^{13}C NMR - 125 MHz, solvent - $CDCl_3$)*

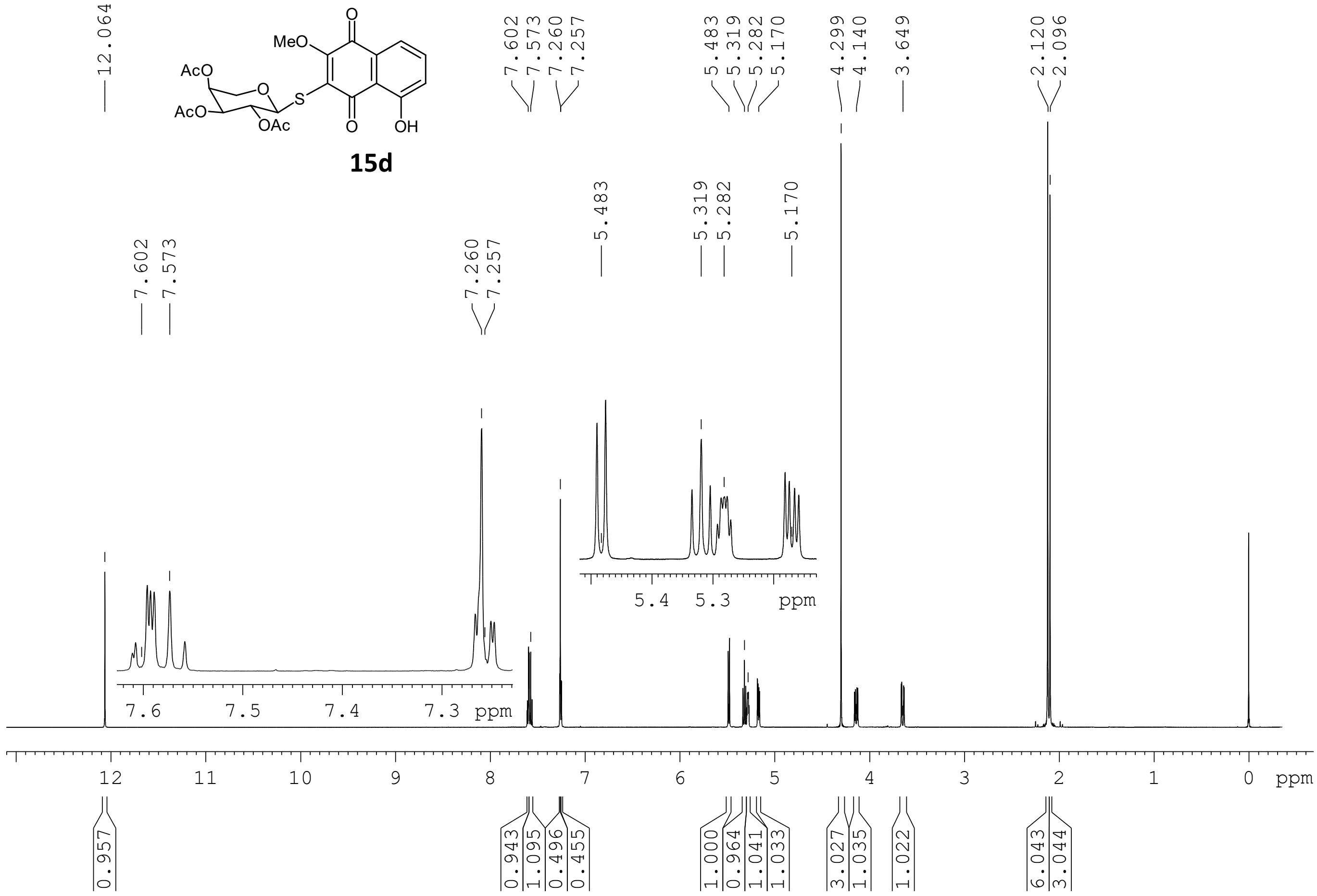

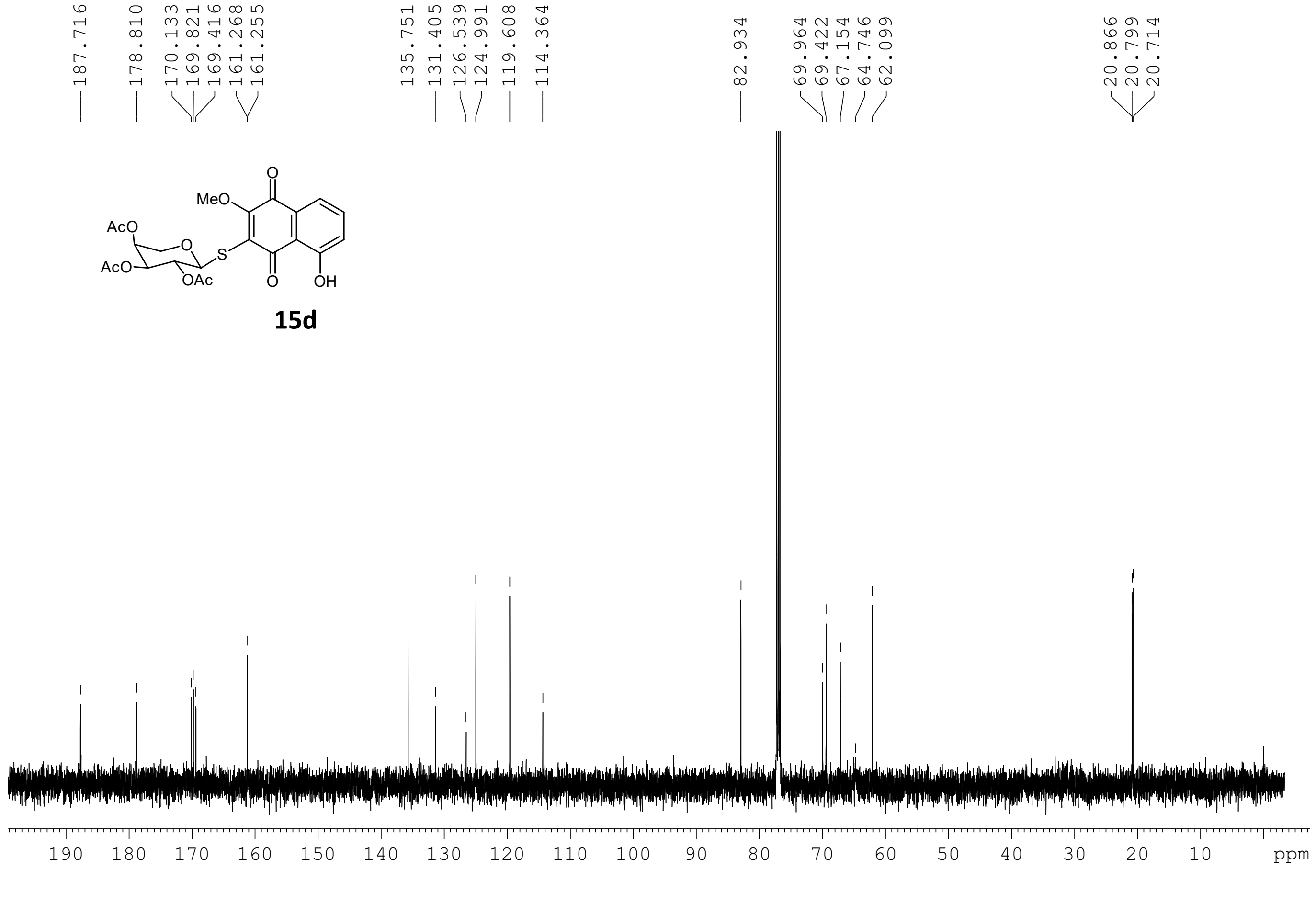

2-(2,3,4,6-Tetra-O-acetyl-β-D-glucopyranosyl-1-thio)-5-hydroxy-3-methoxy-1,4-naphthoquinone ***(16a)****, (^{1}H NMR - 500 MHz, ^{13}C NMR - 125 MHz, solvent - CDCl₃)*

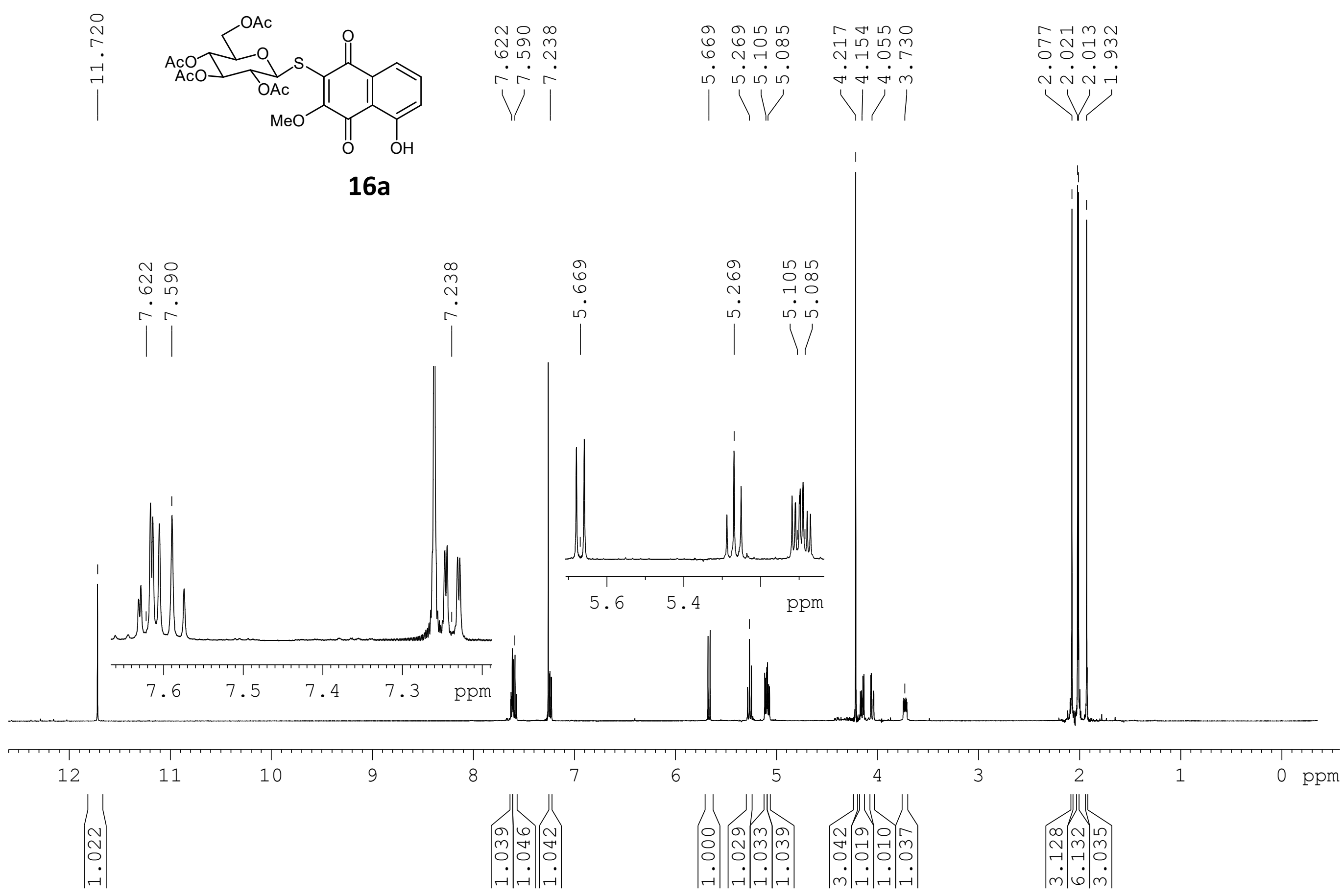

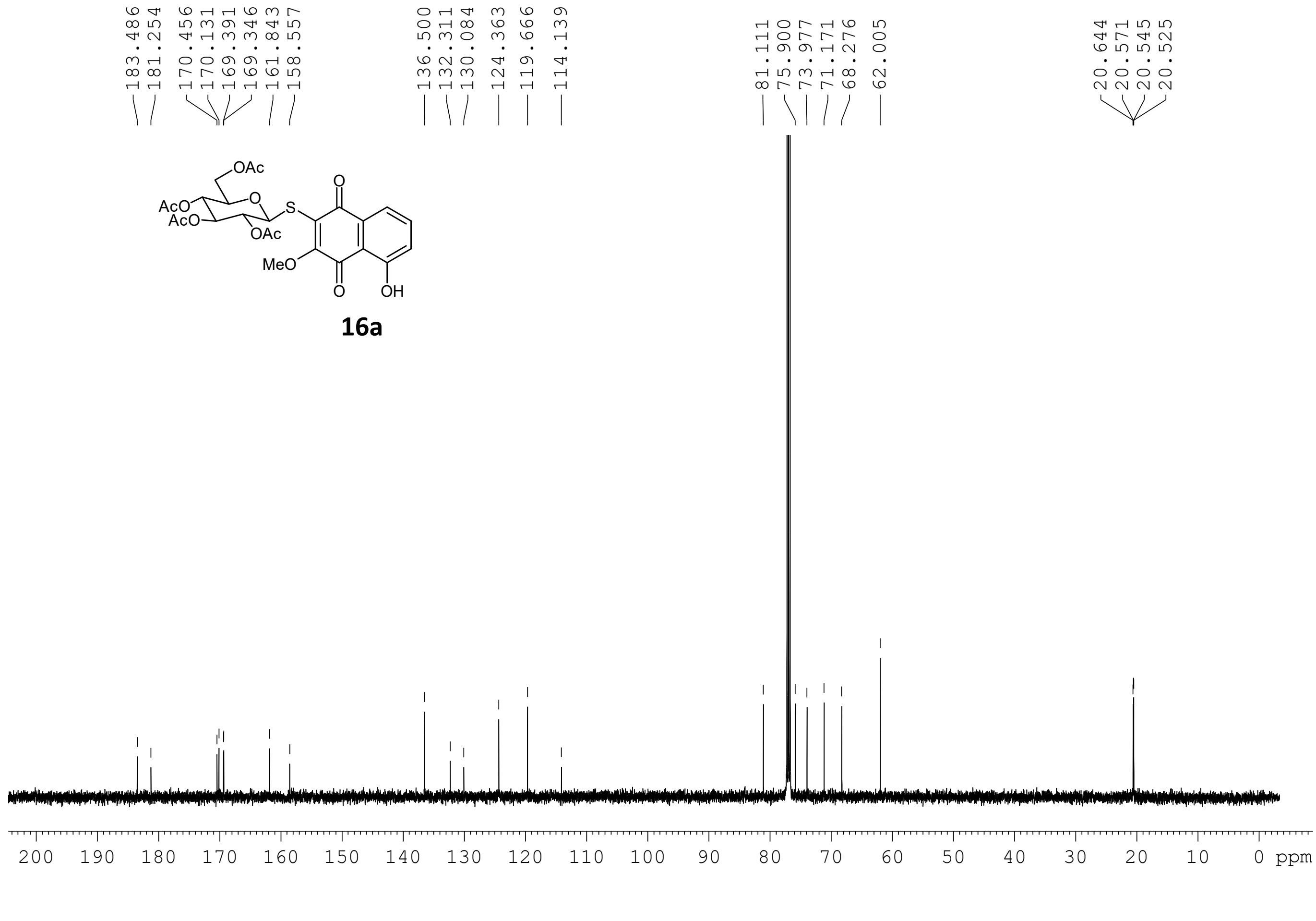

2-(2,3,4,6-Tetra-O-acetyl-β-D-galactopyranosyl-1-thio)-5-hydroxy-3-methoxy-1,4-naphthoquinone ***(16b)****, (^{1}H NMR - 500 MHz, ^{13}C NMR - 125 MHz, solvent - $CDCl_3$)*

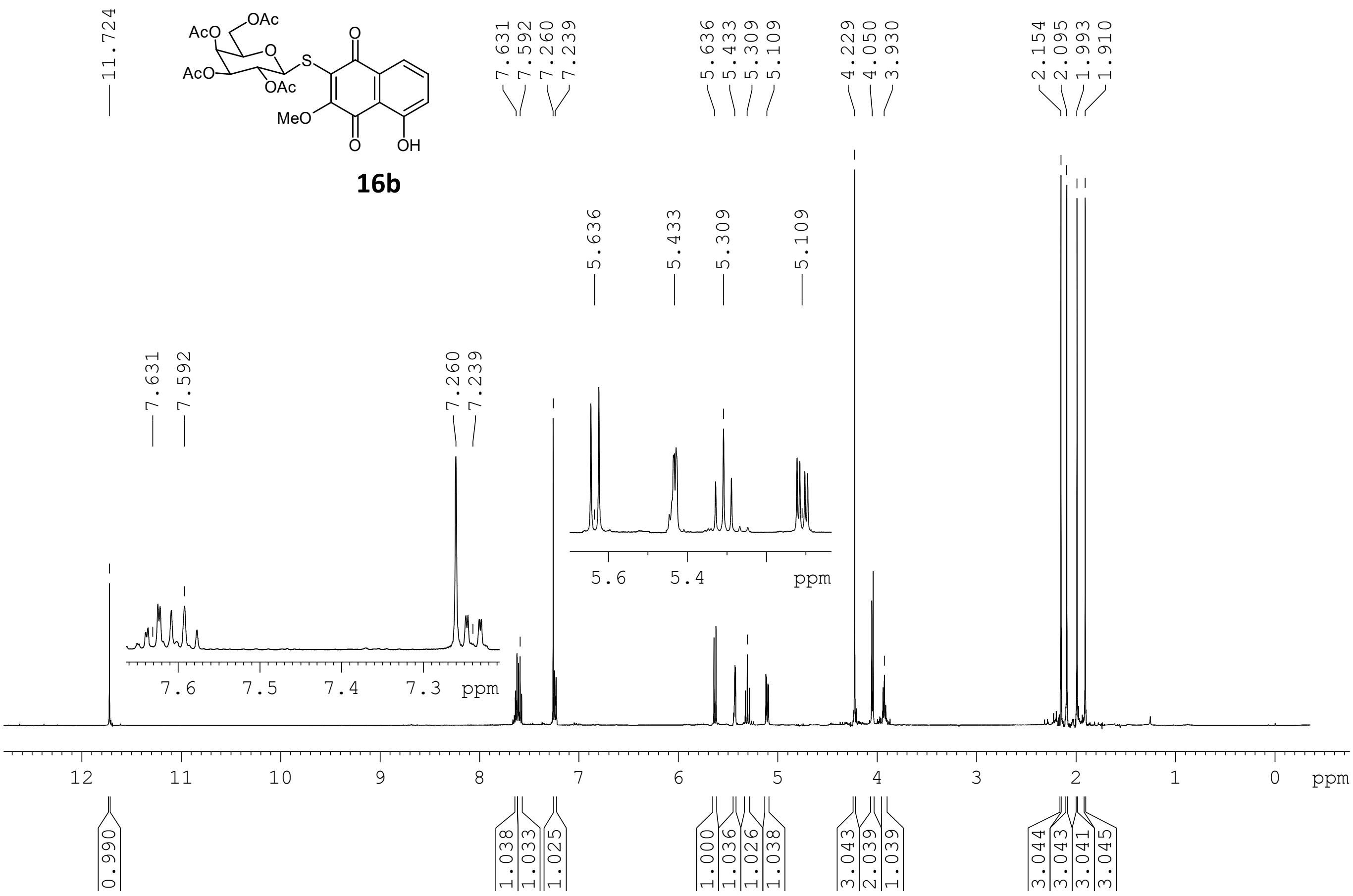

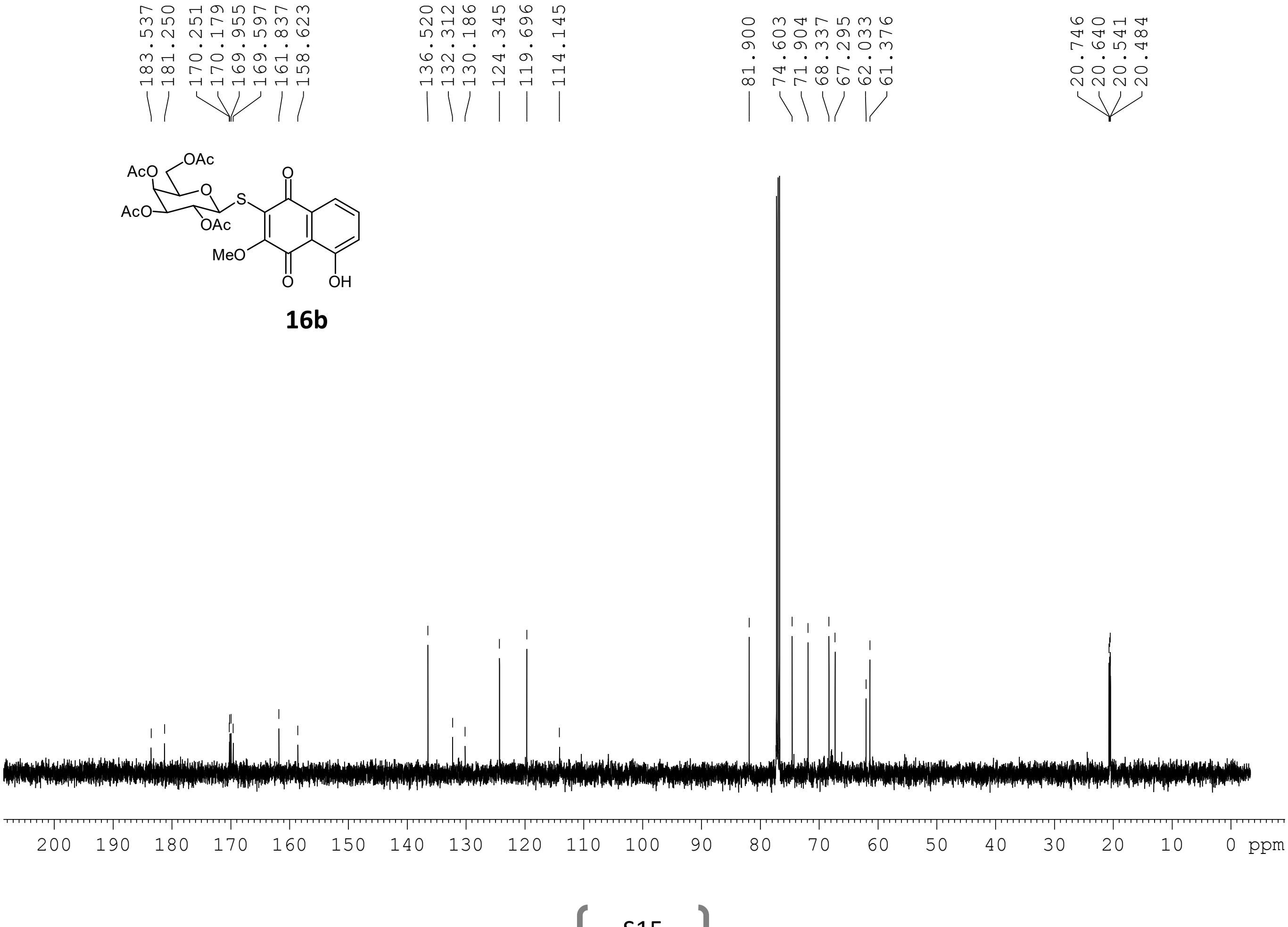

2-(2,3,4-Tri-O-acetyl-β-D-xylopyranosyl-1-thio)-5-hydroxy-3-methoxy-1,4-naphthoquinone ***(16c)****, (^{1}H NMR - 500 MHz, ^{13}C NMR - 125 MHz, solvent - $CDCl_3$)*

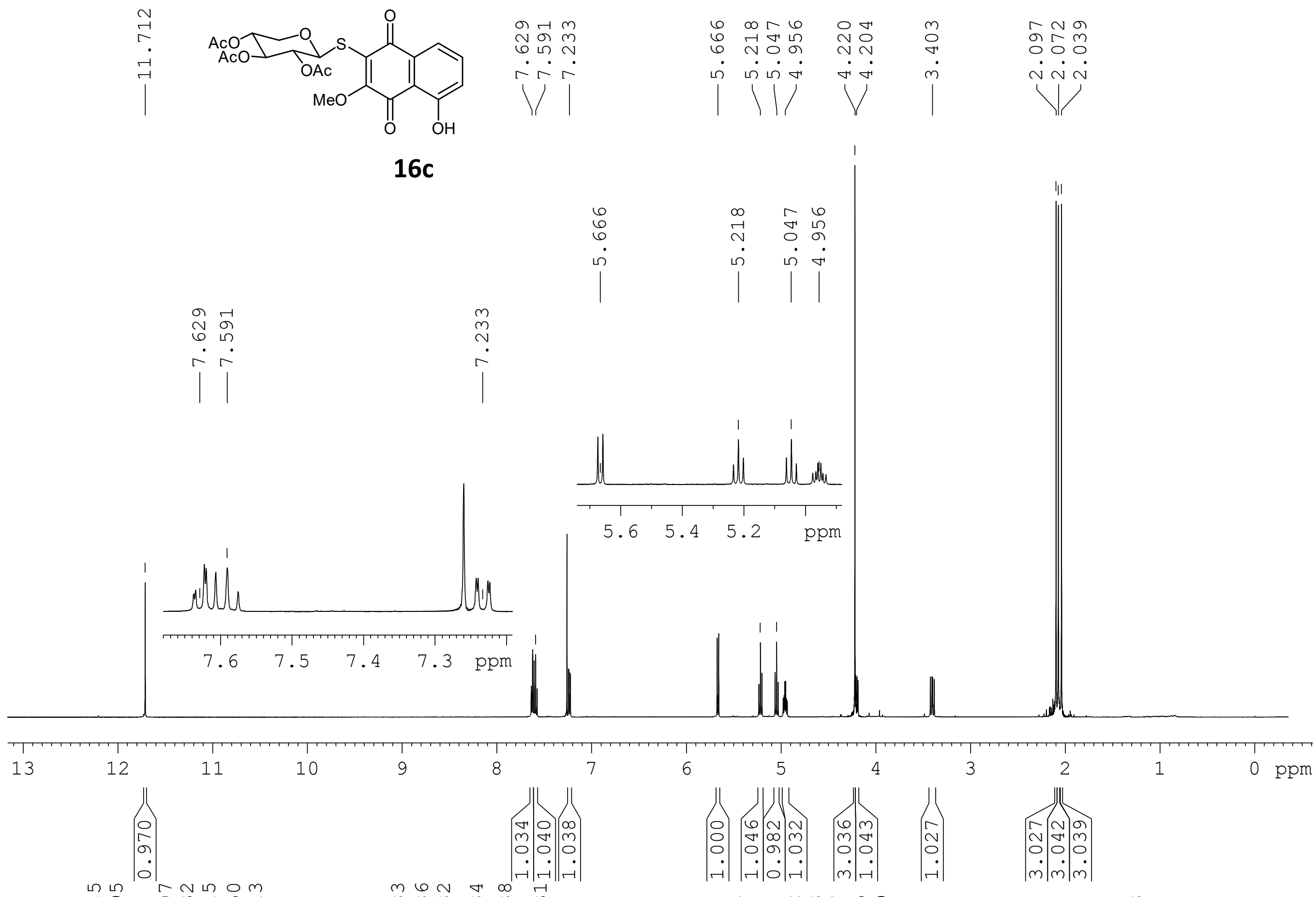

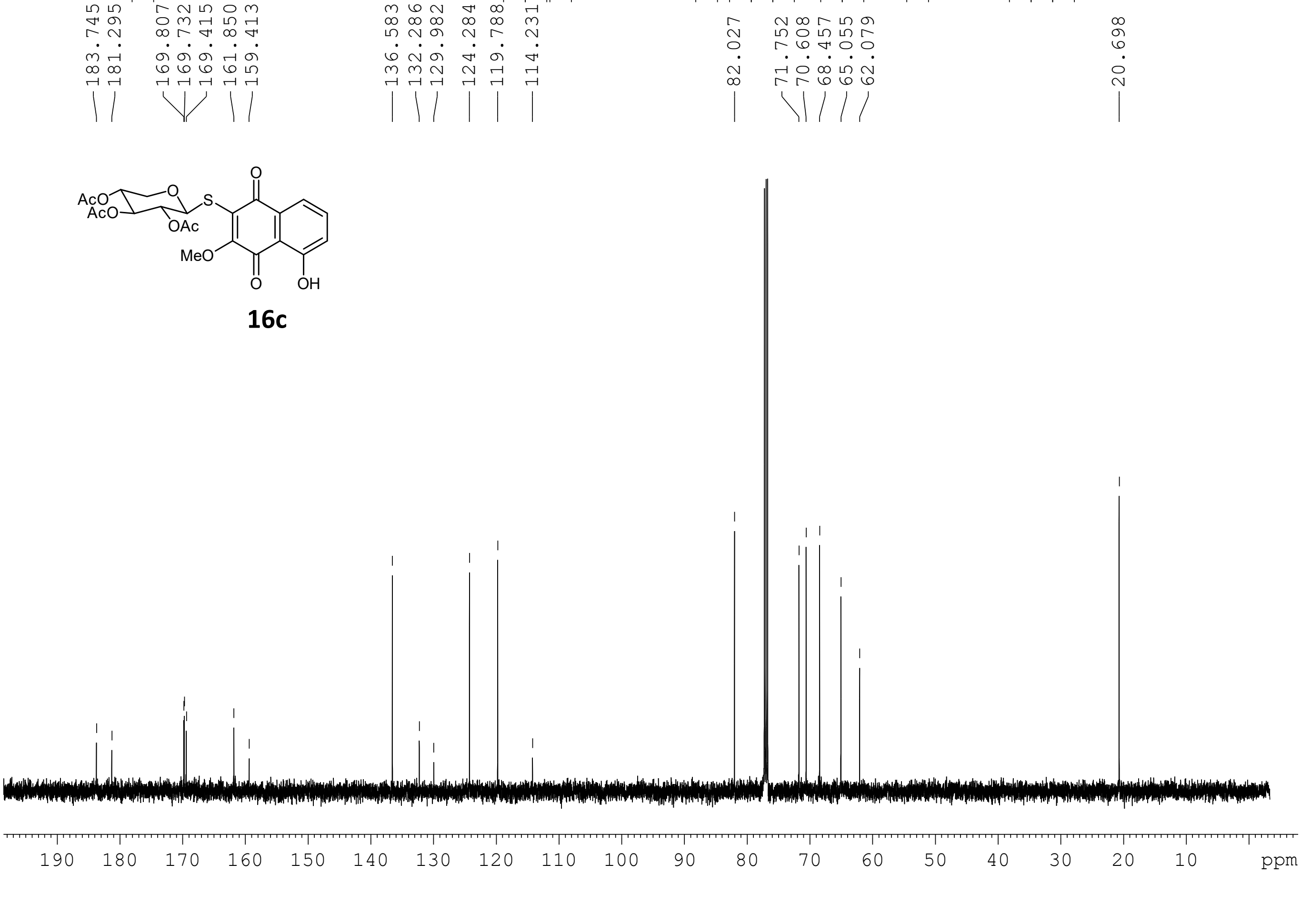

2-(2,3,4-Tri-O-acetyl-α-L-arabinopyranosyl-1-thio)-5-hydroxy-3-methoxy-1,4-naphthoquinone ***(16d)***, *(^{1}H NMR - 700 MHz, ^{13}C NMR - 176 MHz, solvent - $CDCl_3$)*

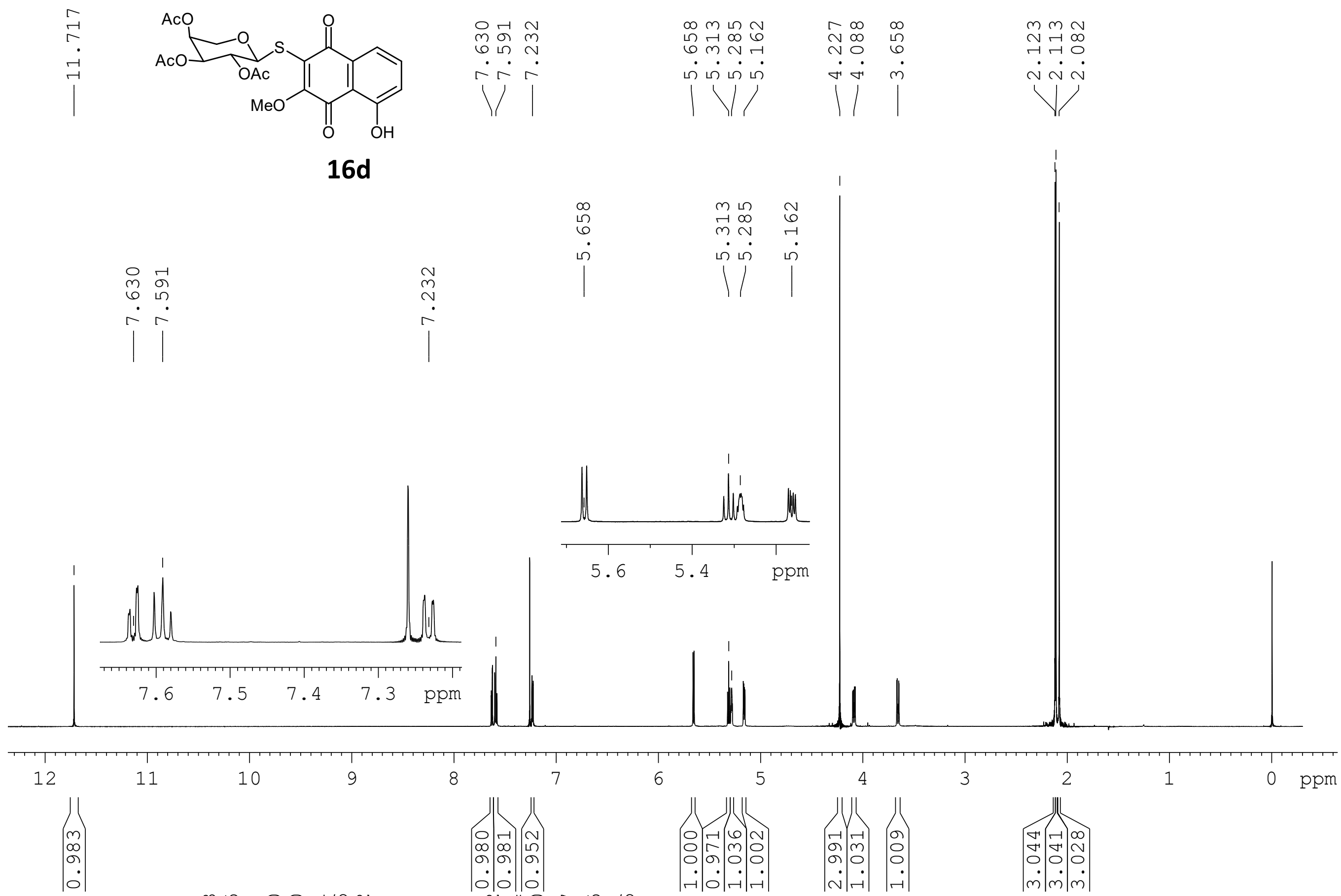

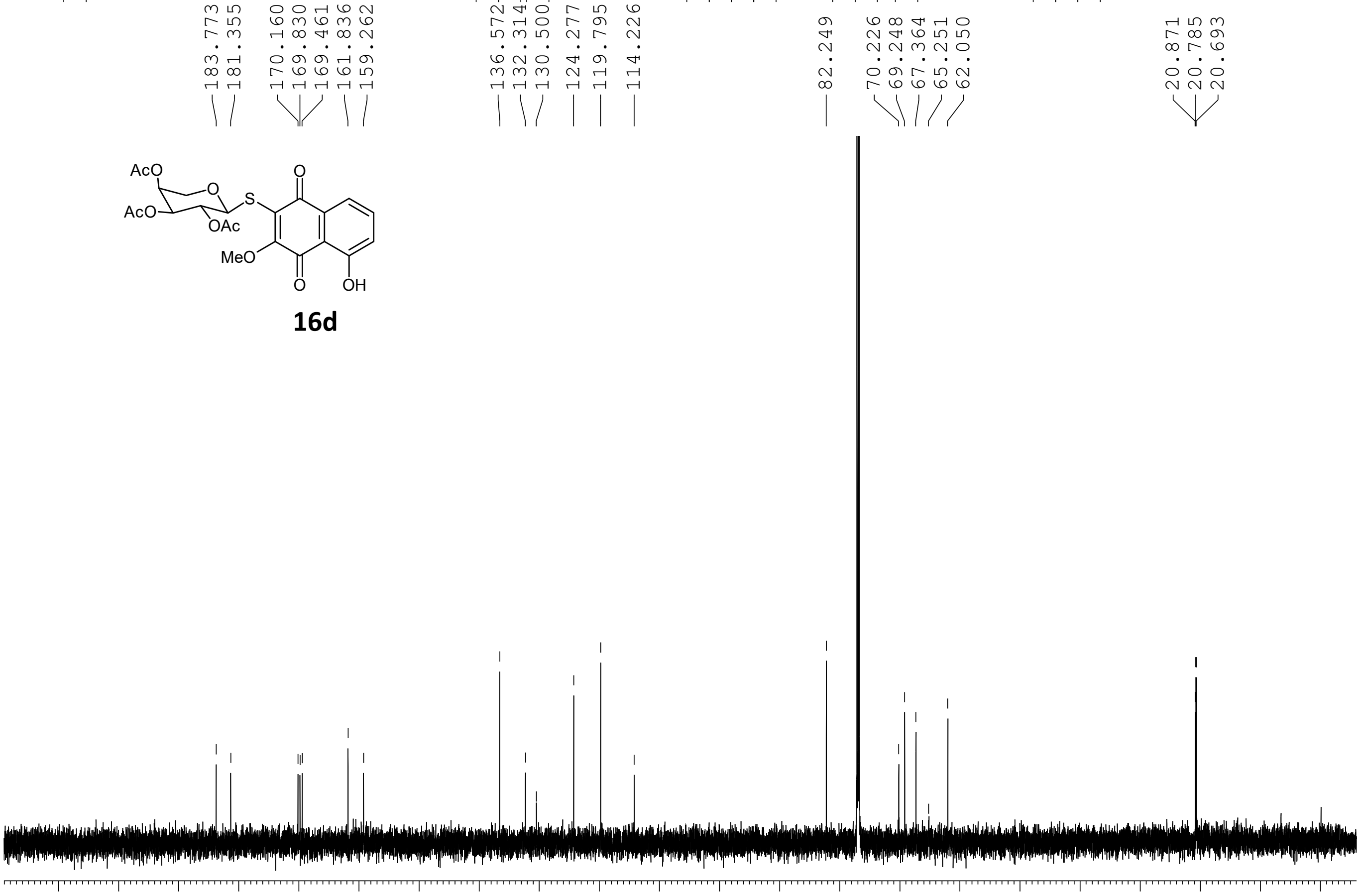

(2R,3S,4S,4aR,12aS)-3,4-Dihydroxy-2-hydroxymethyl-7,10-dimethoxy-3,4,4a,12a-tetrahydro-2H-naphtho[2,3-b]pyrano[2,3-e][1,4]oxathiine-6,11-dione ***(17a)***, (¹H NMR - 700 MHz, ¹³C NMR - 176 MHz, solvent - DMSO-d₆)

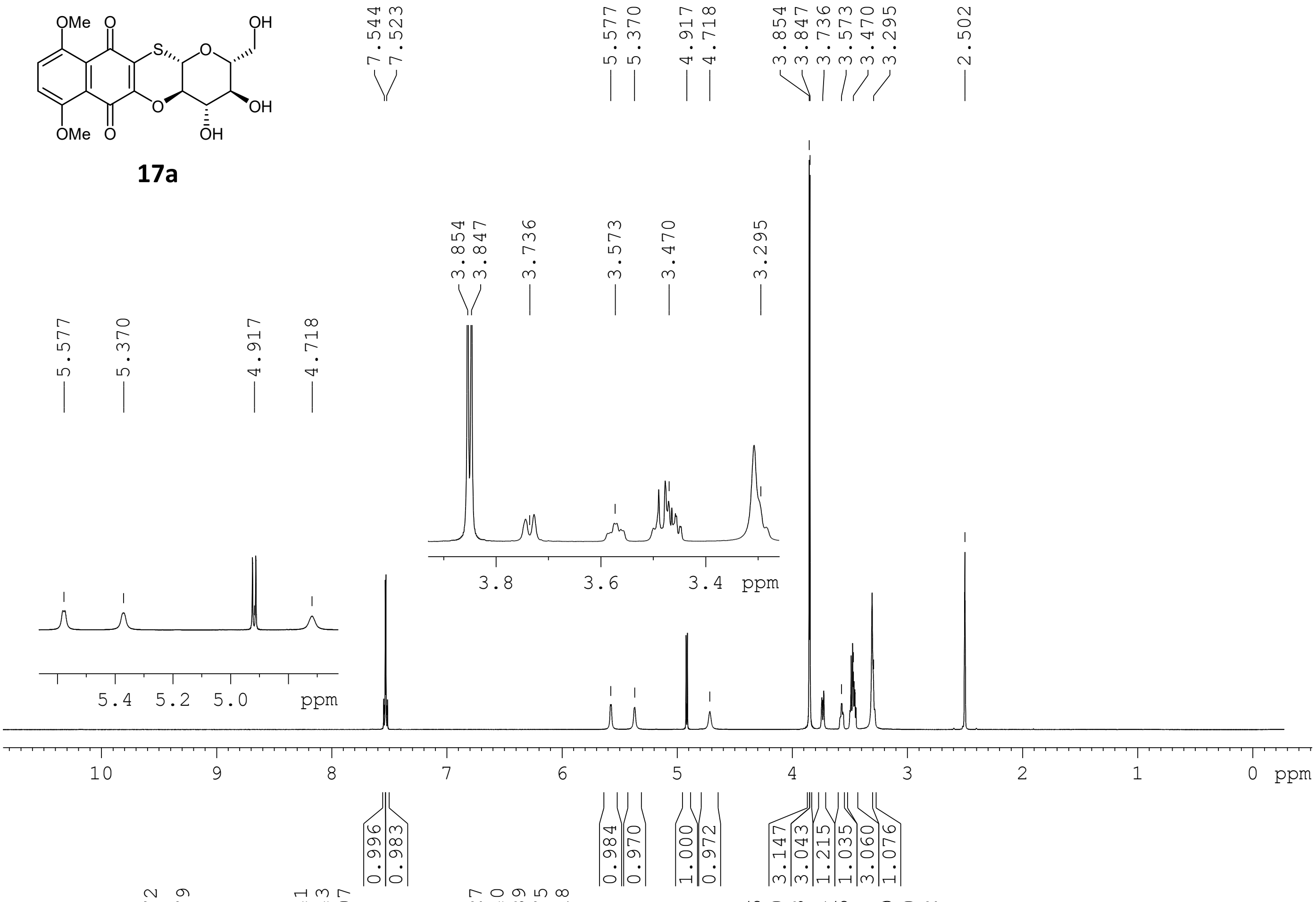

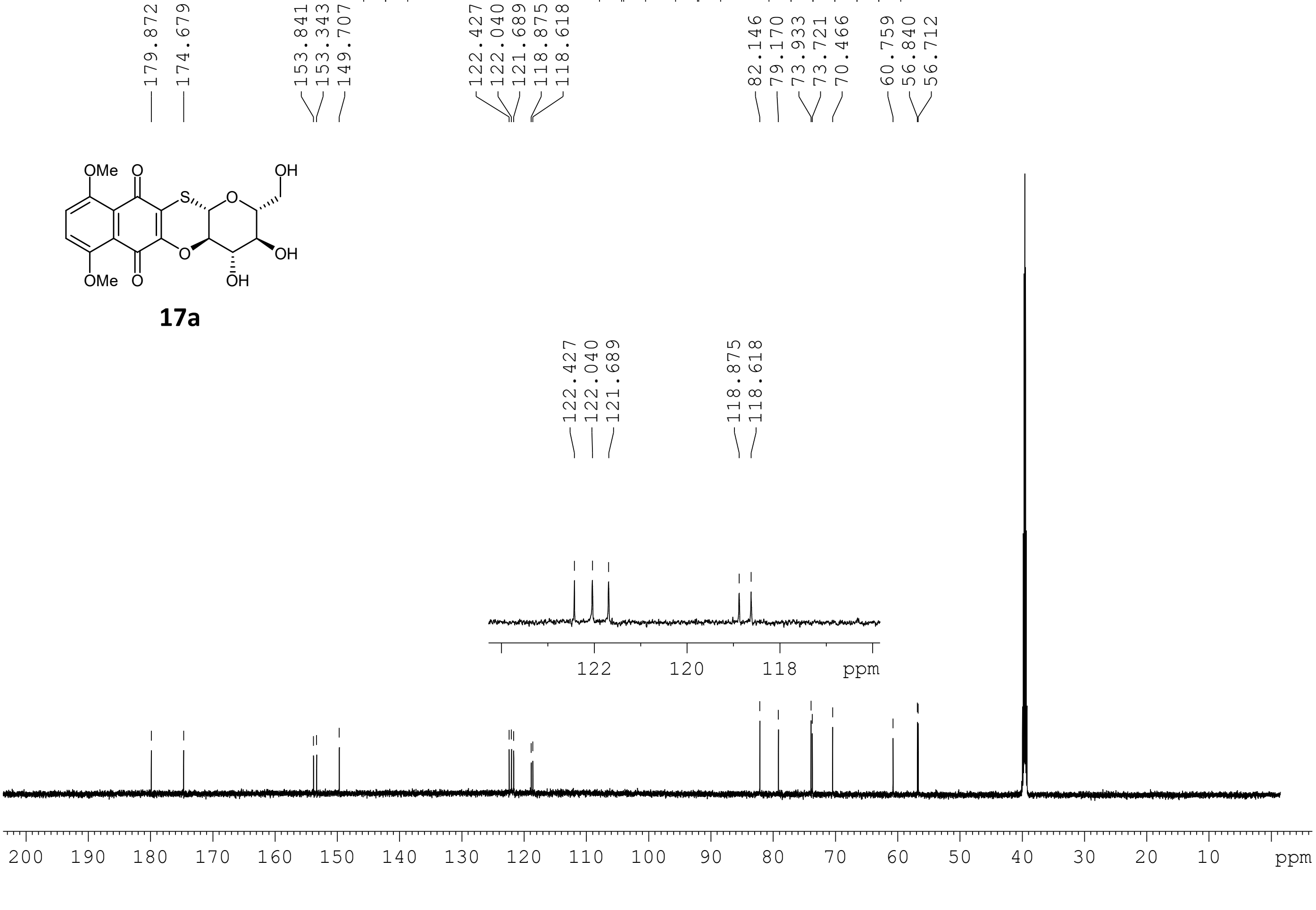

(2R,3R,4S,4aR,12aS)-3,4-Dihydroxy-2-hydroxymethyl-7,10-dimethoxy-3,4,4a,12a-tetrahydro-2H-naphtho[2,3-b]pyrano[2,3-e][1,4]oxathiine-6,11-dione ***(17b)***, (*^{1}H NMR - 500 MHz, ^{13}C NMR - 125 MHz, solvent - DMSO-d$_6$*)

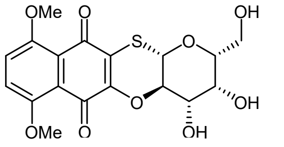

17b

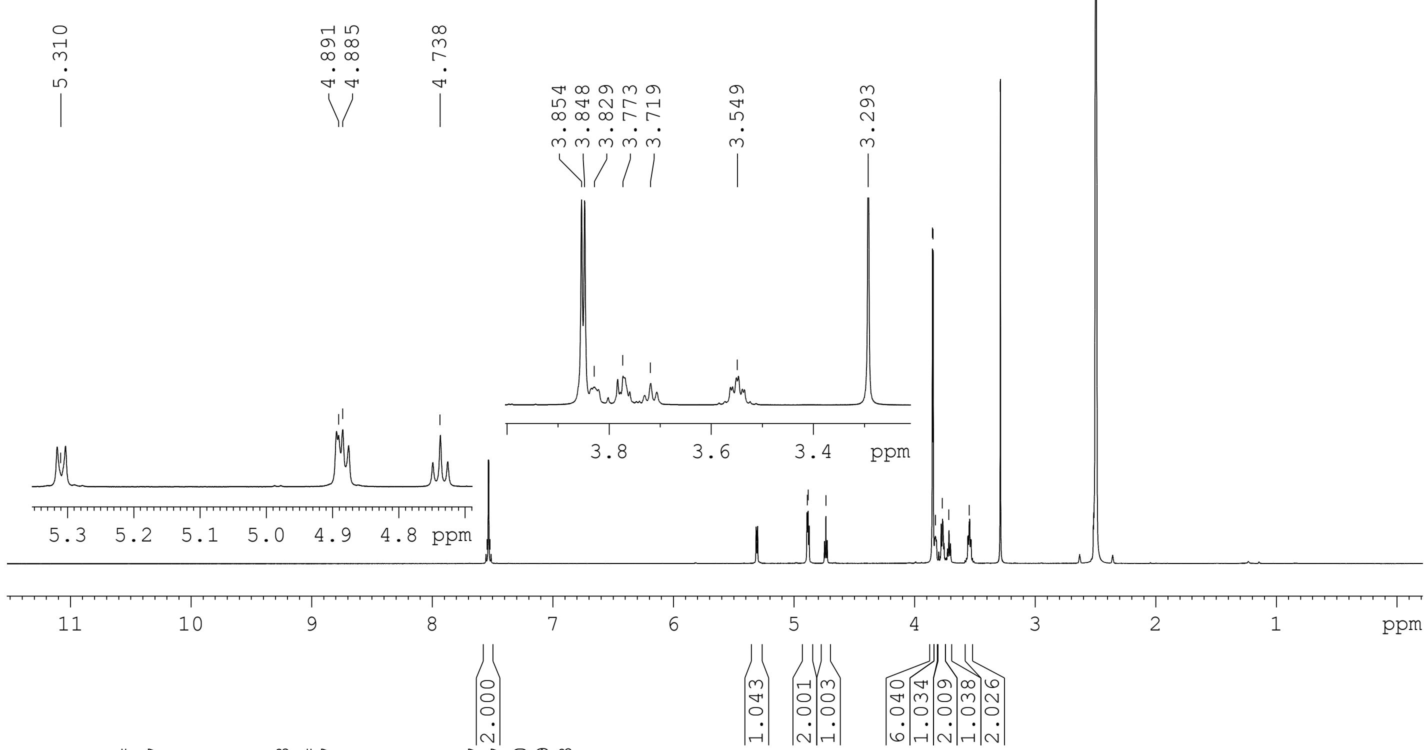

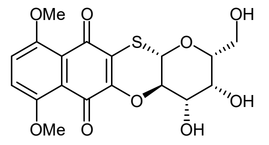

17b

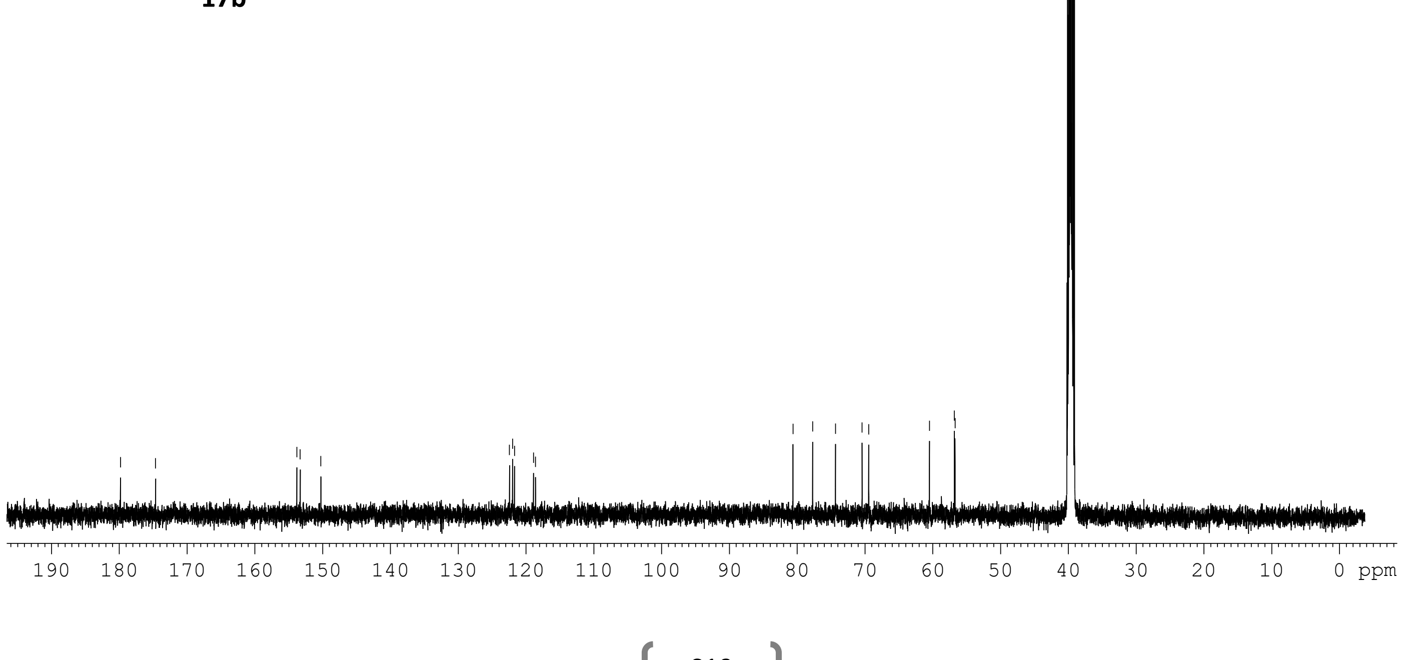

(3R,4S,4aR,12aS)-3,4-Dihydroxy-7,10-dimethoxy-3,4,4a,12a-tetrahydro-2H-naphtho[2,3-b]pyrano[2,3-e][1,4]oxathiine-6,11-dione ***(17c)****, (^{1}H NMR - 700 MHz, ^{13}C NMR - 176 MHz, solvent - DMSO-d$_6$)*

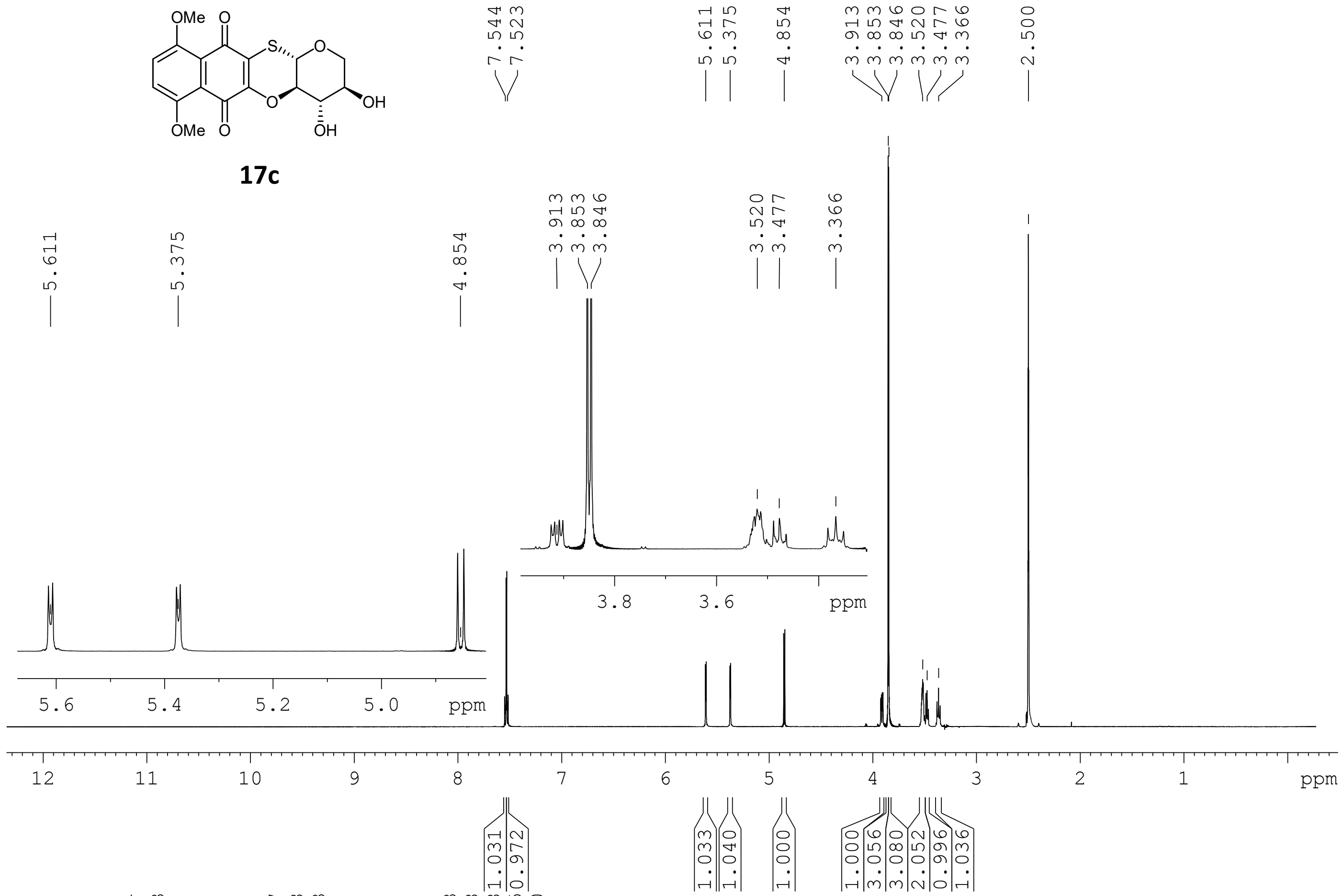

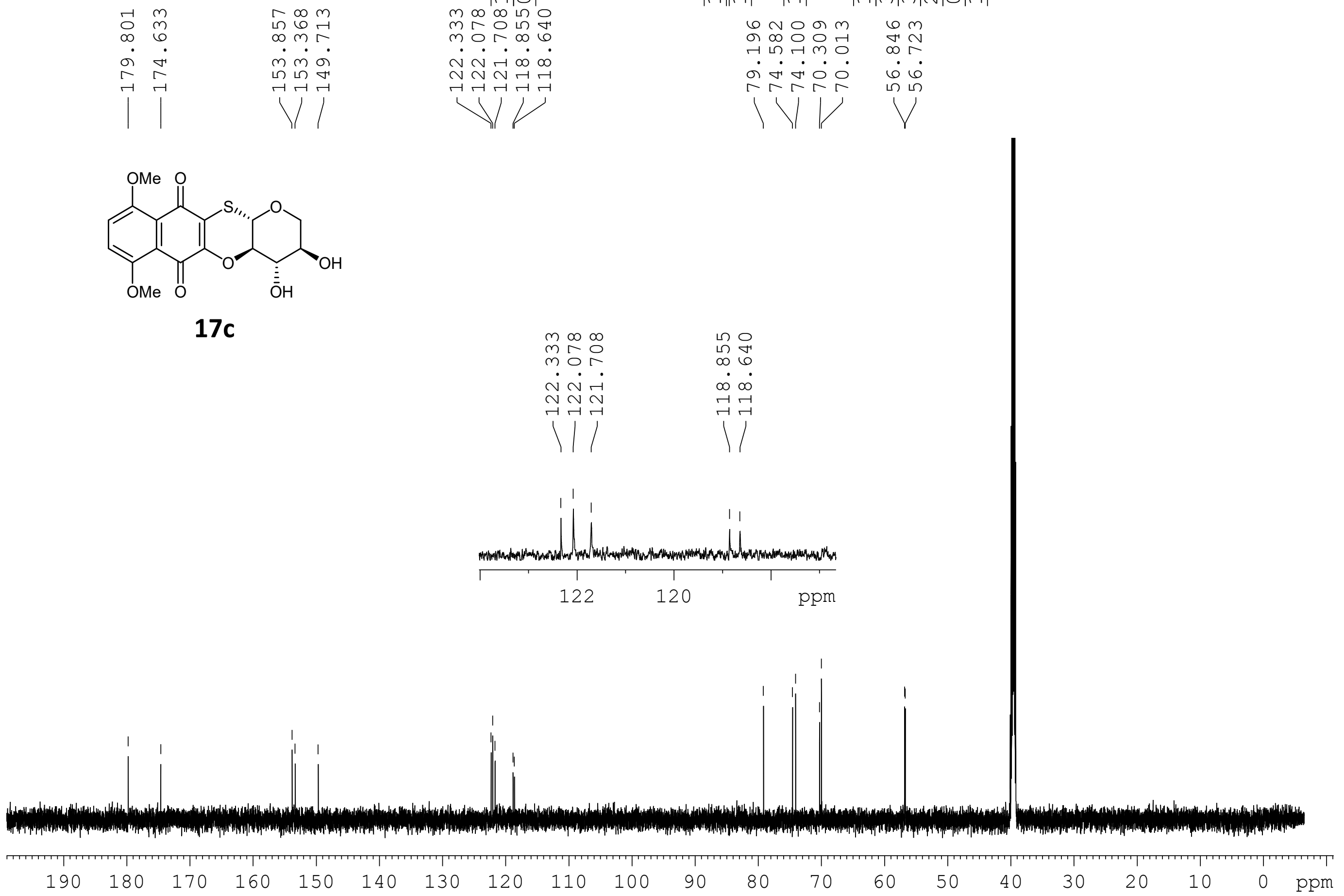

(3S,4S,4aR,12aS)-3,4-Dihydroxy-7,10-dimethoxy-3,4,4a,12a-tetrahydro-2H-naphtho[2,3-b]pyrano[2,3-e][1,4]oxathiine-6,11-dione ***(17d)***, *(^{1}H NMR - 700 MHz, ^{13}C NMR - 176 MHz, solvent - DMSO-d_6)*

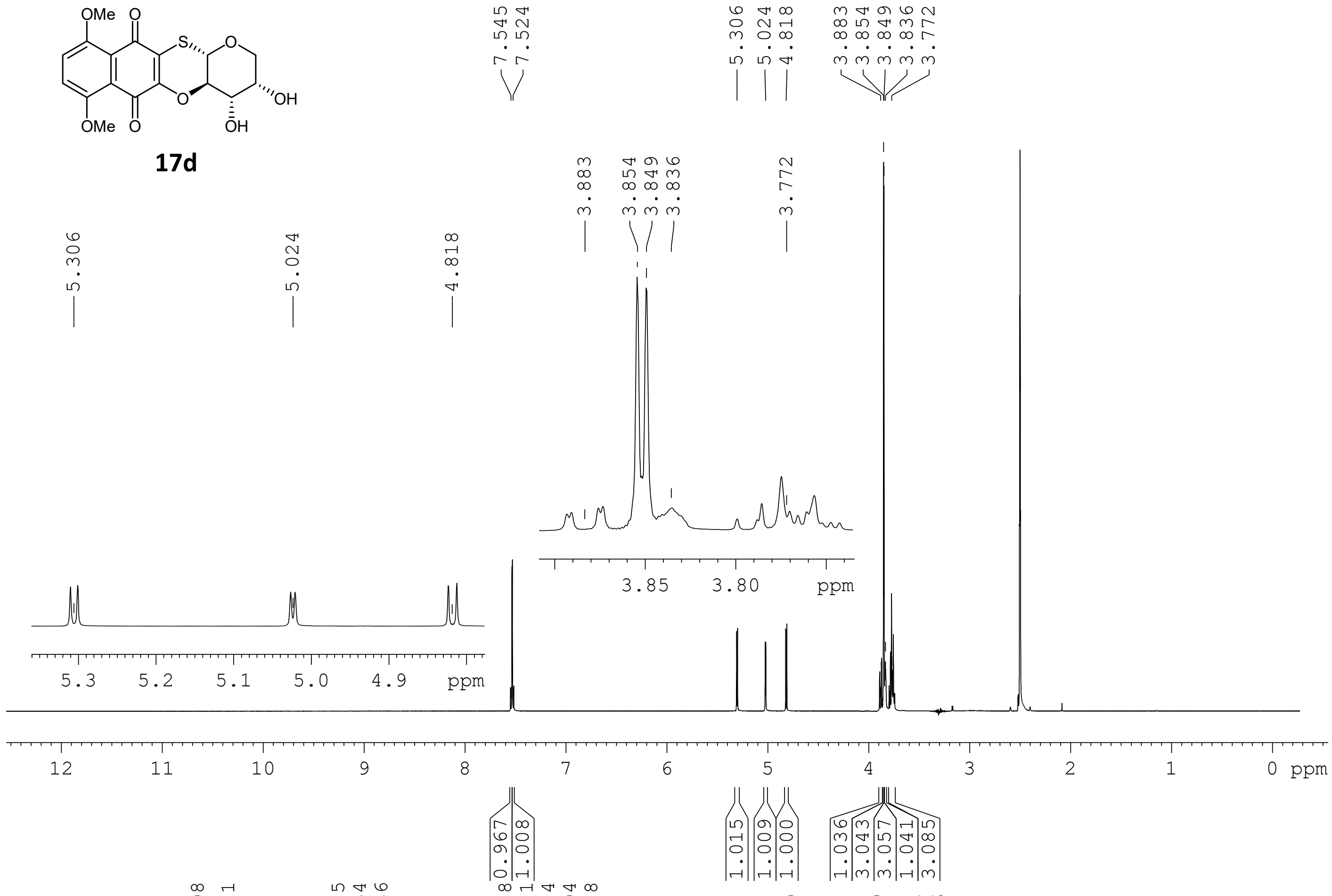

17d

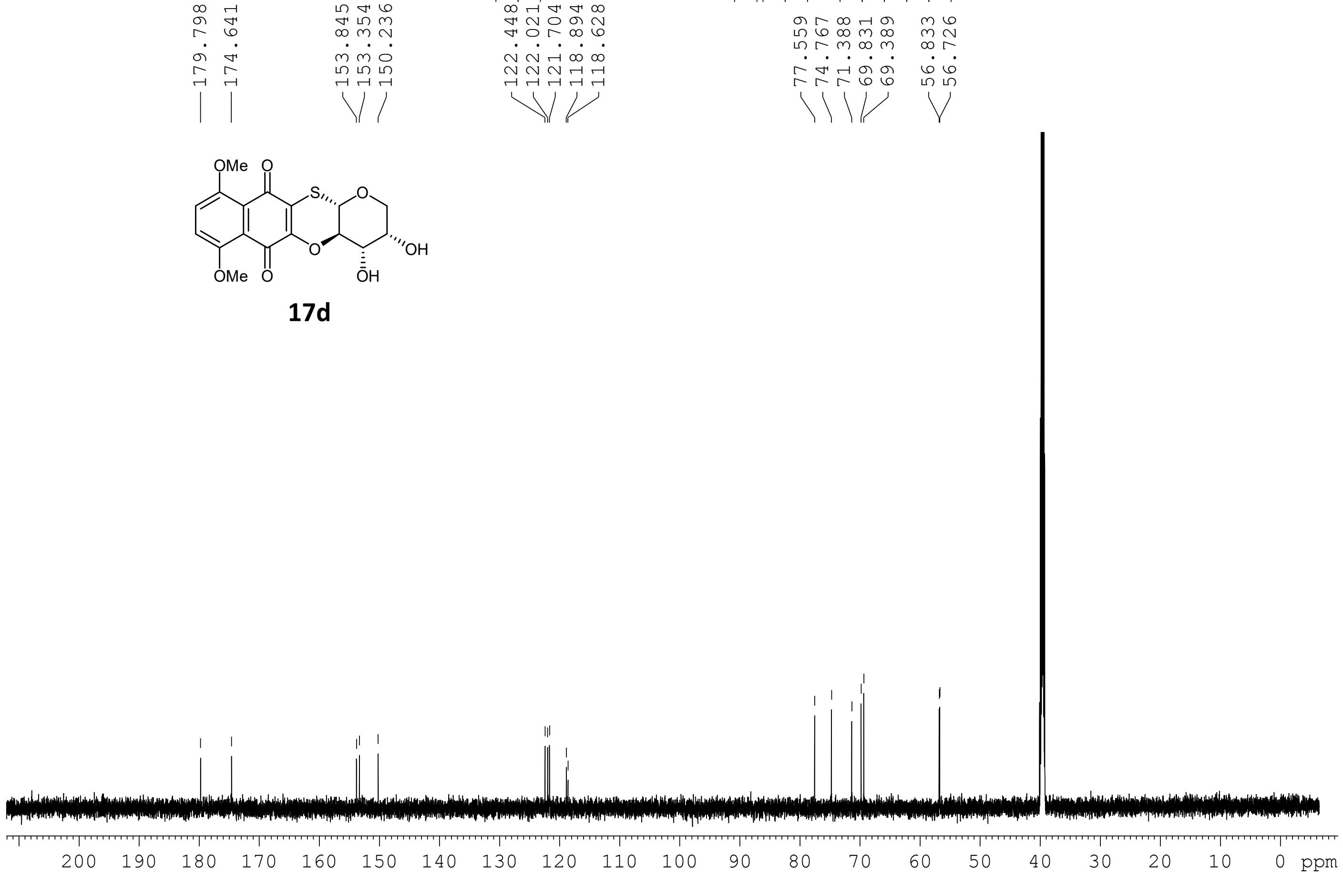

17d

(2R,3S,4S,4aR,12aS)-8,9-Dichloro-3,4-dihydroxy-2-hydroxymethyl-7,10-dimethoxy-3,4,4a,12a-tetrahydro-2H-naphtho[2,3-b]pyrano[2,3-e][1,4]oxathiine-6,11-dione ***(18a)***, *(^{1}H NMR - 500 MHz, ^{13}C NMR - 125 MHz, solvent - DMSO-d_6)*

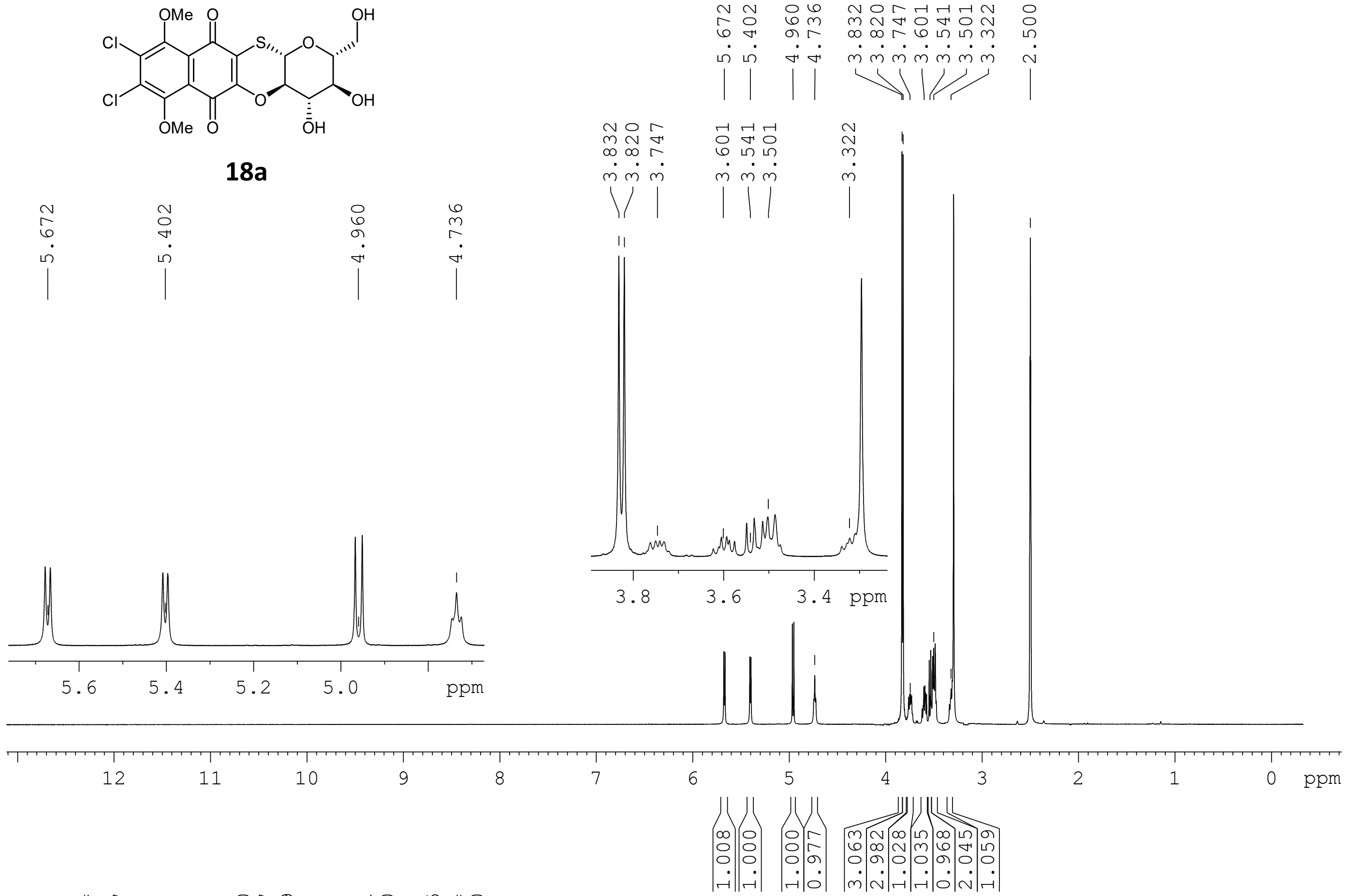

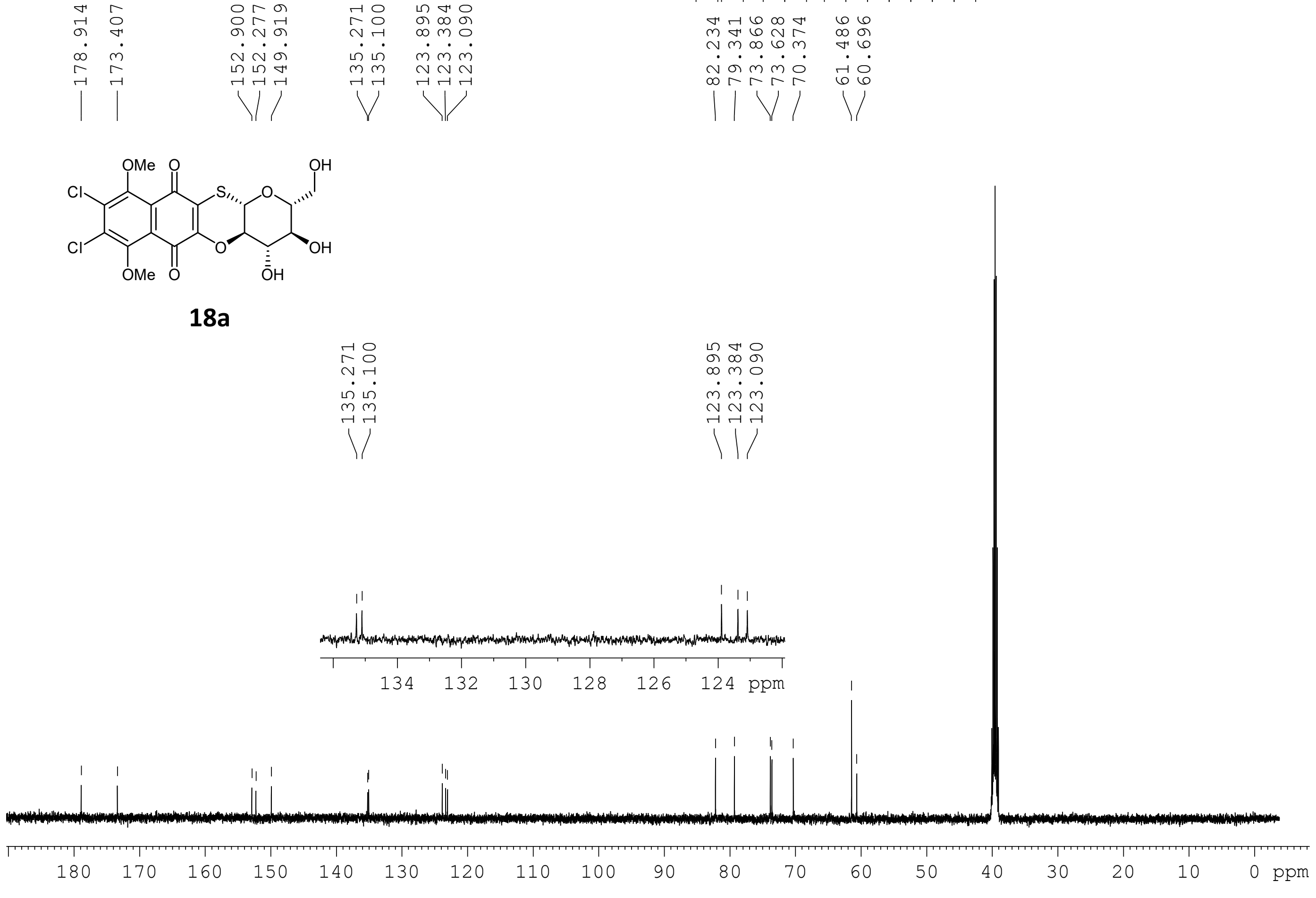

(2R,3R,4S,4aR,12aS)-8,9-Dichloro-3,4-dihydroxy-2-(hydroxymethyl)-7,10-dimethoxy-3,4,4a,12a-tetrahydro-2H-naphtho[2,3-b]pyrano[2,3-e][1,4]oxathiine-6,11-dione ***(18b)***, *(^{1}H NMR - 700 MHz, ^{13}C NMR - 176 MHz, solvent - DMSO-d_6)*

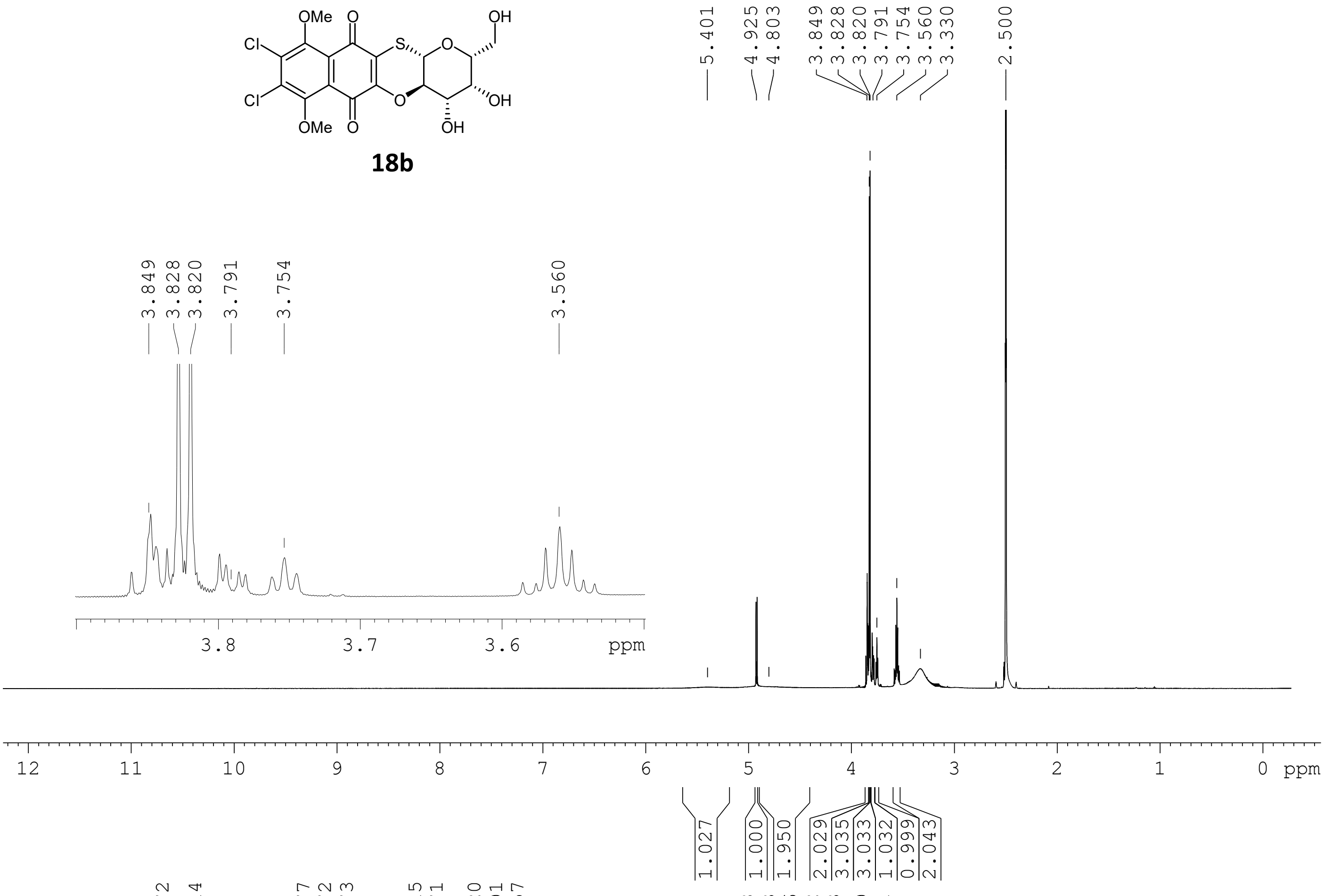

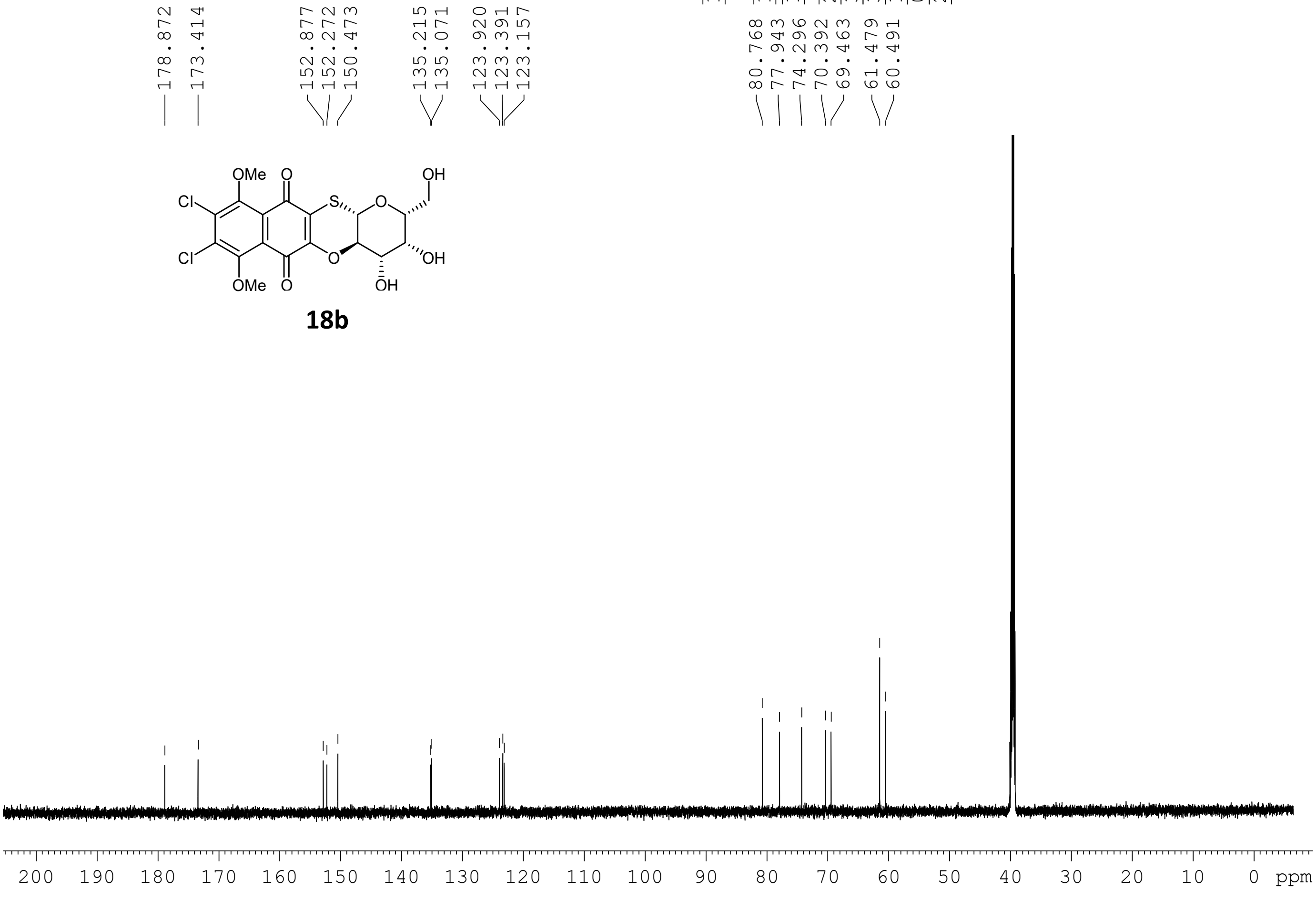

(3R,4S,4aR,12aS)-8,9-Dichloro-3,4-dihydroxy-7,10-dimethoxy-3,4,4a,12a-tetrahydro-2H-naphtho[2,3-b]pyrano[2,3-e][1,4]oxathiine-6,11-dione ***(18c)****, (^{1}H NMR - 700 MHz, ^{13}C NMR - 176 MHz, solvent - DMSO-d$_6$)*

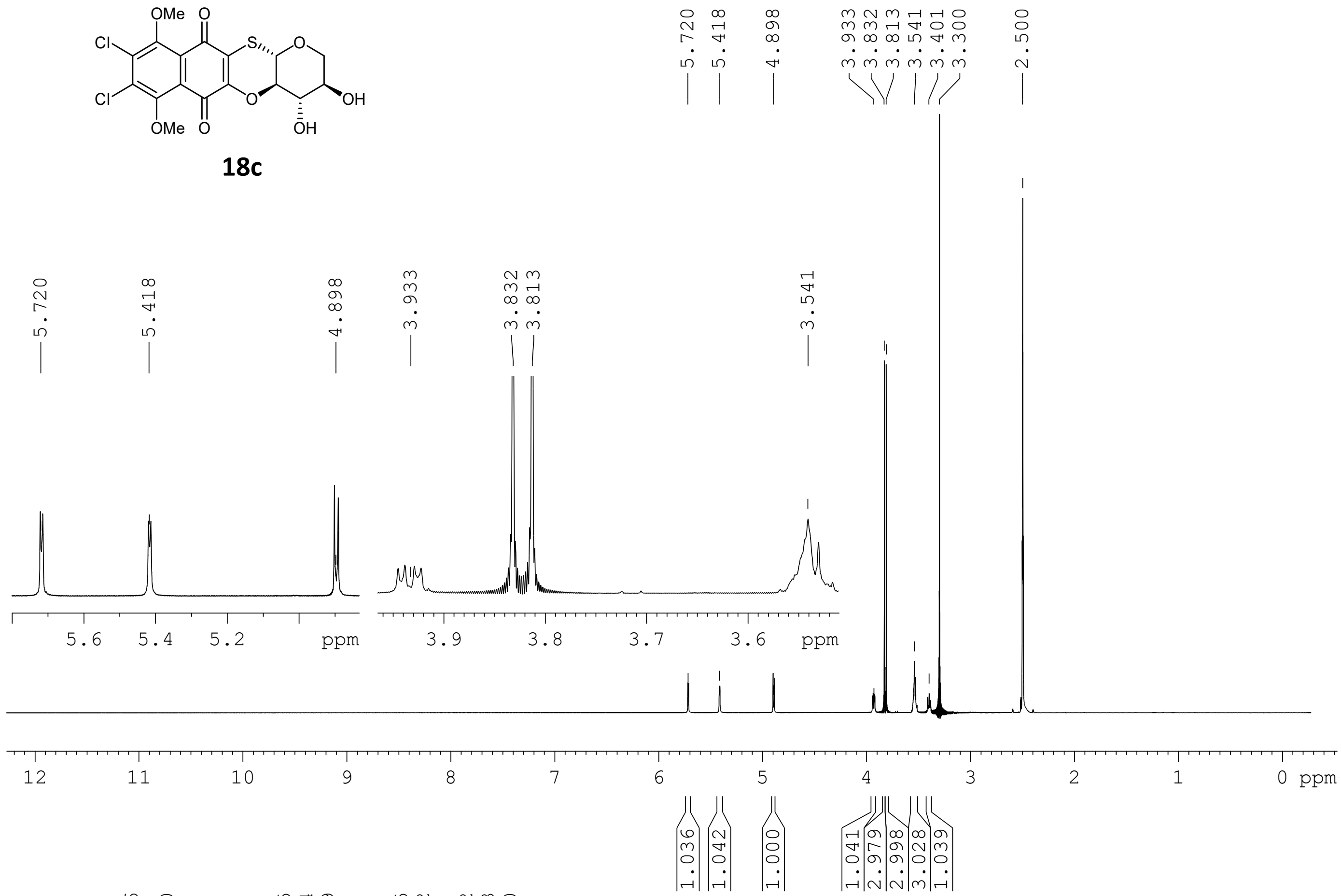

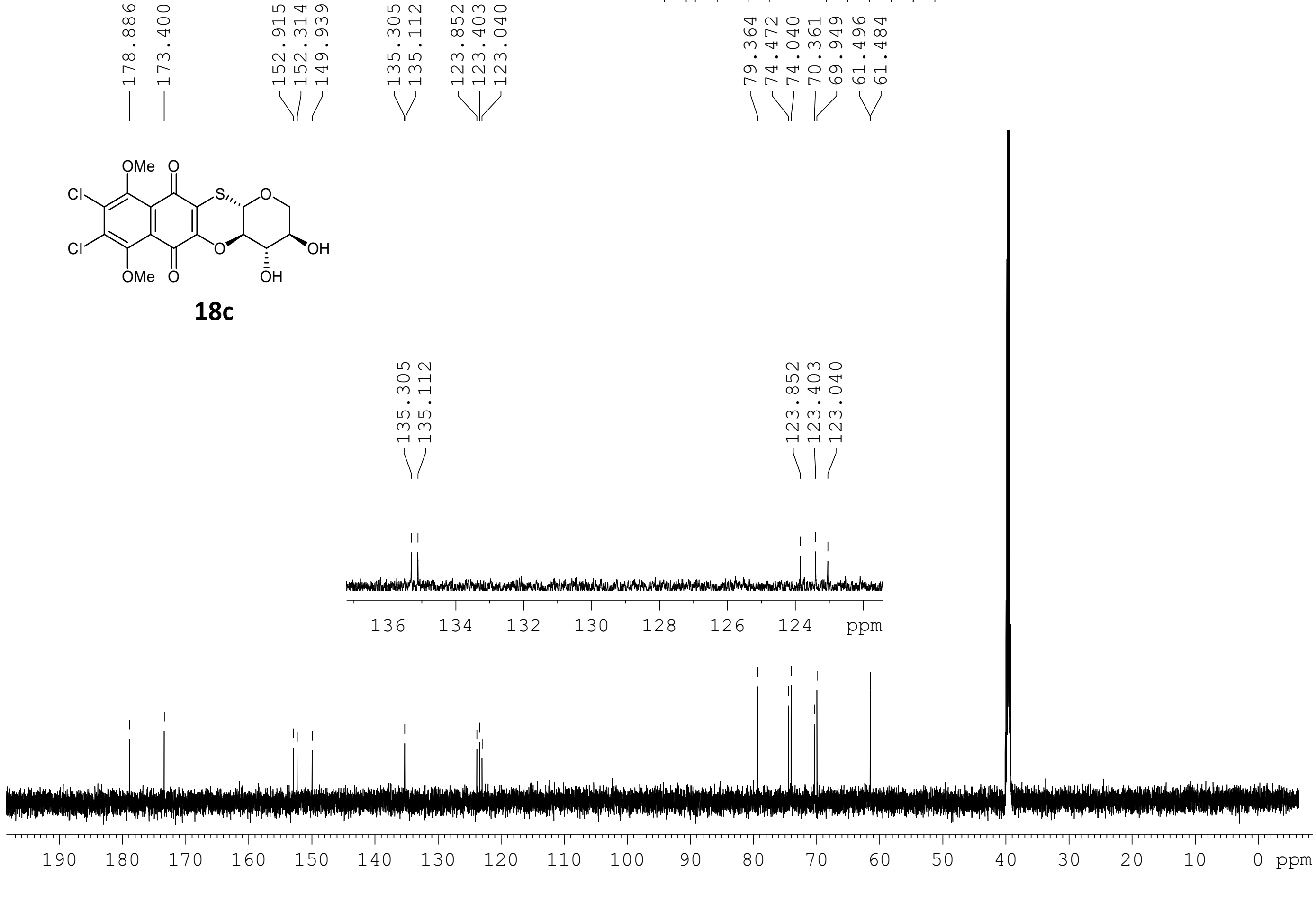

*(3S,4S,4aR,12aS)-8,9-Dichloro-3,4-dihydroxy-7,10-dimethoxy-3,4,4a,12a-tetrahydro-2H-naphtho[2,3-b]pyrano[2,3-e][1,4]oxathiine-6,11-dione **(18d)**, (^{1}H NMR - 500 MHz, ^{13}C NMR - 125 MHz, solvent - DMSO-d$_6$)*

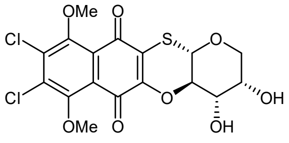

18d

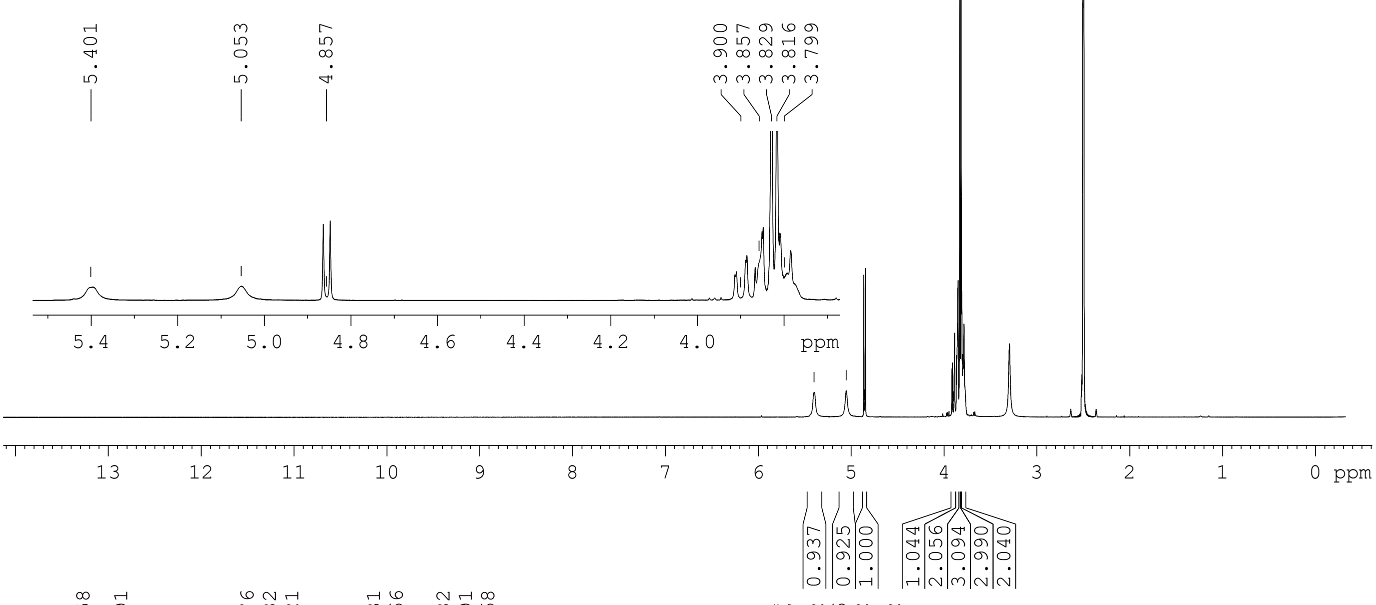

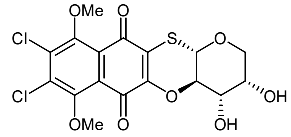

18d

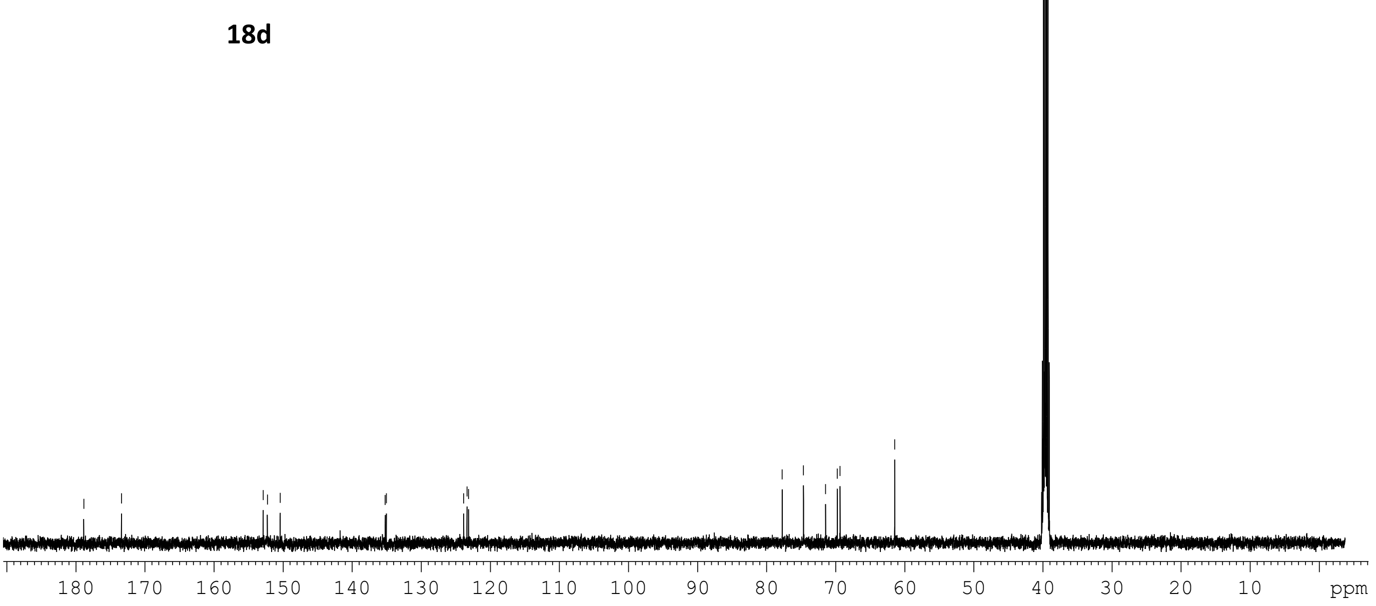

(2R,3S,4S,4aR,12aS)-3,4,10-Trihydroxy-2-hydroxymethyl-3,4,4a,12a-tetrahydro-2H-naphtho[2,3-b]pyrano[2,3-e][1,4]oxathiine-6,11-dione ***(19a)***, *(^{1}H NMR - 700 MHz, ^{13}C NMR - 176 MHz, solvent - DMSO-d$_6$)*

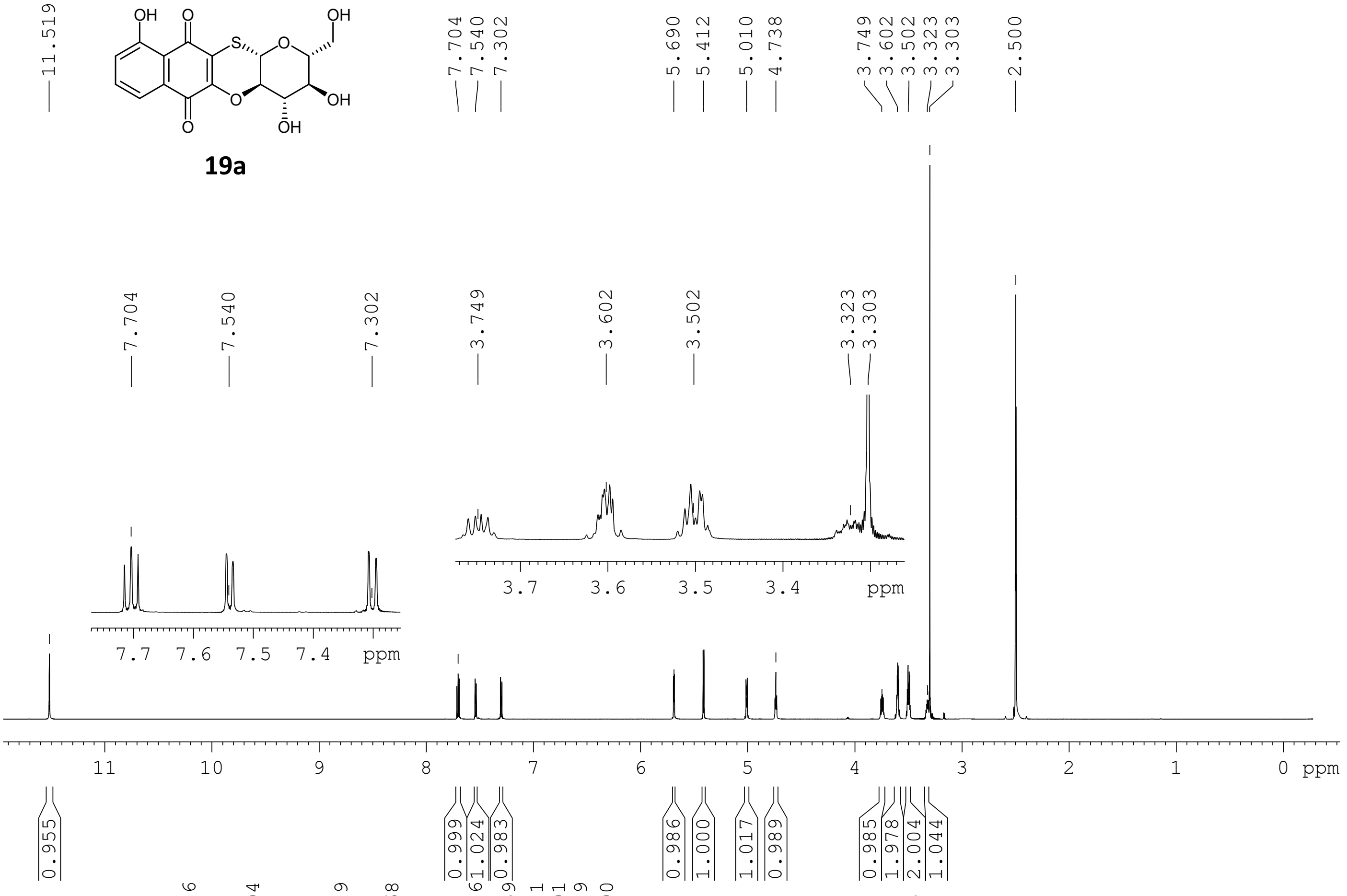

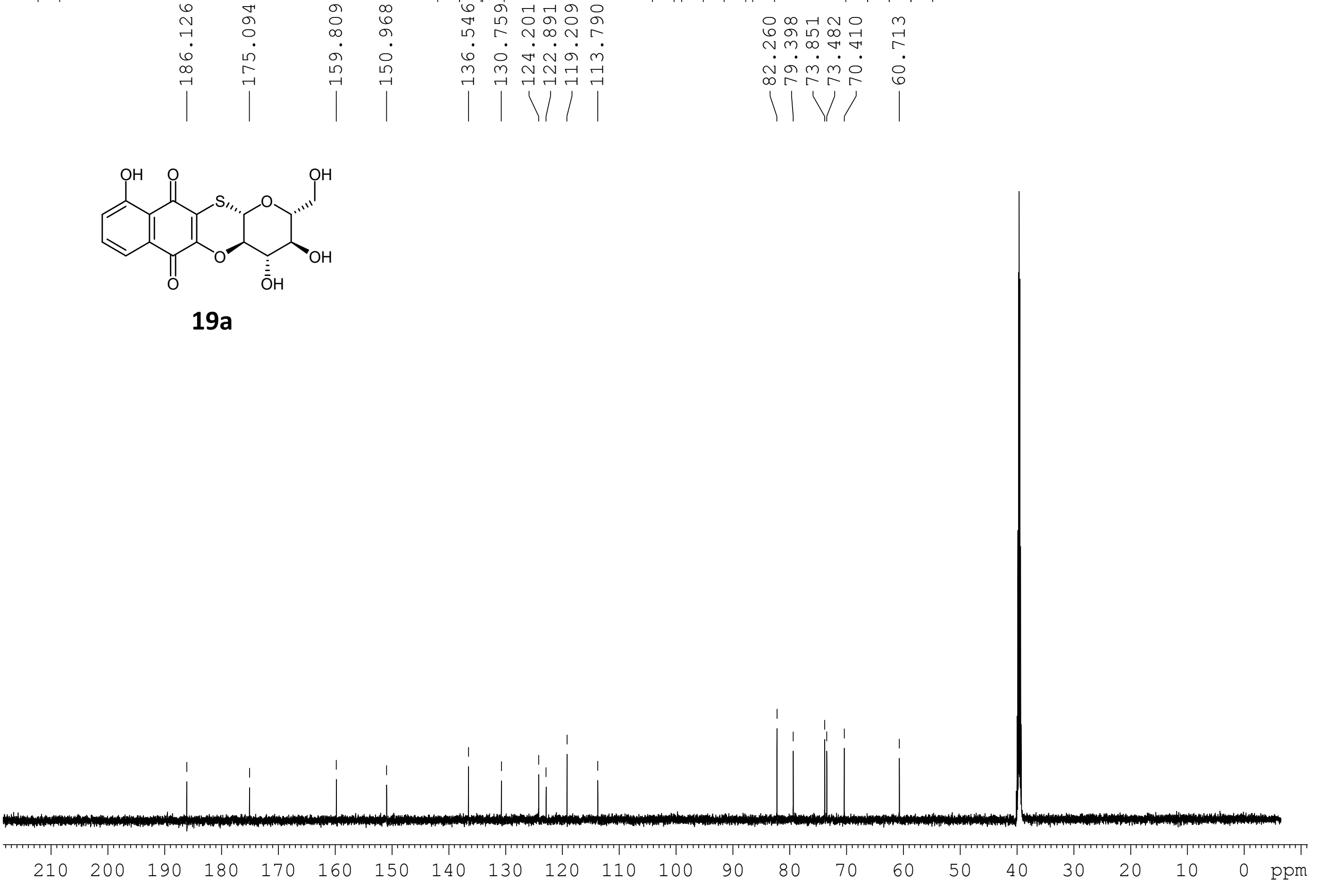

(2R,3R,4S,4aR,12aS)-3,4,10-Trihydroxy-2-hydroxymethyl-3,4,4a,12a-tetrahydro-2H-naphtho[2,3-b]pyrano-[2,3-e][1,4]oxathiine-6,11-dione ***(19b)***, *(^{1}H NMR - 500 MHz, ^{13}C NMR - 125 MHz, solvent - DMSO-d₆)*

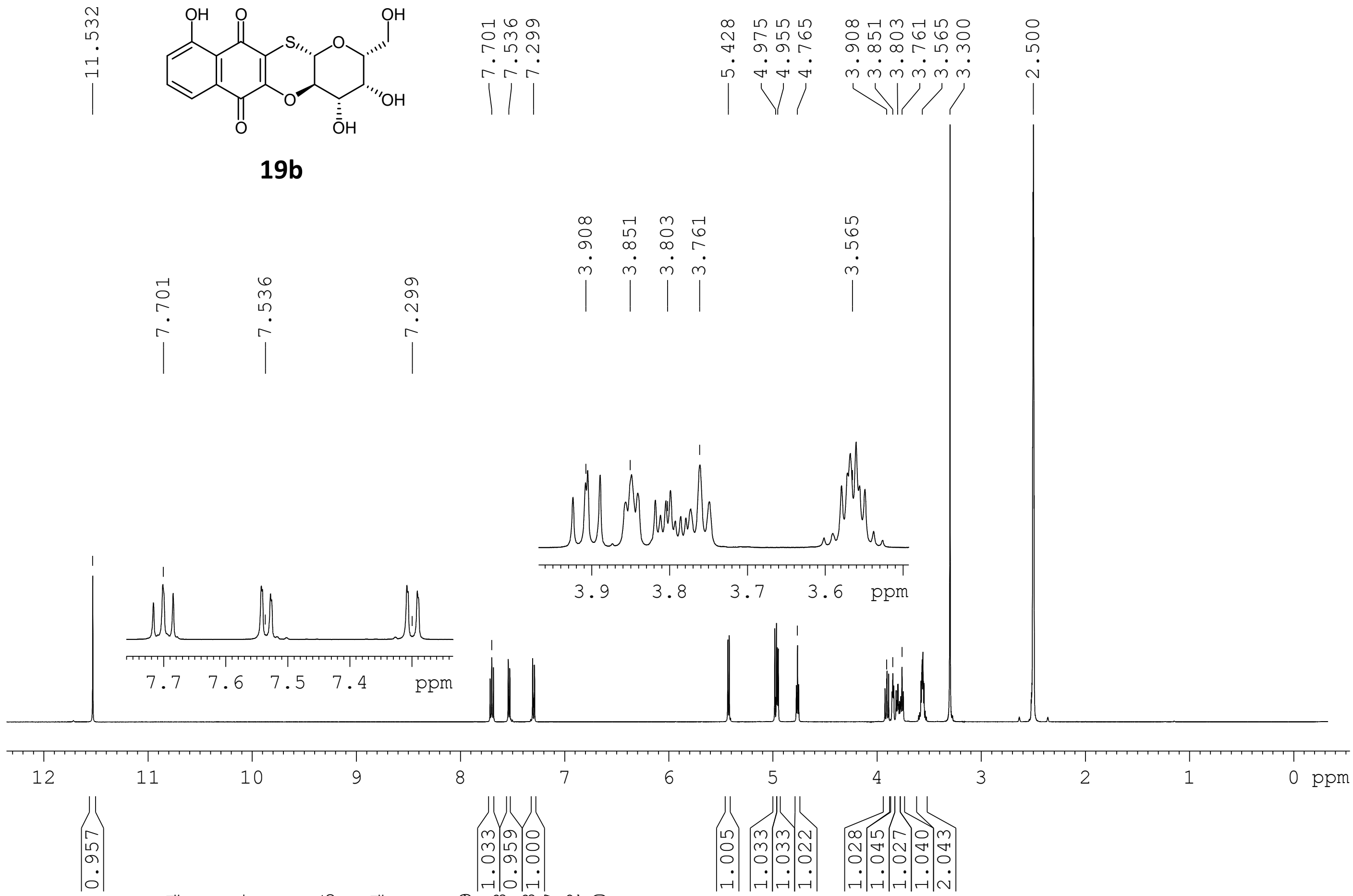

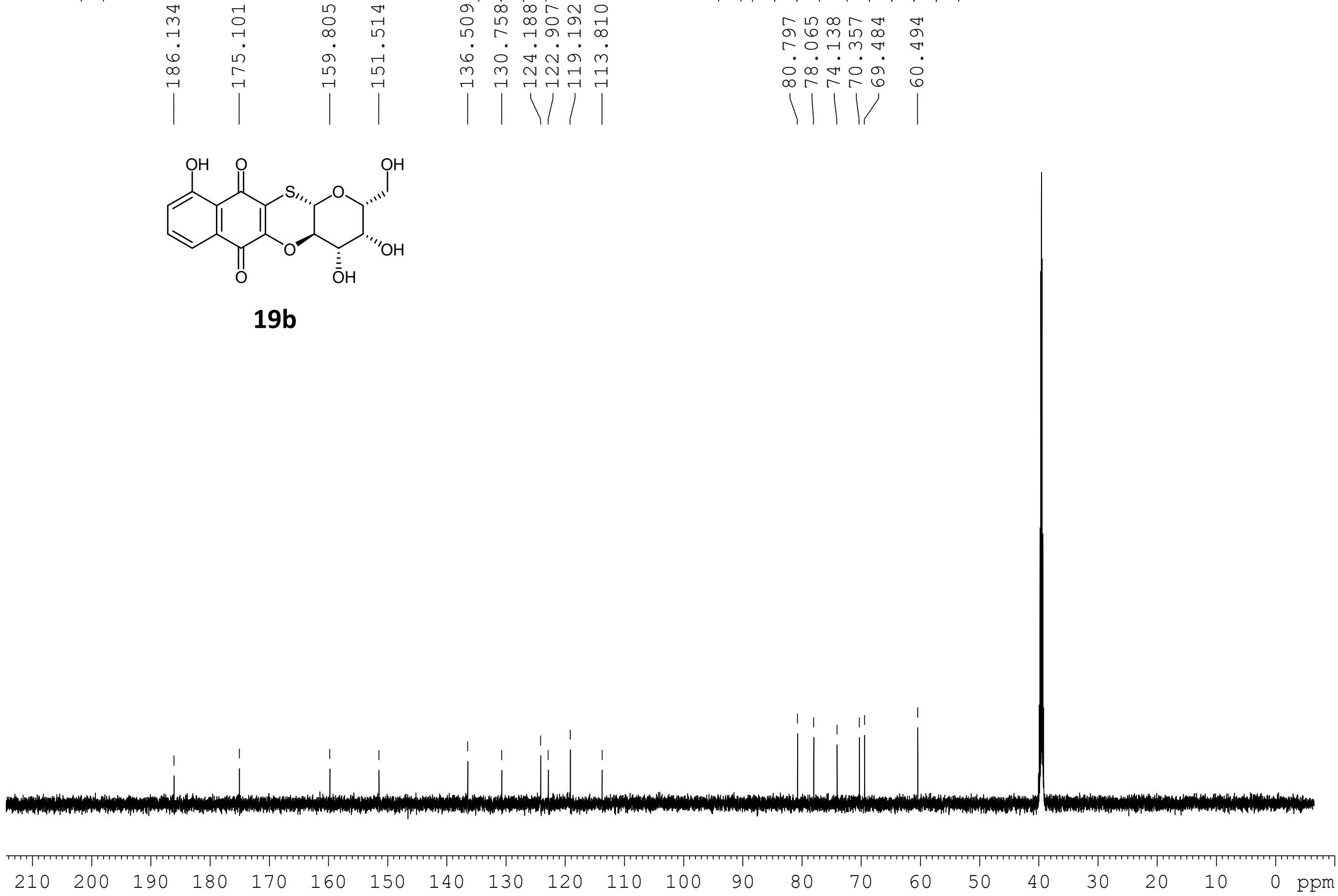

(3R,4S,4aR,12aS)-3,4,10-Trihydroxy-3,4,4a,12a-tetrahydro-2H-naphtho[2,3-b]pyrano[2,3-e][1,4]oxathiine-6,11-dione ***(19c)***, *(^{1}H NMR - 700 MHz, ^{13}C NMR - 176 MHz, solvent - DMSO-d6)*

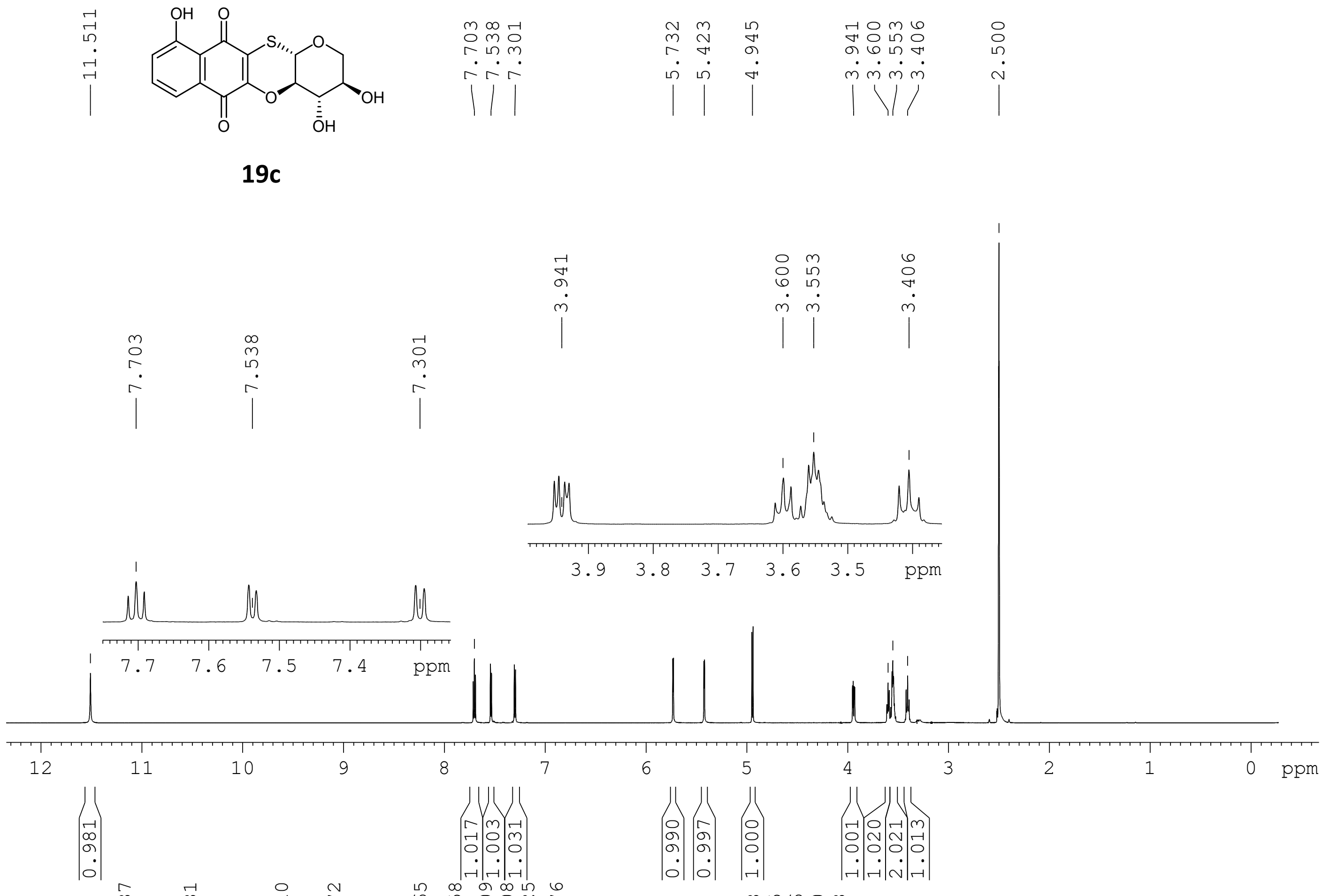

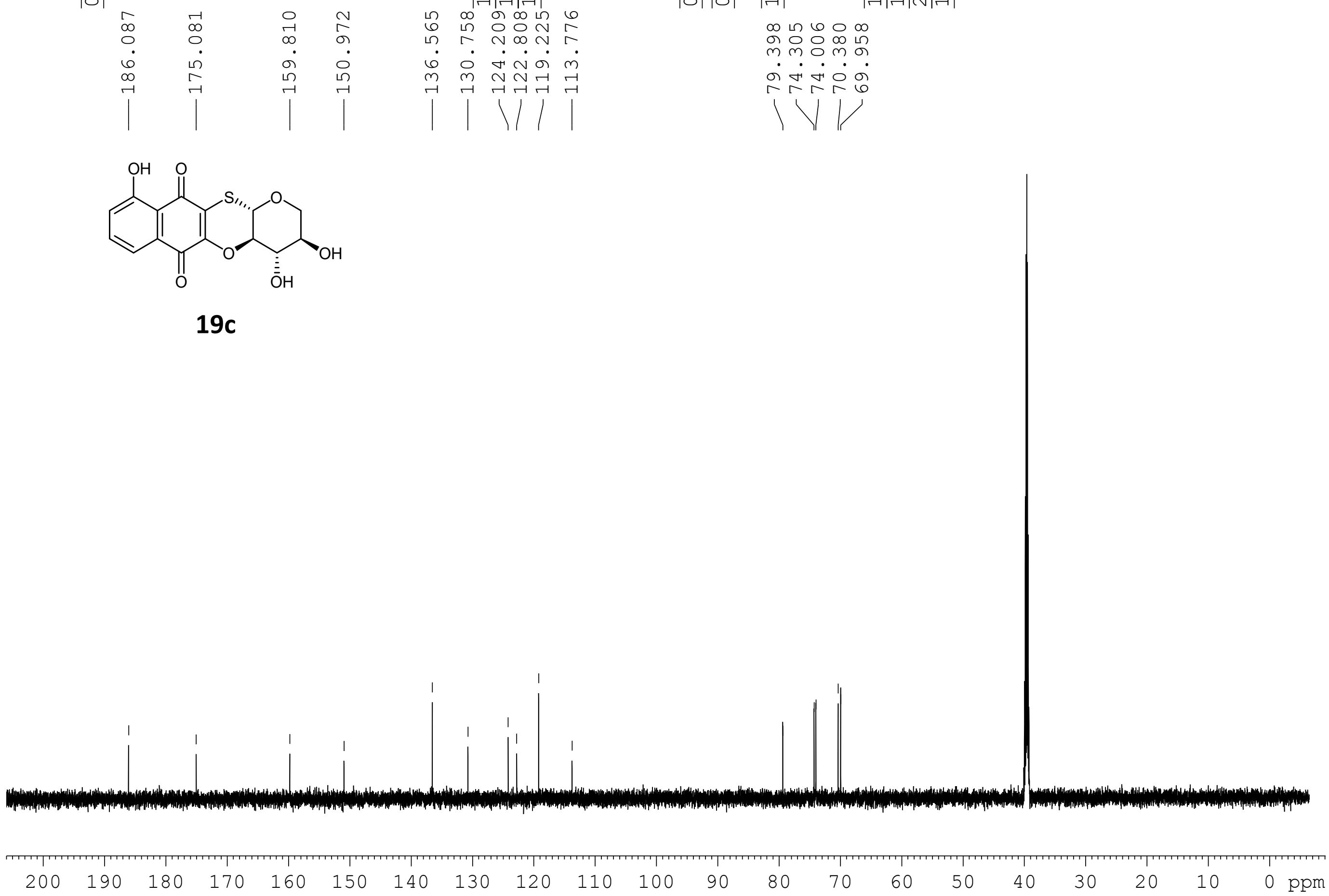

(3S,4S,4aR,12aS)-3,4,10-Trihydroxy-3,4,4a,12a-tetrahydro-2H-naphtho[2,3-b]pyrano[2,3-e][1,4]oxathiine-6,11-dione ***(19d)***, *(^{1}H NMR - 500 MHz, ^{13}C NMR - 125 MHz, solvent - DMSO-d_6)*

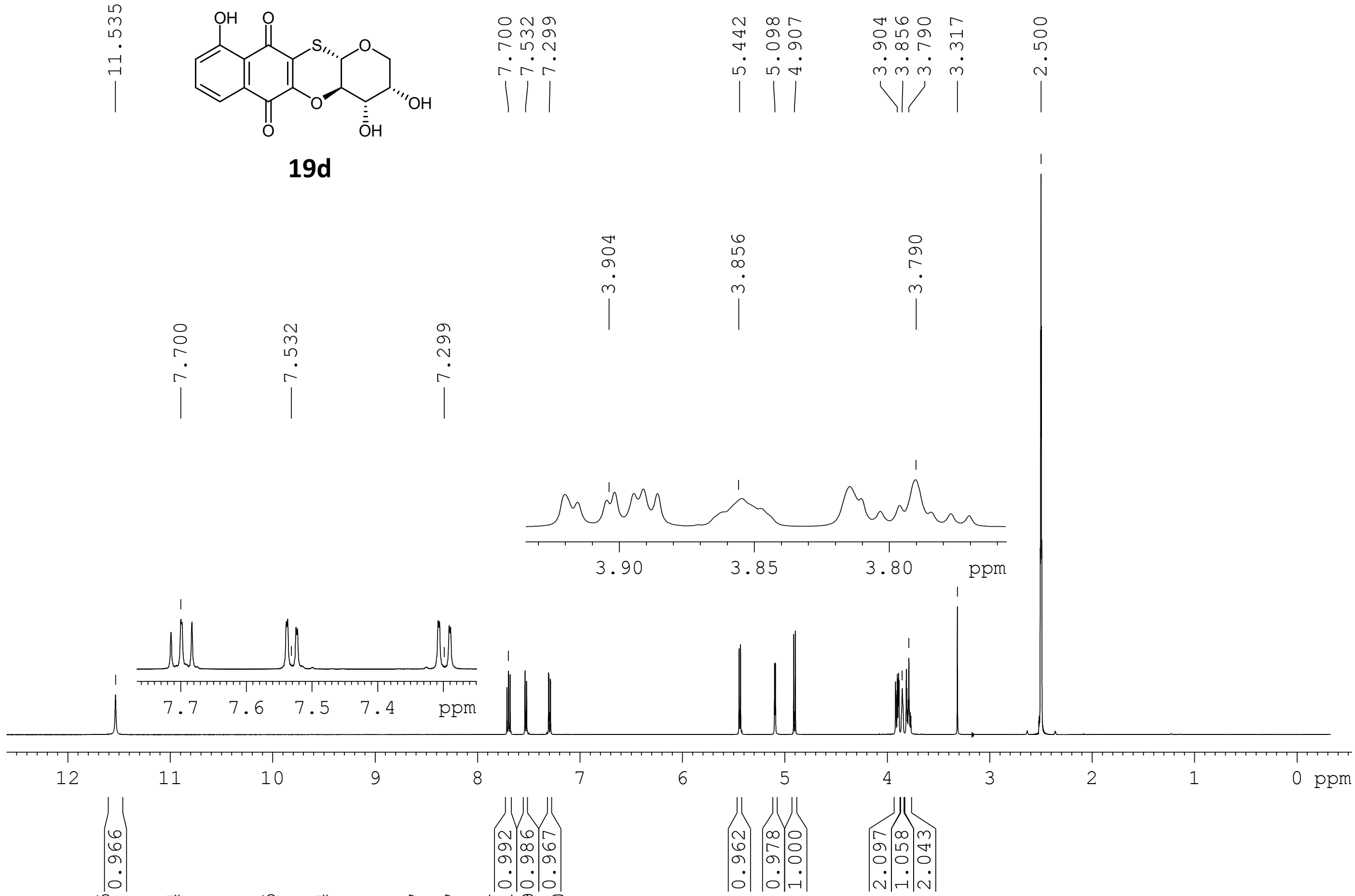

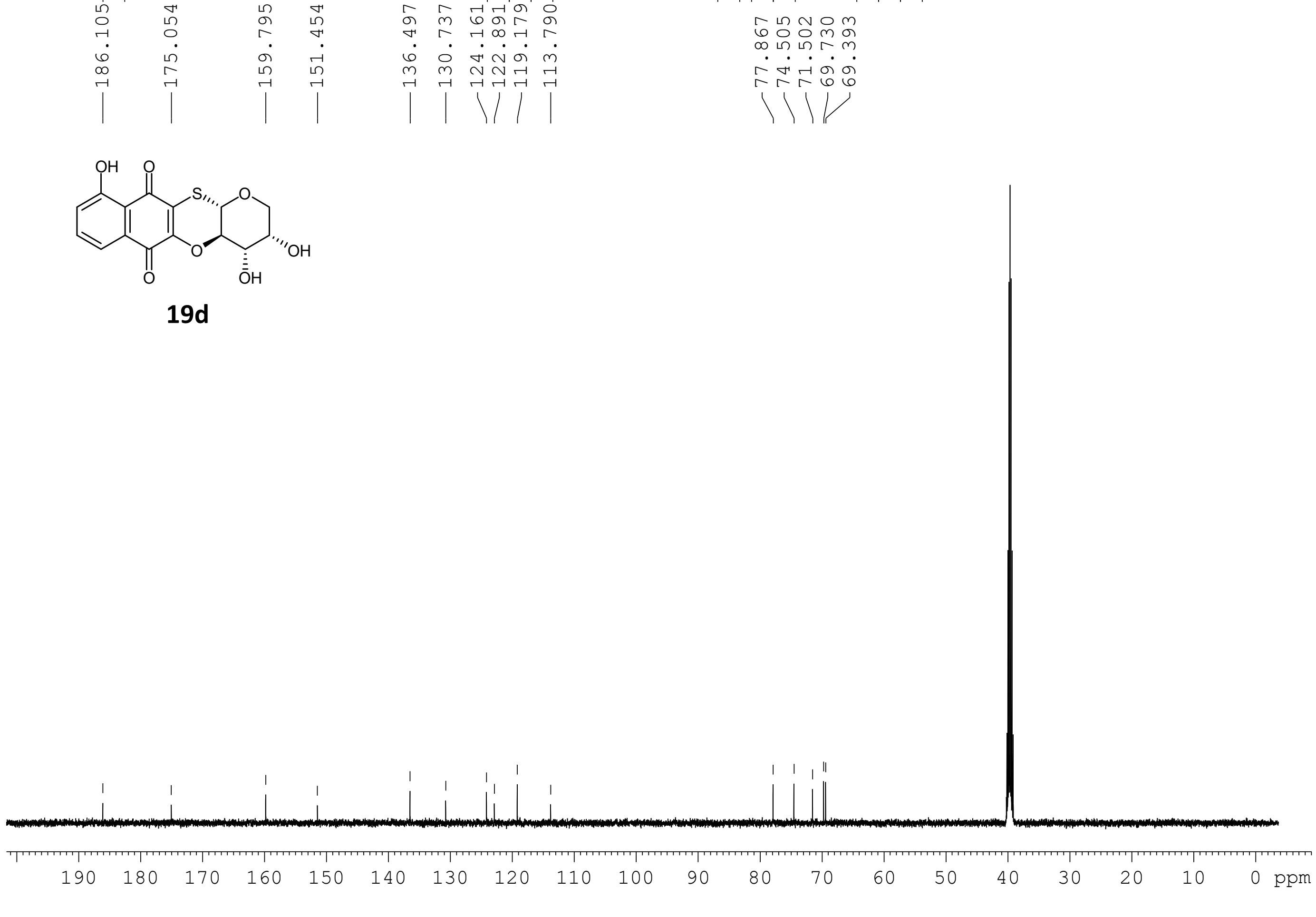

(2R,3S,4S,4aR,12aS)-3,4,7-Trihydroxy-2-(hydroxymethyl)-3,4,4a,12a-tetrahydro-2H-naphtho[2,3-b]pyrano-[2,3-e][1,4]oxathiine-6,11-dione ***(20a)***, *(^{1}H NMR - 700 MHz, ^{13}C NMR - 176 MHz, solvent - DMSO-d6)*

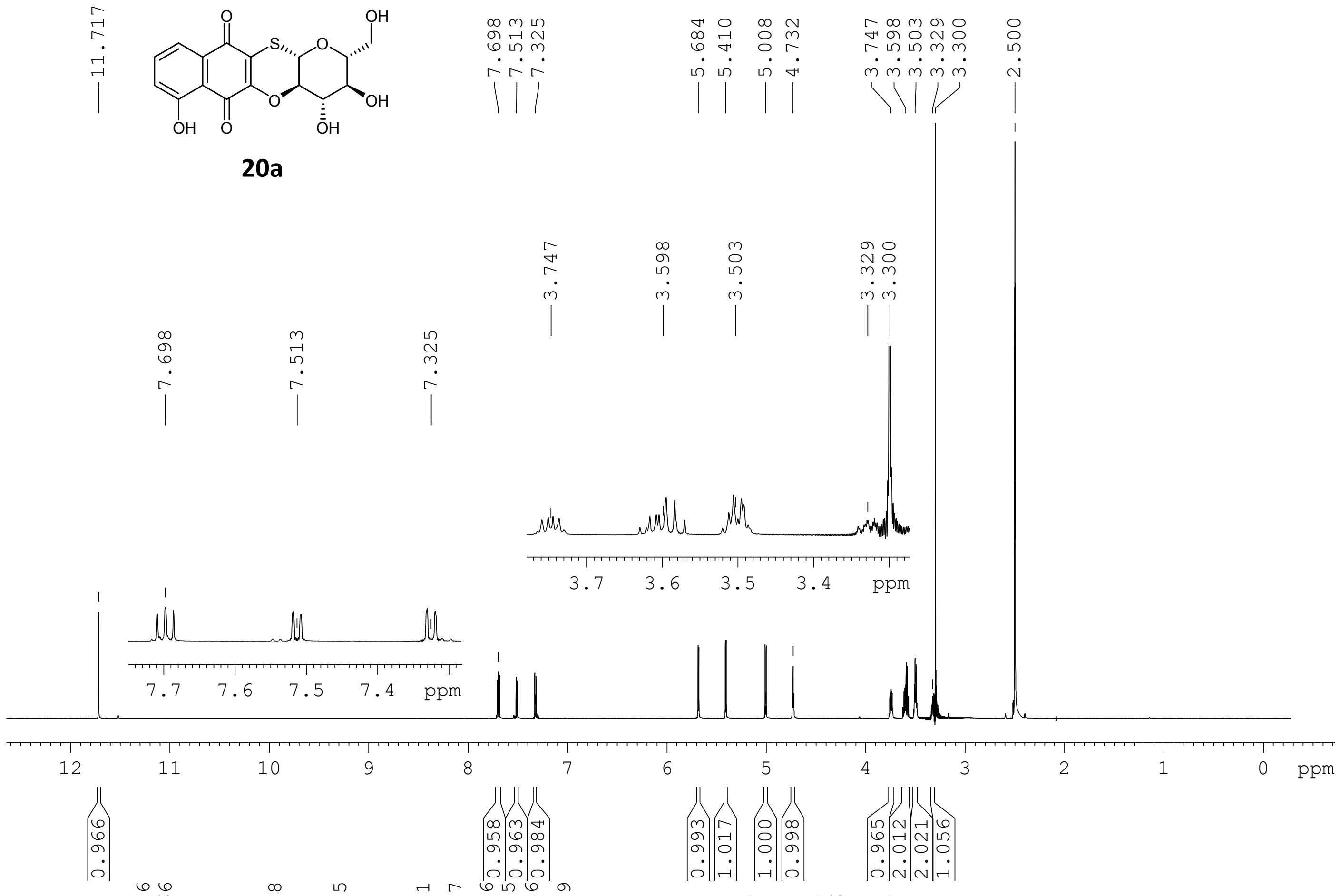

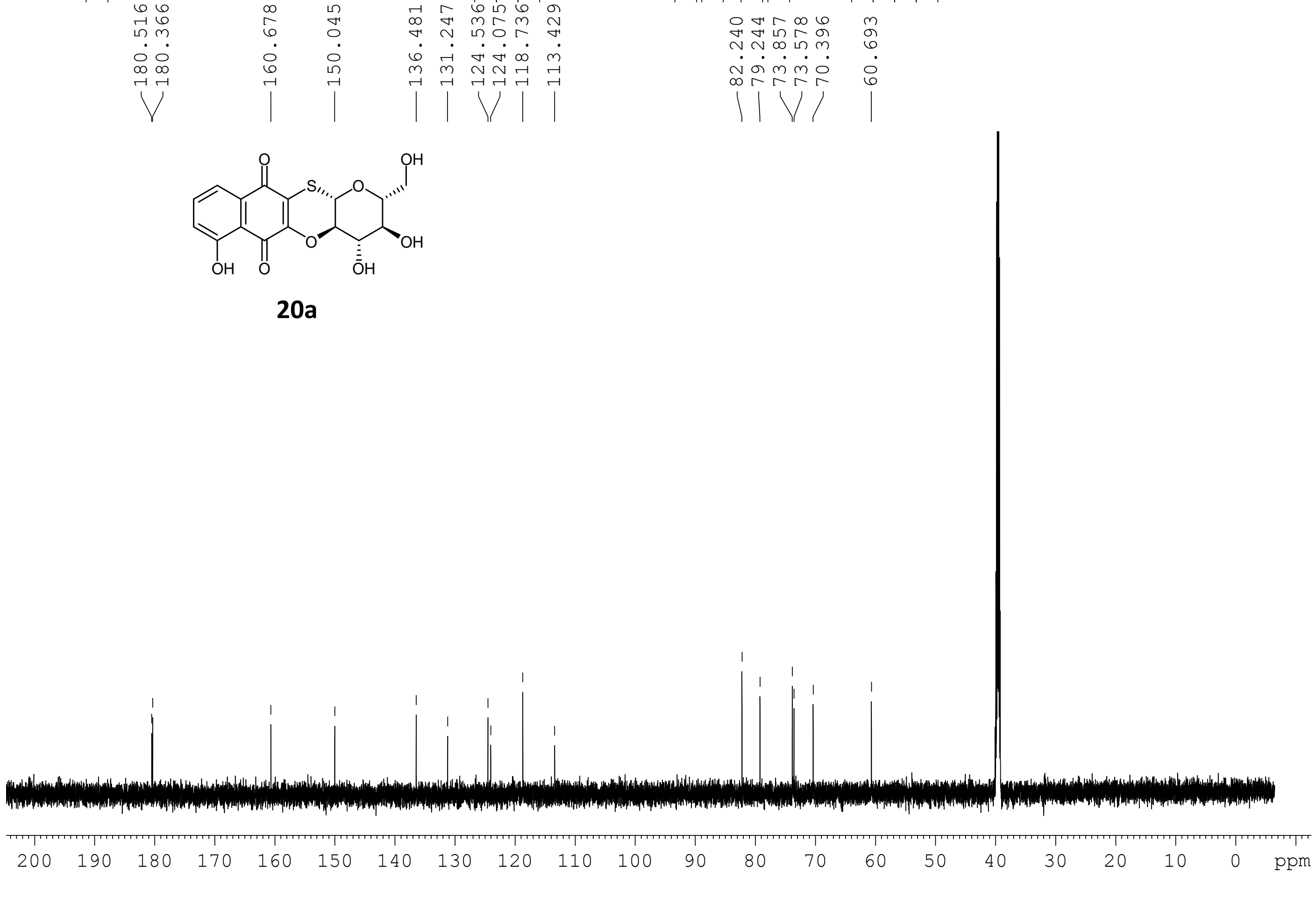

(2R,3R,4S,4aR,12aS)-3,4,7-Trihydroxy-2-hydroxymethyl-3,4,4a,12a-tetrahydro-2H-naphtho[2,3-b]pyrano[2,3-e][1,4]oxathiine-6,11-dione ***(20b)***, *(^{1}H NMR - 500 MHz, ^{13}C NMR - 125 MHz, solvent - DMSO-d$_6$)*

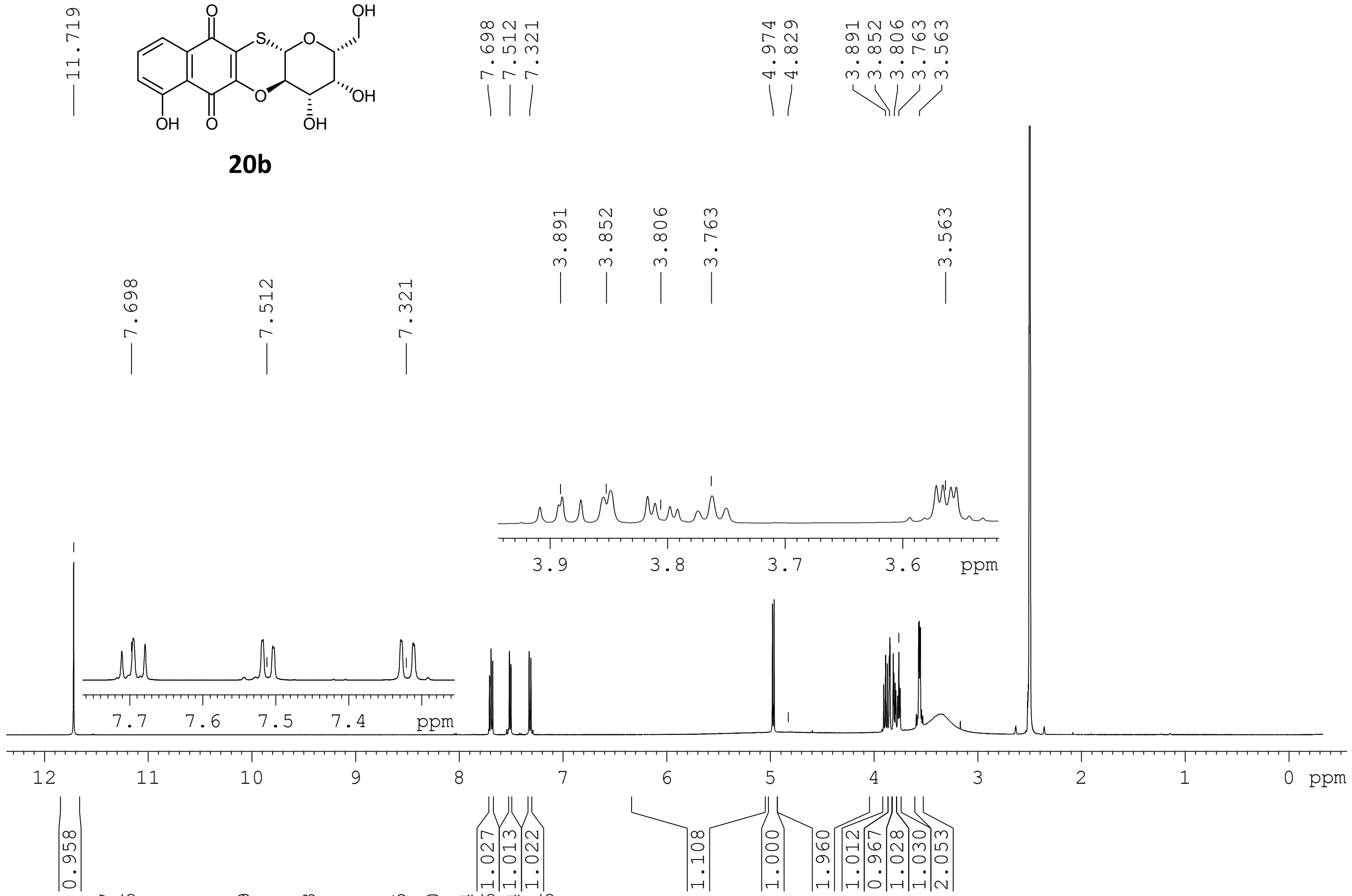

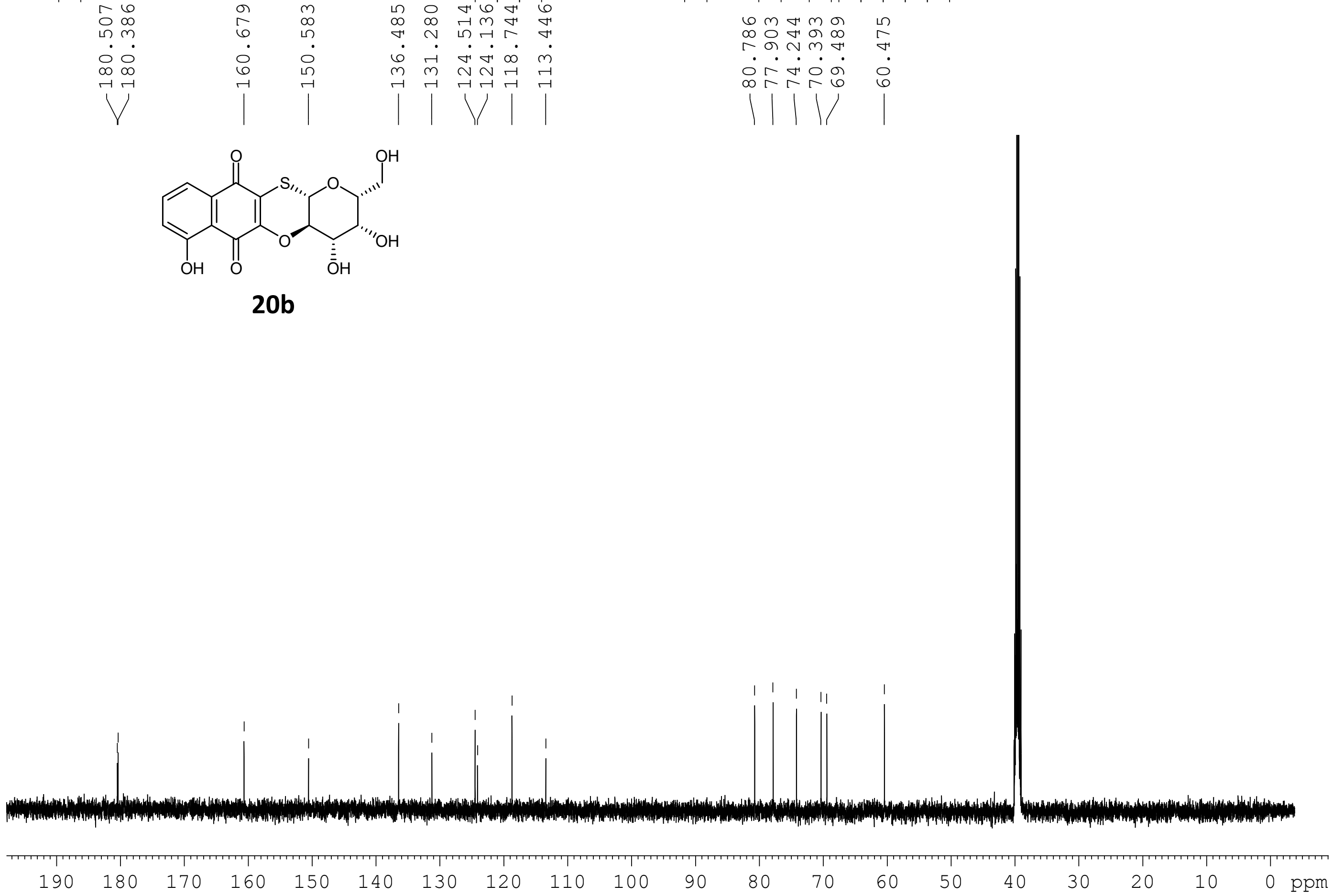

(3R,4S,4aR,12aS)-3,4,7-Trihydroxy-3,4,4a,12a-tetrahydro-2H-naphtho[2,3-b]pyrano[2,3-e][1,4]oxathiine-6,11-dione ***(20c)****, (^{1}H NMR - 500 MHz, ^{13}C NMR - 125 MHz, solvent - DMSO-d$_6$)*

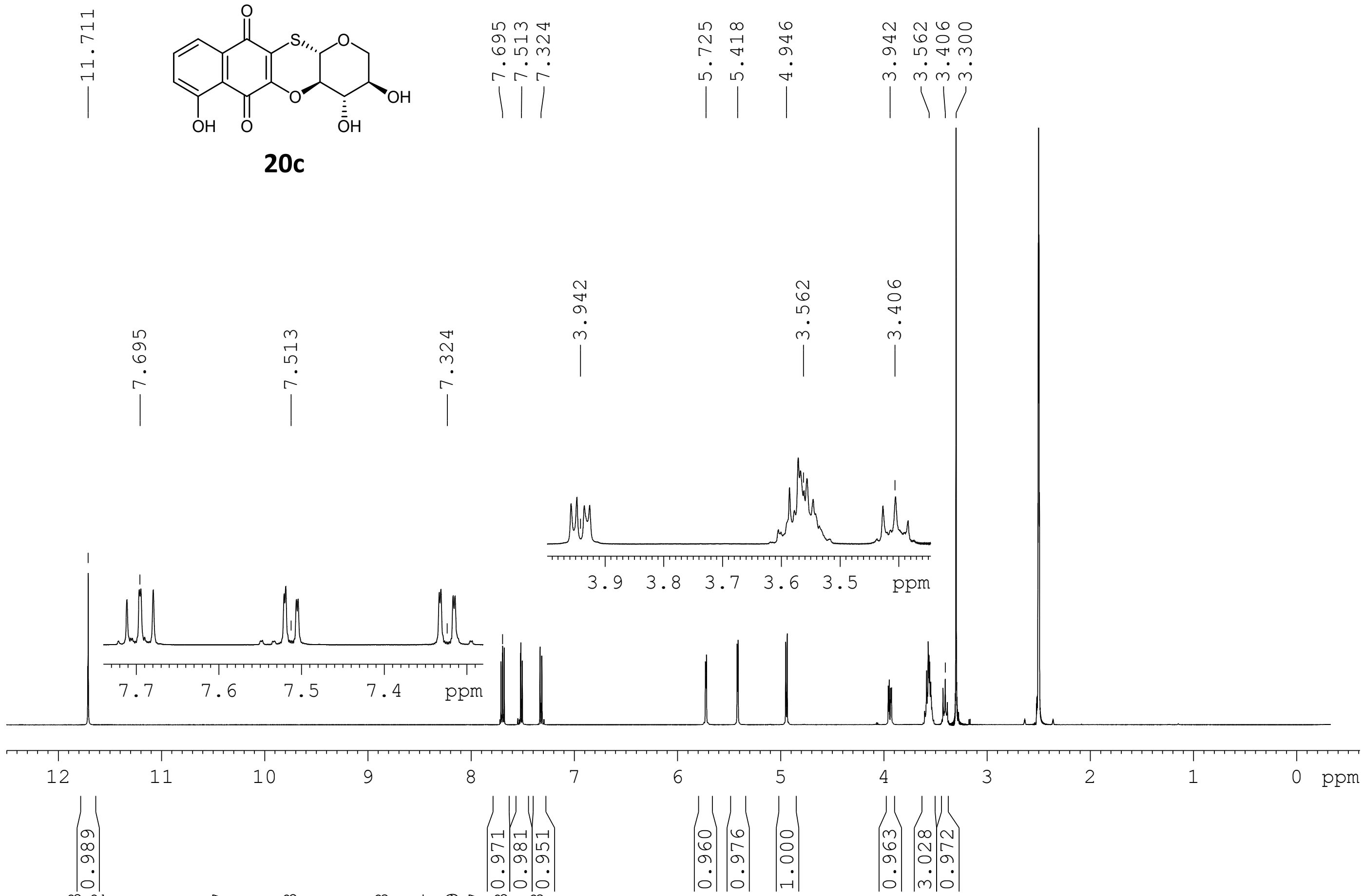

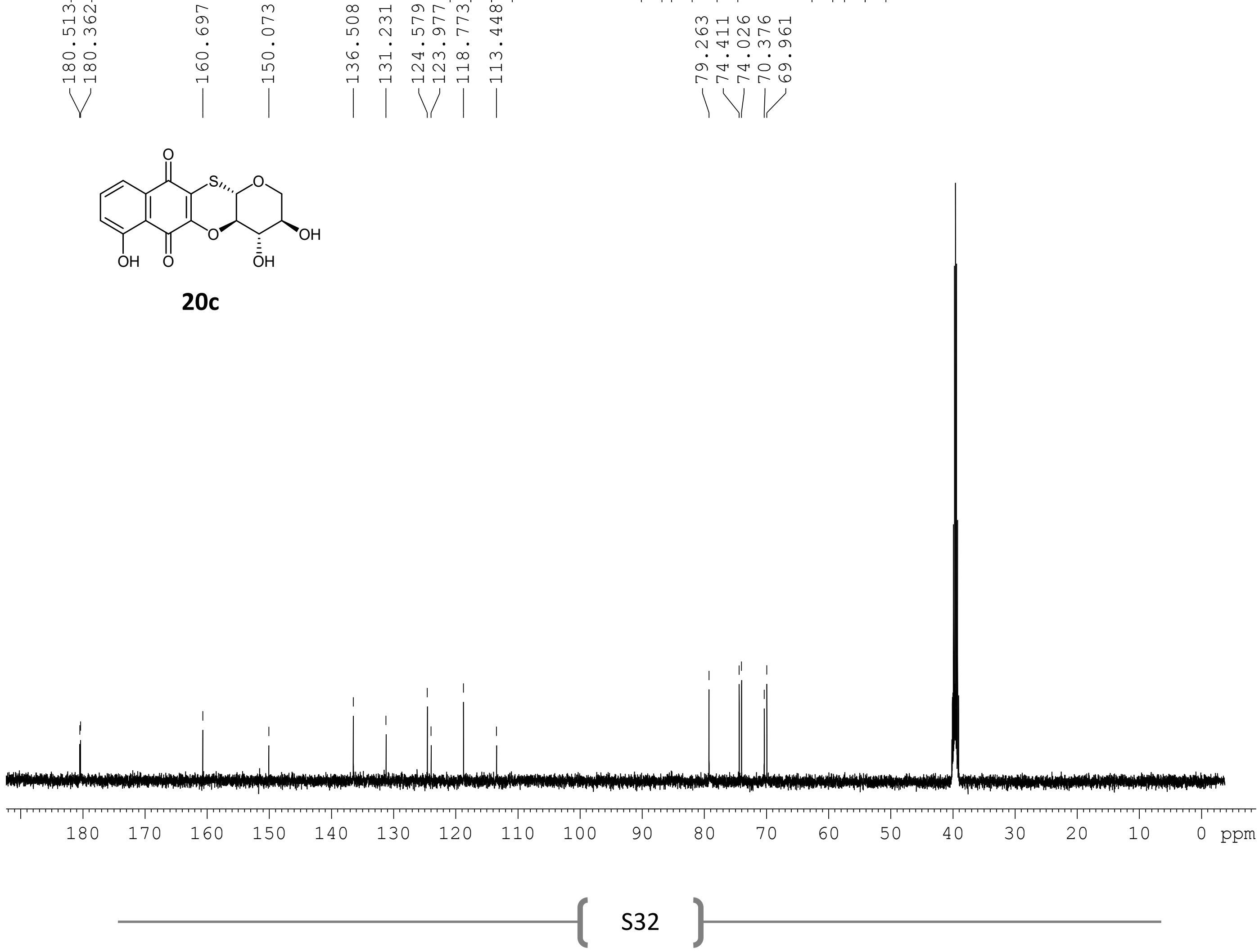

(3S,4S,4aR,12aS)-3,4,7-Trihydroxy-3,4,4a,12a-tetrahydro-2H-naphtho[2,3-b]pyrano[2,3-e][1,4]oxathiine-6,11-dione ***(20d)***, *(^{1}H NMR - 500 MHz, ^{13}C NMR - 125 MHz, solvent - DMSO-d6)*

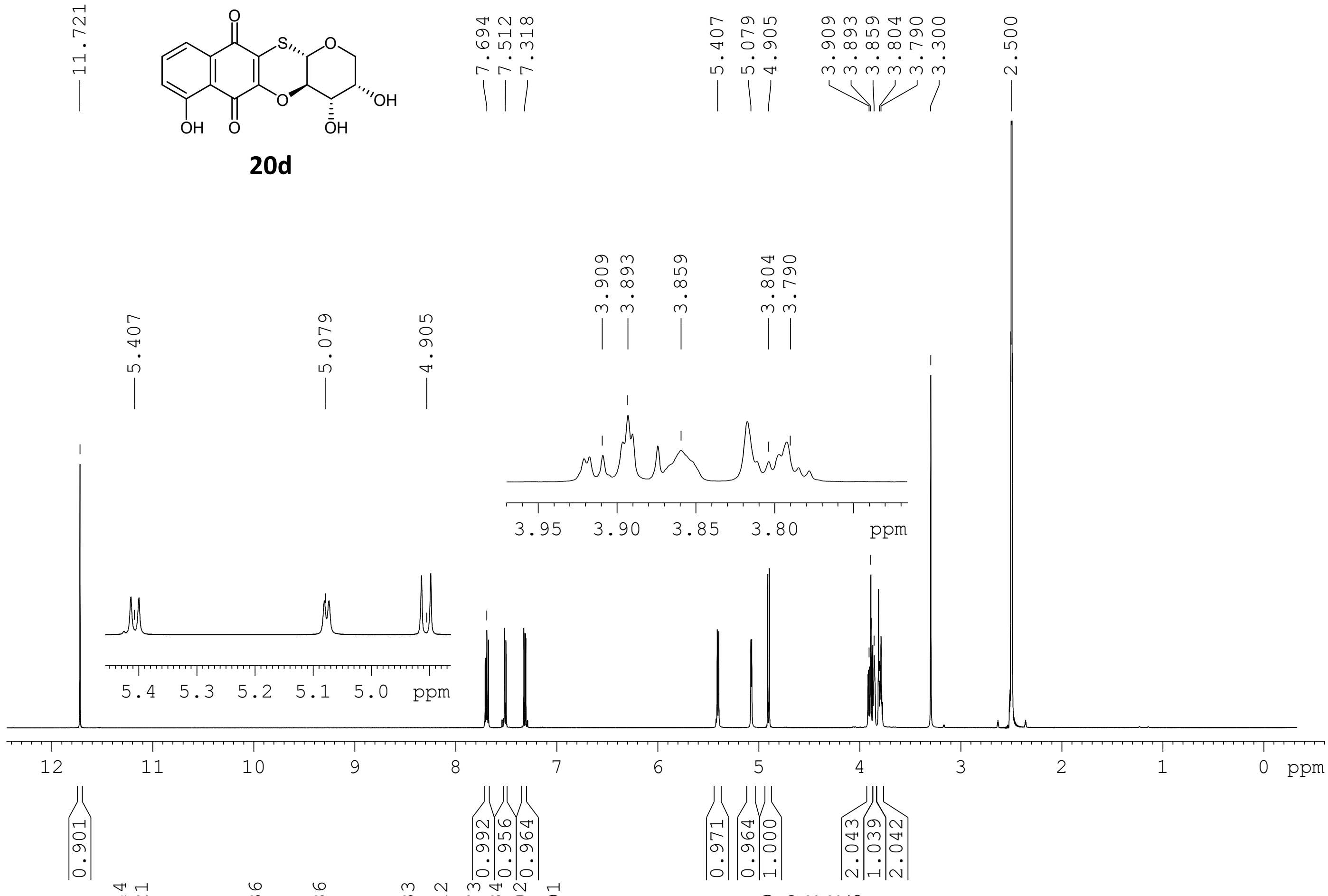

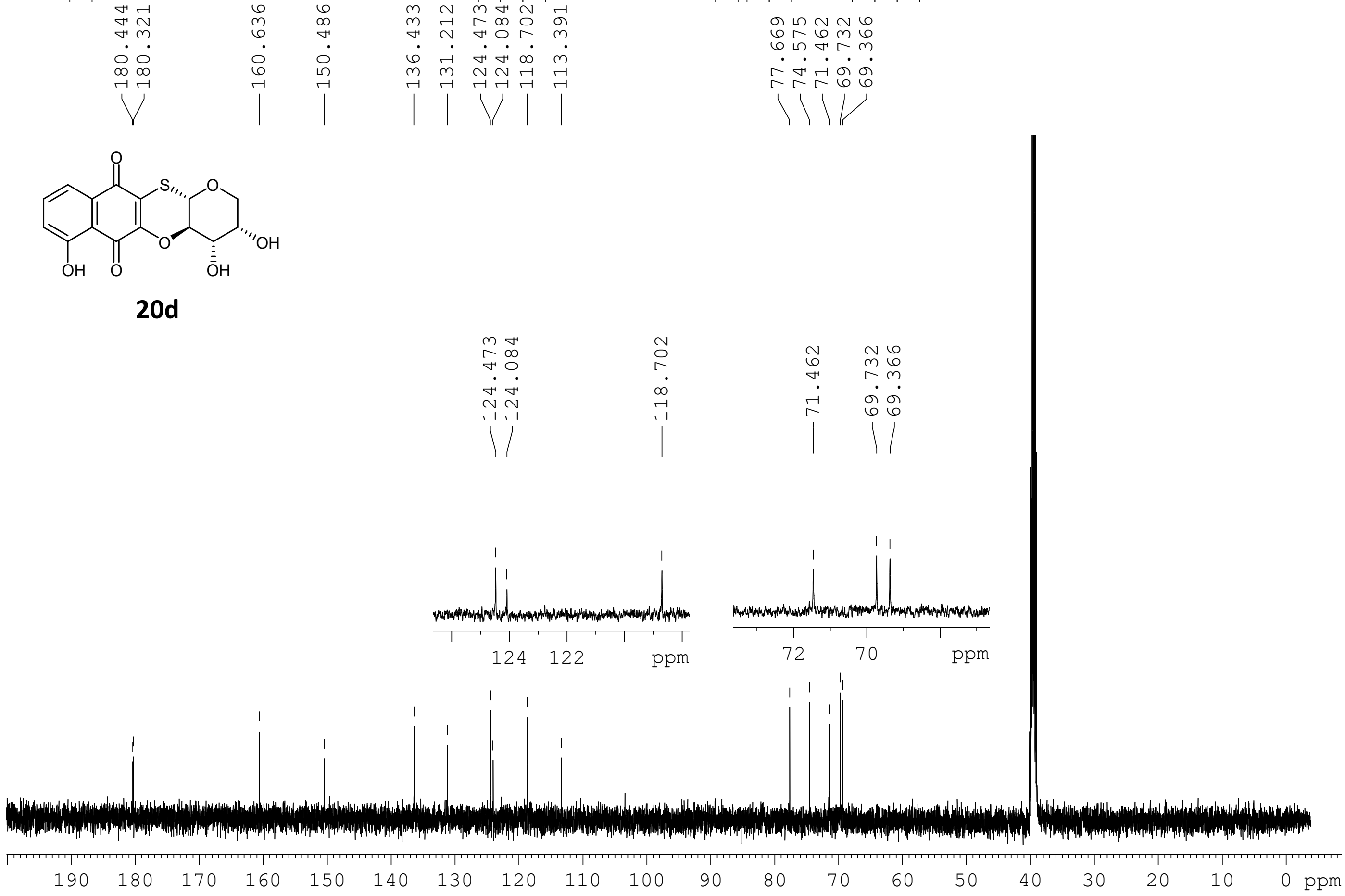

Supplement: Supplementary file 1 [file molecules-25-03577-s001.pdf]
